# Highly selective fluorescent turn-on–off sensing of OH^−^, Al^3+^ and Fe^3+^ ions by tuning ESIPT in metal organic frameworks and mitochondria targeted bio-imaging†

**DOI:** 10.1039/d1ra03078g

**Published:** 2021-08-16

**Authors:** Soumya Bhowal, Arijit Ghosh

**Affiliations:** School of Chemical Sciences IACS Jadavpur Kolkata West Bengal 700032 India bhowalsoumya@gmail.com ghosh.arijit.1234@gmail.com; School of Biological Sciences IACS Jadavpur Kolkata West Bengal 700032 India ghosh.arijit.1234@gmail.com

## Abstract

Herein we report a multifunctional high performance metal organic framework (Zn-DHNDC MOF) based chemosensor that displays an exceptional excited state intramolecular proton transfer (ESIPT) tuned fluorescence turn-on–off response for OH^−^, Al^3+^ and Fe^3+^ ions along with mitochondria targeted bio-imaging. Properly tuning ESIPT as well as the hydroxyl group (–OH) allows Zn-DHNDC MOF to optimize and establish chelation enhanced fluorescence (CHEF) and chelation enhanced quenching (CHEQ) based sensing mechanisms. The MOF benefits from acid-base interactions with the ions which generate a turn-on bluish green fluorescence (*λ*_Em_ 492 nm) for OH^−^, an intense turn-on green fluorescence (*λ*_Em_ 528 nm) for Al^3+^ and a turn-off fluorescence quenching response for Fe^3+^ ions. The aromatic –OH group indeed plays its part in triggering CHEF and CHEQ processes responsible for the turn-on-off events. Low limits of detection (48 nM of OH^−^, 95 nM for Al^3+^, 33 nM for Fe^3+^ ions), high recyclability and fast response time (8 seconds) further assist the MOF to implement an accurate quantitative sensing strategy for OH^−^, Al^3+^ and Fe^3+^ ions. The study further demonstrates the MOF's behaviour in cellular medium by subjecting it to live cell confocal microscopy. Along with a bio-compatible nature the MOF exhibited successful accumulation inside the mitochondria of MCF7 cancer cells, which defines it as a significant bio-marker. Therefore the present work successfully represents the multidisciplinary nature of Zn-DHNDC MOFs, primarily in sensing and biomedical studies.

## Abstract 2

ESIPT tuned fluorescence sensing of OH^−^, Al^3+^ and Fe^3+^ ions and mitochondria targeted bio-imaging by a Zn-DHNDC MOF.

## Graphical abstract

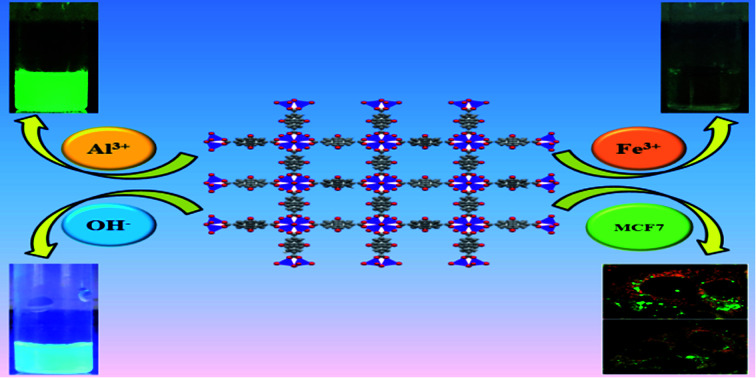

## Introduction

Aluminium (Al) and iron (Fe) are the most abundant (Al ∼ 8.1% & Fe ∼ 5.6%) metals in the earth's crust. These metals in their ionic forms (Al^3+^, Fe^3+^) are not only omnipresent but also vital for the survival of living beings.^1,2^ Fe^3+^ ions mediate several active processes in both plant and animal biological systems such as haemoglobin and myoglobin formation, oxygen transport, mitochondrial electron transport, chlorophyll synthesis, chloroplast maintenance, *etc.*^3,4^ Deficiency of Fe^3+^ ions in the human body can cause reduction of healthy red blood cells or anaemia, whereas excess Fe^3+^ in blood may cause liver and heart diseases, diabetes, *etc.*^5^ Al^3+^ ions, although being present in our food, water and surroundings, do not actively influence the biological system. Excess Al^3+^ may lead to various diseases such as Parkinson's, Alzheimer's encephalopathy *etc.*^6^ Since preventive measurements are always first priority, reliable and accurate techniques for detecting these vital metal ions were highly necessary.

In this report we elaborate on the ESIPT based optical properties of –OH functionalised Zn-DHNDC MOF to create an explicit detection strategy for these highly influential metal ions. MOFs are crystalline, rigid, porous, mono or multidimensional, organic–inorganic hybrid polymers that are constructed with inorganic metal ions and multidentate organic ligands.^7–10^ In recent years applications of MOFs have evolved magnificently, showing potential in various fields such as gas storage^11,12^ and separation,^13,14^ sensing,^15–18^ catalysis,^19,20^ drug delivery,^21,22^ magnetic studies,^23^ proton conductivity,^24–26^ bio-labelling^27^*etc.* Introducing new functional groups in a framework immensely alters the characteristic properties as well as applications.^28^ Optical properties especially fluorescence behaviours are one such aspect that may comprehensively get modified by incorporating chromophoric functional groups.^29^ Such kind of fluorescent MOFs are widely utilized as potential chemical sensors,^30^ photoactive LEDs,^31^ aromatic explosive detectors^32^*etc.* Particle size of the MOFs can be further stabilized in nano region to form nanoscale MOFs (NMOFs).^33^ Fluorescent NMOFs having both particle size and optical properties can be manoeuvred successfully in bio-medical applications as luminescent markers.^34^ High thermal stability, porous geometry and optical properties of MOFs define them as prominent carriers for delivering drugs inside cancer cells.^35^ Progressive research further demonstrates the capability of MOFs acting as contrasting agents in Magnetic Resonance Imaging (MRI).^36^ In photodynamic therapy MOF induced cytotoxic ROS (reactive oxygen species) was exploited to obtain early cancer cell apoptosis.^37^ MOF based selective labelling of organelles such as mitochondria^38^ is one of the current trending topics due to its significant contribution in understanding the proper functioning of the organelles in cellular system.^39,40^ Several MOFs with ESIPT based fluorescent characteristics have been previously reported.^41^ Although MOF based ESIPT tuned sensing mechanisms of metal ions^42^ were rarely explored. To the best of our knowledge this is the first report of a MOF demonstrating various ESIPT tuned mechanisms for sensing multiple ions (OH^−^, Al^3+^ and Fe^3+^) and mitochondria targeted bio-imaging.

Here we report the successful design and applications of –OH functionalized Zn-DHNDC framework synthesized with Zn^2+^ ion and 4,8-dihydroxynaphthalene-2,6-dicarboxylic acid. Structural analysis with powder XRD confirms the cubic IRMOF topology as well as high phase purity and stability of the framework.^43^ Optical properties of the MOF were measured with absorption, emission and fluorescence decay revealed the presence of inherent ESIPT based optical properties. Further investigation of ESIPT in various polar and non-polar solvents exhibited a facilitated ESIPT behaviour in water due to extended polar and H bonding interactions between MOF and solvent medium. Investigation of optical properties in pH 4–10 range demonstrated an ESIPT suppressed intense bluish green fluorescence in higher pH solutions which also represented the pH responsive characteristics of the MOF. A similar ESIPT suppressed intense turn on bluish fluorescence was also observed selectively for OH^−^ ions. When subjected to various metal ions on the other hand the MOF showed a CHEF mediated intense turn on green luminescence for Al^3+^ ions and a CHEQ assisted fluorescence quenching for Fe^3+^ metal ions. With high recyclability, low LOD and fast responsiveness the Zn-DHNDC MOF implements an accurate selective and quantitative detection strategy for these ions. The MOFs potential as a bio-marker was exploited by successful cellular uptake manifesting least cytotoxicity both at pH-7 and pH-9. Further investigation with live cell confocal microscopy exhibited localization of the MOF in mitochondrial region. Therefore the scope of application for the Zn-DHNDC MOF is not only limited to naked eye detection of OH^−^, Al^3+^ and Fe^3+^ ions but it also exceeds in bio-medical field.

## Results and discussion

**Fig. 1:**
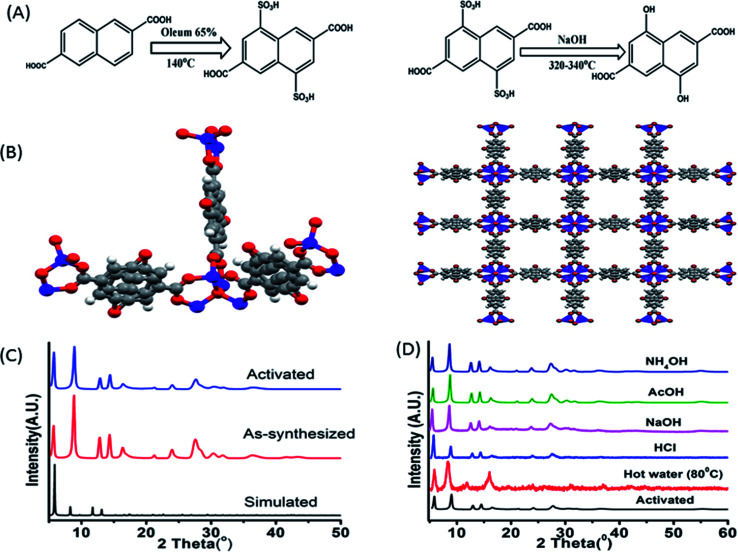
(A) Synthetic scheme of 4,8-dihydroxynaphthalene-2,6-dicarboxylic acid; (B) three dimensional structures of the –OH functionalized Zn-DHNDC MOF drawn from literature report;^43^ the red balls represent the oxygen, grey balls represent carbon and blue balls represent Zn^2+^ atoms; (C) the powder XRD plots of simulated, as-synthesised and activated MOF; (D) PXRD plot of MOF collected from hot water (80 °C), NaOH, NH_4_OH, HCl, AcOH solutions.

The synthetic procedure of the ligand can be divided into two segments, first sulphonation and then hydroxylation. Sulphonation with oleum 65% in 140 °C is a reaction of high yield although being hazardous. The reaction being a ring deactivating one, needs higher temperature and greater time (24 h) to produce the desired disulpho derivative. The second step involving hydroxylation was performed by fusing the disulpho derivative with NaOH at around 310–330 °C in solid state (Fig. 1A). Normal solvo-thermal methods were not applicable for this reaction mainly for two reasons. First, the sulpho group in presence of NaOH became deprotonated (–SO_3_^−^) which was highly co-ordinated by solvent medium, making it almost impossible for OH^−^ nucleophile to replace –SO_3_Na group. Second, the highly stable C–S bond needed greater temperature (around 300–320 °C) to cleave which was not possible to obtain in normal solvo-thermal conditions. Although the ligand possesses two carboxylic acid (–COOH) and two hydroxyl functional groups (–OH), construction of the Zn-DHNDC framework involves –COOH group chelating with Zn^2+^ to form the cubic framework leaving the –OH group free in the pores. In general the presence of proton containing group such as –OH, –NH_2_*etc.* alongside a proton accepting group such as –C=C, –C=N generates keto–enol tautomerism. Therefore upon irradiating such compounds, rapid proton transfers between the –OH group and aromatic –C=C group takes place which lead to the ESIPT properties.

The synthetic procedure yielded stable cubic orange-red crystals. The structure was confirmed by matching the experimentally obtained as-synthesized and activated PXRD data with the simulated diffraction patterns.^43^ The PXRD data presented in Fig. 1C presenting a well matched diffraction pattern further confirms the classical IRMOF-8 topology. The MOF crystals possess a highly symmetrical cubic geometry with space group *Fm*3̄*m*.^43^ The SBU Zn_4_O(−CO_2_)_6_ is composed of four tetrahedral Zn^2+^ metal ions linking with one μ_4_ oxygen and 6 ligand carboxylates groups. Thus the Zn_4_O metal ion cluster act as a 6 point connected node while the ligand acts as a 4 connected node. The (6, 4) bonding between ligands and SBU nodes propagate along all three axes resulted the 3D framework. The framework contained cubic channels as pores filled with solvents, moisture and free –OH group. The stability of the MOF in various chemical environments were investigated by performing PXRD of the MOF recovered from hot water, NaOH, HCl, AcOH, NH_4_OH solutions. Fig. 1D constitutes a similar curve nature, good stability of the peaks and intensity of all the recovered MOF samples which further confirmed the structural robustness of the MOF. Thermo gravimetric analyses of the as-synthesised and activated MOF revealed their corresponding thermal stabilizations. The as-synthesized MOF showed a stepwise weight reduction with increasing temperature. At first a weight loss of 15% at around 60–95 °C, 14% at around 170–230 °C was observed. These two steps represent the evaporation of ethanol, moisture and DMF from the pores of the MOF. A considerable weight loss of around 24% was observed at 380–482 °C which signified the degradation of the framework. The activated MOF on the other hand showed a plot with a single step in the region of 360–500 °C signifying the framework degradation. Therefore the stability of the framework is up to 360 °C (Fig. 3S†). The FT-IR data of the as-synthesized, activated MOF are exhibited in Fig. 4S.† The figure represents two wide peaks at around 3135 and 3460 cm^−1^ which can be assigned as the O–H stretching frequency of the phenolic hydroxyl group and solvent molecules respectively.

The H-bonding interaction between the solvent molecules, moisture with linker –OH group broadens the peak representing O–H stretching. Other important peaks characterizing the ligand of the MOF are the peak at 1670 cm^−1^ representing the symmetric stretching frequency of carboxylate, 1410 cm^−1^ representing the asymmetric stretching of carboxylate, 1202 cm^−1^ representing O–H bending, 1047 cm^−1^ representing C–OH stretching frequency, 797 cm^−1^ representing C=C bending *etc.* The sharp peak at around 628 cm^−1^ can be assigned to metal–oxygen stretching frequency which represents a successful formation of the framework (Fig. 3S†). Transmission electron microscopy (TEM) micrographs exhibited in Fig. 5S† represents agglomerated cubic crystalline MOF particles in nano region. The particle size of the MOF resides in the range of 25–50 nm. Moreover HRTEM images display an arrangement of lattice fringes representing the overlapping of crystal lattice planes. The selected area electron diffraction (SAED) image displays a definite array of white spots representing the diffraction of the unscathed crystalline MOF particles in nano region. The Energy-Dispersive X-ray spectroscopy (EDX) of such nano range MOF particles constitutes the distribution of all the elements of the MOF (Fig. 5S†) and thus confirming the stability of the MOF in nano region. The particle size in a solution phase was determined with dynamic light scattering (DLS). The hydrodynamic diameter was found around 78–105 nm (Fig. 6S(E)†). A high solvent-MOF interaction may attribute for higher particle size observed from DLS compare to TEM. The zeta potential of the MOF was observed around −8 mV (Fig. 20S†).

### Optical properties of MOF

Spectroscopic methods are the most prominent techniques for measuring the ESIPT based optical properties. These methods involve absorbance (UV-VIS), emission (Photoluminescence, PL), time-correlated single photon counting (TCSPC) *etc.* The absorbance of the MOF showed a strong peak around 248 nm, three small humps at 287, 299, 312 nm and a broad peak at 363 nm respectively. The ligand also displayed a similar curve nature with a slight shift towards red region (Fig. 6S†). The initial four peaks residing at 248, 287, 299, 312 nm represent the π–π* transition while the broad peak at 360 nm can be assigned as the n-π* transition.^15^ The TCSPC was used to determine the fluorescence decay and average lifetime of the MOF. The curve displayed a bi exponential decay nature with average lifetime of around 2.97 ± 0.119 ns (Fig. 6S†). The emission of the ligand showed two broad peaks with almost similar width and intensity having *λ*_Em_ around 414 and 536 nm situated over a wide (364–600 nm) range (exited with 350 nm light). The MOF also exhibited a two headed broad emission curve having *λ*_Em_ around 410 and 503 nm situated at the same wide range (*λ*_Ex_ = 350 nm). But for MOF the peak on 410 nm possess a higher intensity than the other one (Fig. 6S(B)†). The MOF also showcased a considerable Stokes shift of 50 nm. Therefore observations such as a Stokes shift along with a wide two headed curve nature situated over blue to red range only suggested the presence of additional exited state activities or ESIPT phenomenon. The peak situated at lower (*λ*_Em_ 410 nm) and higher wavelength region (*λ*_Em_ 503 nm) can be assigned to the enol and keto tautomer respectively.^44,45^ The presence of ligand to metal charge transfer (LMCT) between ligand to d^10^ metal ion (Zn^2+^) is the reason behind the enhancement of the 410 nm peak. For d^10^ metal ions such as Zn^2+^, Cd^2+^ based MOFs, such LMCT between carboxylate oxygen orbitals (p) to vacant metal ion orbital (s) is a common occurrence. Although d^10^ metal ion based MOFs were previously reported to show such LMCT behaviour,^46^ the ESIPT phenomenon is associated with –OH group modification.^47^ Therefore observations conclude that optical properties of the Zn-DHNDC MOF are heavily influenced by the –OH inclusion in the framework.

**Fig. 2:**
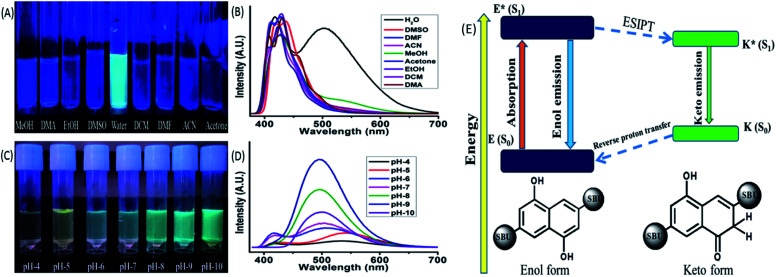
(A) Fluorescence of MOF (365 nm light) in different solvents such as MeOH, DMA, EtOH, DMSO, water, DCM, DMF, ACN, acetone; (B) the PL spectra of MOF in the abovementioned solvents; (C) fluorescence of MOF (365 nm light) in pH 4–10 solutions; (D) the PL spectra of MOF in pH 4–10. (E) Energy level diagram representing ESIPT and keto–enol tautomers of the MOF.

The ESIPT phenomenon was further investigated in different polar and non-polar solvents. The experiments was conducted by dispersing 1 mg of the MOF in 3 mL of solvents such as water, methanol (MeOH), ethanol (EtOH), acetone, dimethylformamide (DMF), dimethyl sulphoxide (DMSO), dichloromethane (DCM), dimethylacetamide (DMA), acetonitrile. The solutions were sonicated for 20 min and rested for 30 min before estimating of the optical properties. In 365 nm light the MOF showed a faint bluish green fluorescence in water while in the rest of the solvent solutions displayed slight bluish or no fluorescence at all (Fig. 2(C)). The PL data of the MOF in different solvents exhibit diverse emission spectra nature (Fig. 2(D)). The MOF showed a wide curve with a single peak having *λ*_Em_ at around 430 nm in dichloromethane. In presence of water, acetone, acetonitrile, DMA, MeOH, EtOH, DMF, DMSO the MOF displayed two headed curves which indicated the existence of enol and keto tautomer. MOF in acetone and acetonitrile solutions showed a similar type of curve nature with a slight shift in position. The *λ*_Em_ of enol tautomer was found around 423 and 425 nm and keto was around 407 and 410 nm respectively. DMF and EtOH solution of MOF also showed a similar curve nature and situated in an almost same wide region having *λ*_Em_ around 410 and 430 nm respectively. In DMA the MOF produces two distinctly separate peaks situated at 408 and 430 nm. In this case the two peaks representing enol and keto are situated 22 nm apart from each other. The MOF in DMSO showed a slight red shifted curve with two less separated peaks resembling the nature of EtOH and DMF. In DMSO the *λ*_Em_ of the MOF was observed at 419 and 436 nm respectively. In MeOH, the MOF showed a broad peak with *λ*_Em_ at 428 nm and a wide hump having *λ*_Em_ around 540 nm.

As discussed earlier the MOF in water exhibits two prominent peaks having *λ*_Em_ at around 410 and 503 nm. The MOF showing a blue shifted obscure peak nature in solvents such as acetone, acetonitrile, DMF and ethanol only suggest a weak interaction between MOF and solvent medium due to which ESIPT characteristics gets hindered. The slight red shifted curve in DMSO along with well defined curve natures in methanol, water and DMA are due to presence of internal H bonding and increasing polar interactions between MOF and solvents that stabilize the ESIPT behaviour. Both ligand and MOF showing two properly separated peaks only reflects the stability of these components in water medium due to such interactions. In MeOH, the wider peak created at 540 nm suggested the presence of intermolecular H bonding but compare to water it is weak. MOF in DCM fails to show the traditional behaviours of ESIPT due to absent of such interactions. The MOF in ground state reside in enol form. When irradiated the ligand also form the keto tautomer due to rapid proton transfer between the two isomers which produce the two headed emission curve. The keto tautomers in water are further stabilized by severe intermolecular interactions. This coupled with the stability of MOF found with PXRD (Fig. 1D) makes it a suitable medium for further detection experiments.^44,45,47^

### pH responsiveness of the MOF

In a similar method the pH responsiveness of the MOF was measured by subjecting the MOF (1 mg) to different pH solutions ranging from pH 4–10. In 365 nm light (Fig. 2(C)) as the pH increased the fluorescence colour of the MOF in these mediums changed from faint yellowish green to intense bluish green. The PL curves of the MOF in pH 4–10 range (Fig. 2D) demonstrated a rapid increase in intensity in pH 4–9 range while a sudden drastic reduction of intensity at pH-10. In acidic pH solutions (pH = 4, 5) the intensity of the MOF was the lowest and the overall curve was comparatively red shifted with the keto *λ*_Em_ around 550 nm. In pH 6 and 7 the overall fluorescence intensity increases with the curve shifting a bit towards blue region. For pH solution 8 and 9 the two headed curve nature change into a wide curve with single peak. Therefore overall a blue shift of curve was observed with increasing pH medium along with the enhancement of intensity. To observe the influence of acidic and basic medium on the optical properties separately the Zn-DHNDC MOF was subjected to PL titration with 10^−3^ N NaOH and HCl solution were obtained (Fig. 8S†). For titration with NaOH, the fluorescence intensity of the MOF enhanced quite a fold. With gradual addition of NaOH the two headed curve changes into a single headed peak and shifts slightly towards the blue region at saturation point. With gradual addition of HCl on the other hand the fluorescence intensity of the MOF decreased rapidly. In this case gradual addition of HCl caused the overall PL curve to move toward red region as the two headed curve nature remained intact.

Certain conclusions can be made from these experiments. The PXRD represented a good stability of the MOF in HCl and NaOH medium which was previously established. The acidic protons of –OH group are the key component in bestowing the ESIPT phenomenon in the MOF. ESIPT often leads to reduction of fluorescence intensity. In high pH solutions and base titrations the acidic protons interact with the base medium only to form deprotonated MOF.^48^ Due to such deprotonation the ESIPT and keto–enol tautomerism was suppressed.^48^ Therefore the two headed curve nature transforms into a curve with single peak. The deactivation of ESIPT causes reduction of Stokes shift which furthermore resulted in the blue shift of the emission curves. Due to the deprotonation the delocalised π electron density inside naphthalene aromatic ring increased quite a fold. This phenomenon also increased the charge transfer from filled ligand orbitals to vacant Zn^2+^ metal orbitals.^46^ Due to this reason a comparatively blue shifted intense PL was recorded in basic medium which was the reason for the bluish green fluorescence. In acidic medium on the other hand fluorescence intensity decreased due to the quenching nature of H^+^ ions. The ESIPT on the other hand get facilitated in acidic conditions, shifting the curve towards red region. Therefore in lower pH solutions and acidic medium the MOF showed a faint yellowish green fluorescence. In pH-10 due to presence of strong basic conditions the MOF dissociates which is responsible for the sudden drop in fluorescence intensity. The PXRD of MOF collected from pH-9 and pH-10 buffer solutions also project a similar outcome (Fig. 25S†). Therefore the pH responsive fluorescence property of the MOF is highly influenced by ESIPT characteristics. The optimum stability of MOF is found to be at pH-9. The zeta potential of the MOF at pH-7 is −8.10 mV while at pH-9 it is −19.2 mV. This phenomenon also hints the presence of deprotonation in presence of higher pH solutions (Fig. 36S†).

### Influence of anions

**Fig. 3:**
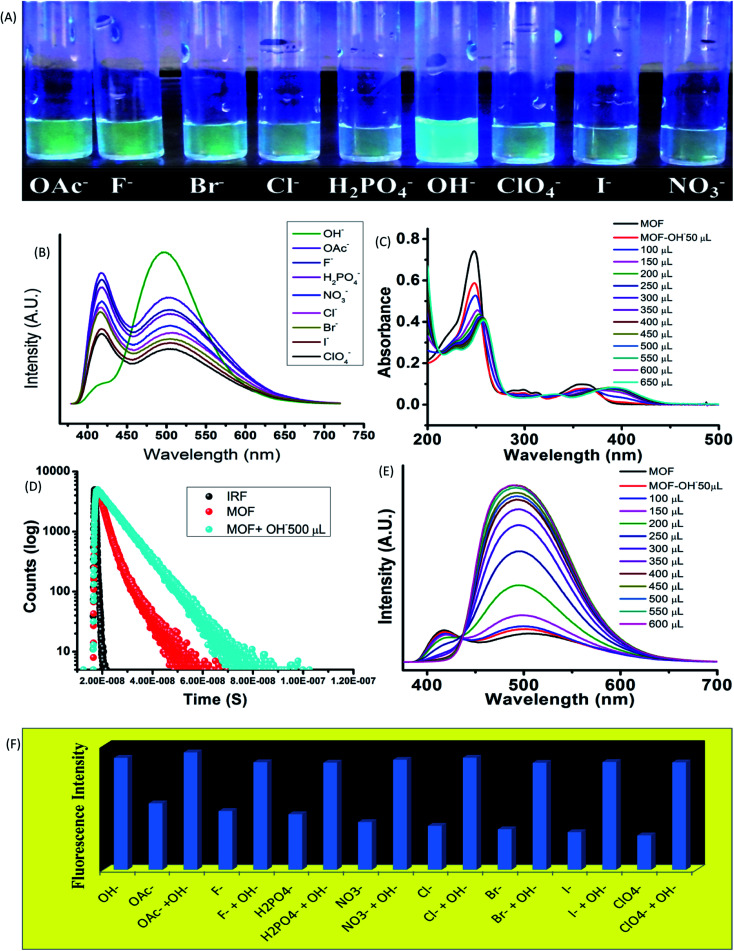
The optical properties of MOF in presence of various anions. (A) The fluorescence of the MOF in presence of anions in 365 nm light; (B) represents the emission data of the MOF in presence of various anions; (C) UV-VIS titration of the MOF with OH^−^ ions; (D) the PL titration of the MOF with OH^−^ ions; (E) the fluorescence decay of MOF and the MOF in presence of OH^−^ ions; (F) fluorescence intensity of MOF in anions and anion-OH^−^ ion mixture.

The influence of anionic components on the optical properties was measured by subjecting the MOF (1 mg) in 10^−3^ M aqueous solution of tetra butyl ammonium (TBA) salts (F^−^, Br^−^, Cl^−^, I^−^, OH^−^, ClO_4_^−^, OAc^−^, NO_3_^−^, H_2_PO_4_^−^) of different anions in aqueous medium. In 365 nm light the MOF showed an intense bluish green fluorescence selectively in presence of hydroxyl (OH^−^) ions (Fig. 3A). The rest of the solutions did not exhibit any alterations of the usual fluorescence of the MOF. The primary investigation was carried out by obtaining absorbance of MOF in presence of different anions (Fig. 9S†). Apart from OH^−^ ion, none of the other anions cause any effective change in UV-VIS nature of the MOF. In presence of OH^−^ a red shift of the curve was observed as seen in Fig. 3C.

UV-VIS titration with OH^−^ ion displayed a gradual red shifting of the curve until saturation point is reached. At saturation point the pH of the solution remain in 9.2, the π–π* peak shifted up to 257 nm and the n-π* peak stabilized at around 393 nm with addition of around 500 μL of 10^−3^ M OH^−^ ions. Equivalent amount of OH^−^ ions are added individually to all the anion@MOF solutions. The absorbance plots of all the solutions exhibit a similar bathochromic shift resembling the OH^−^@MOF solution. Next the emission properties were obtained by conducting PL titrations of the MOF with all the anionic solutions (10^−3^ M) (Fig. 11S†). In a similar scenario the MOF only showed a rapid change only in presence of OH^−^ ions. Titration with F^−^, OAc^−^ solutions shows a slight enhancement in intensity (Fig. 11S†). As observed earlier during the PL titration with NaOH the OH^−^ titration also showcased an enhancement of fluorescence intensity along with transformation of ESIPT induced two headed curve nature (Fig. 3E). At saturation point (pH-9.2) the MOF displayed around 6 fold enhancement of fluorescence intensity with addition of 500 μL of 10^−3^ M OH^−^ solution. Selectivity was obtained by adding equivalent amount of OH^−^ to all the anion@MOF solutions. The PL spectra of all the solutions showed a similar curve nature to OH^−^@MOF. The Fluorescence decay measurements in presence of OH^−^ recorded an increase of average lifetime of the MOF. With addition of 500 μL of 10^−3^ M OH^−^ the average lifetime of the MOF increased up to 6.7 ± 0.376 ns (Fig. 3D). The limit of detection (LOD) is measured by using the equation 3*σ*/*K* where *σ* is the standard deviation and *K* is the slope of the fluorescence titration curve. The LOD is calculated to be 4.83 × 10^−8^ M (48 nM) (Fig. 33S†). To obtain the recyclability of the MOF it is subjected to OH^−^ in 5 cycles. The first cycle consisted of obtaining the emission of MOF suspended in OH^−^ ion solutions. The MOF is then recollected with centrifugation and washed with water to remove the OH^−^ ions from the surface of the MOF. After measuring the emission of the recovered MOF it is then again subjected to OH^−^ ion solutions for the second cycle. All the 5 cycles showcased fluorescence enhancement (Fig. 34S†) although the fifth cycle featured intensity reduction of around 10%. The loss of intensity could be due to partial degradation of the MOF. The PXRD of the MOF recovered from the 5^th^ cycle showed a good stability of the peaks. The exposure time was obtained by conducting a time dependent sensing with 300 μL of OH^−^ anion. Almost 4–4.5 fold enhancement of fluorescence intensity was observed within 5 second time range. Also within 750 second around 5 fold increase of intensity was recorded (Fig. 35S†).

Higher pH solutions generate OH^−^ ions in aqueous medium. The OH^−^ ions being strong base, easily deprotonated the MOF and increase the LMCT between ligand and Zn^2+^ ions. Other anions such as OAc^−^ and F^−^ although possessing strong base nature could not alter the emission nature of the MOF apart form a slight enhancement of intensity. The rest of the anions with comparatively weaker base nature did not cause any effect on emission of the MOF. Therefore the selectivity lies in the basicity of the OH^−^ ions as it is the only ion strong enough to suppress ESIPT. The zeta potential of the MOF in presence of different anion solutions also exhibit deprotonation. A greater negative zeta potential than the MOF in neutral water was observed in presence of anionic solutions. In presence of anions with stronger base nature such as OH^−^, OAc^−^ the potential is lowest (−24.6 & −19.6 mV) while for anions with weaker base properties such as Br^−^, I^−^, ClO_4_^−^ ions the potential stays at –10 to −13 mV range. Therefore observations conclude that most of the anions are capable of deprotonating the MOF. The degree of deprotonation for anions with weaker base strength is very less to almost nothing. Anion with stronger base nature however had a higher degree of deprotonation which lead to the potential becoming greatly negative. To the best of our knowledge this is the first report on an ESIPT based MOF selectively sensing hydroxide ions in aqueous medium along with high pH mediums.

### Metal ion detection properties

**Fig. 4:**
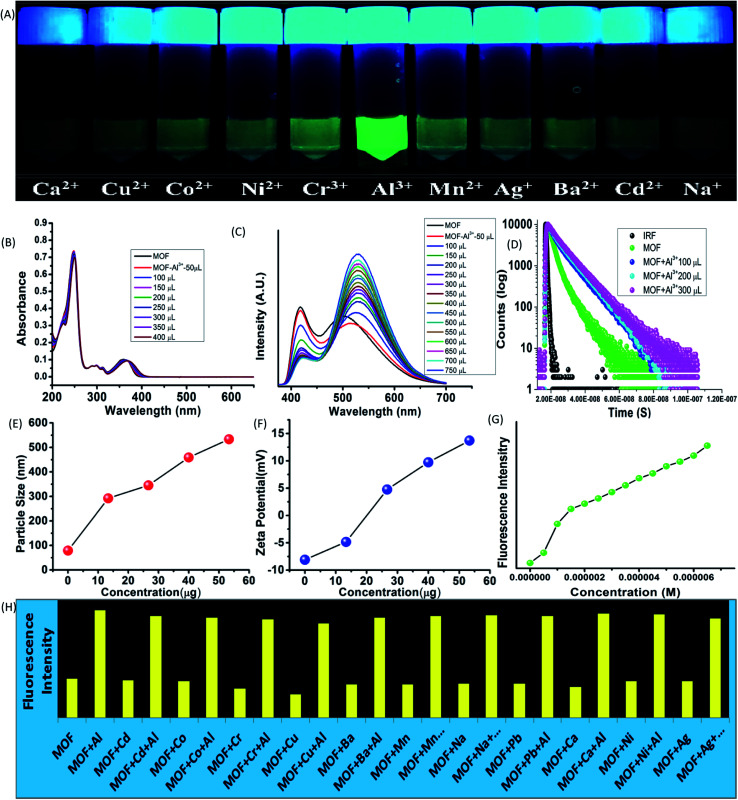
Optical properties of MOF in presence of metal ions and Al^3+^ ion sensing. (A) Fluorescence of MOF in presence of different metal ions in 365 nm light; (B) UV-VIS titration of MOF by Al^3+^ ions; (C) PL titration of MOF by Al^3+^ ions; (D) fluorescence decay titration of MOF by Al^3+^ ions. (E) Dynamic light scattering (hydrodynamic diameter size) titration of MOF by Al^3+^ ions. (F) Zeta potential titration of MOF by Al^3+^ ions; (G) fluorescence intensity of MOF in presence of Al^3+^ ions; (H) fluorescence intensity of MOF in presence different metal ions and metal ion-Al^3+^ solution.

While interactions with anions represents the acidic properties of the –OH group, the MOF also possess a Lewis base character. The basic properties of the –OH group were further exploited by subjecting the MOF in various metal ion solutions. 1 mg of MOF was added to 1 mL of 10^−3^ M aqueous solutions of salts containing different metal ions such as Fe^3+^, Cr^3+^, Co^2+^, Ni^2+^, Mn^2+^, Cd^2+^, Mg^2+^, Cu^2+^, Ag^+^, Al^3+^, Ba^2+^, Na^+^ (all chloride salts) and pH-7 solution in glass vials. At 365 nm light the MOF exhibited an intense green fluorescence in presence of Al^3+^ ions while for Fe^3+^ ions the MOF showed no fluorescence at all. Rest of the metal ion solutions of MOF did not project any alterations in optical properties (Fig. 4A). All the following sensing experiments were conducted in pH-7 to maintain a uniform medium.

### Al^3+^ sensing

At first the UV-VIS of the MOF in presence of all the above mentioned metal ions were obtained. In presence of Al^3+^ the UV-VIS curve exhibited an overall red shift, especially the n-π* peak. The UV-VIS titration with 10^−3^ M Al^3+^ displayed a slight bathochromic shift of the π–π* peak up to 250 nm with a slight decrease of intensity. The n-π* peak on the other hand stabilized at 367 nm (Fig. 4B) in saturation point. In presence of other metal ions except Fe^3+^ the MOF did not show any alterations of UV-VIS nature. Therefore to obtain the selectivity equivalent amount of Al^3+^ ion was added to all the metal ion solutions of the MOF. The absorbance of all the solutions showed a bathochromic shift and curve nature similar to Al^3+^@MOF solution. The PL of the MOF in presence of all metal ions also provided a similar outcome. In presence of Al^3+^ ions, the PL intensity of the MOF enhances quite a fold (*λ*_ex_ – 350 nm). The overall curve shifted toward red region but in this case also the ESIPT induced two headed curve nature transformed into a curve with single peak (Fig. 4C) at saturation point.

The PL titration with 10^−3^ M Al^3+^ solution displayed an initial bathochromic shift of 24 nm and slight enhancement of the keto peak with addition of 50 μL of Al^3+^ solution. Then gradual addition of Al^3+^ solution caused the intensity of the enol peak to reduce while the keto peak increases prominently. With addition of around 650 μL of 10^−3^ Al^3+^, the MOF showed around 3 fold increase of intensity. At saturation point the *λ*_Em_ of the Al^3+^@MOF solution is stabilized at 528 nm. In this case also apart from Fe^3+^ ions other metal ions did not cause any alterations in emission nature of MOF. To obtain the selectivity, equivalent amount of Al^3+^ was added to all the MOF-metal ion solutions and subjected for PL. The PL for all the solutions except Fe^3+^ featured a turn on fluorescence similar to Al^3+^@MOF (Fig. 17S†). The fluorescence decay of the MOF in presence of Al^3+^ showed an increase of average lifetime. Addition of 500 μL of Al^3+^ exhibited an increase in average lifetime up to around 7.52 ± 0.822 ns (Fig. 4D). The Stern–Volmer constant (*K*_sv_) was calculated according to the literature.^49^ The Stern–Volmer constant (*K*sv) is 1.65 × 10^5^ M^−1^ for the enhancement of fluorescence (Fig. 26S†). The LOD is calculated as 9.52 × 10^−8^ M (95 nM) (Fig. 31S†) which fall among one of the lowest reported LODs for Al^3+^ ions.^49–52^

The particle size and potential were also altered by the presence of Al^3+^ ion. The hydrodynamic diameter of MOF is observed around 78–105 nm with an average diameter of 89 nm. With addition of 400 μL of 10^−3^ M Al^3+^ the average diameter of the MOF particles increased up to around 530 nm (Fig. 4E). The aqueous dispersed MOF showed a zeta potential of around −8.1 mV. With addition of 400 μL of 10^−3^ M Al^3+^ the potential increased up to 13.7 mV (Fig. 4F). Again the reusability for Al^3+^ sensing was obtained by conducting the recyclability test for 5 cycles. The fluorescence intensity of Al^3+^@MOF remained stable throughout the 5 cycles (Fig. 34S†). The PXRD of the MOF recovered from the fifth cycle also showed good peak stability (Fig. 25S†) also corroborates with the recyclability experiment. The exposure time measured with 300 μL of Al^3+^ solution showed 1.7–1.9 fold enhancement of fluorescent intensity within 8 second. In 850 second the Al^3+^@MOF solution showed up to 2 fold enhancement of fluorescence (Fig. 35S†).

### Fe^3+^ sensing

**Fig. 5:**
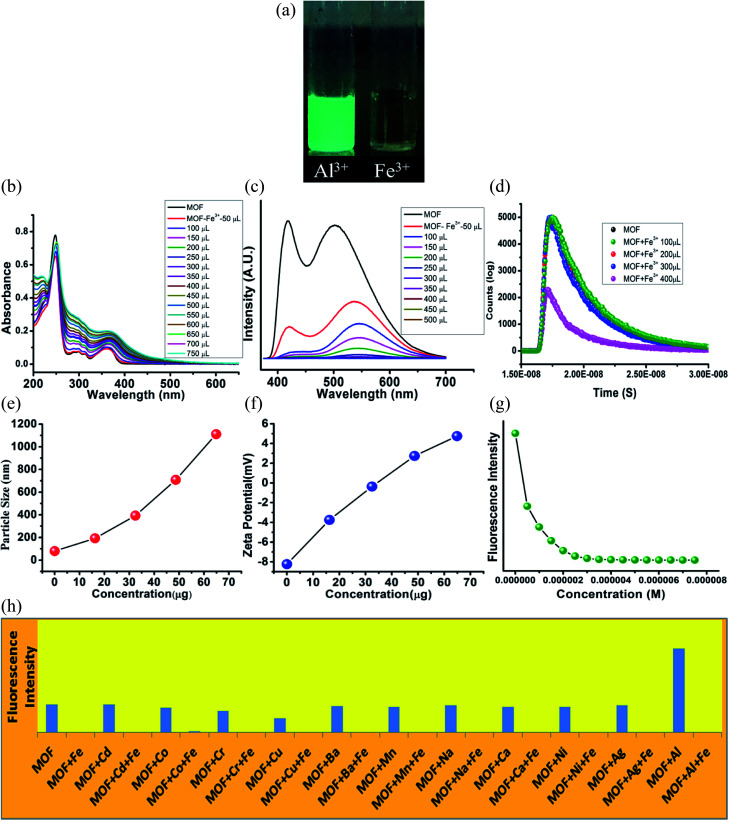
Fluorescence quenching phenomenon of MOF in presence of Fe^3+^ ions. (A) Fluorescence of MOF in presence of different metal ions in 365 nm light; (B) UV-VIS titration of MOF by Fe^3+^ ions; (C) PL titration of MOF by Fe^3+^ ions; (D) fluorescence decay titration of MOF by Fe^3+^ ions. (E) Dynamic light scattering (hydrodynamic diameter size) titration of MOF by Fe^3+^ ions. (F) Zeta potential titration of MOF by Fe^3+^ ions; (G) fluorescence intensity of MOF in presence of Fe^3+^ ions; (H) fluorescence intensity of MOF in presence different metal ions and metal ion-Fe^3+^ solution.

The UV-VIS in presence of Fe^3+^ ions featured some heavy alterations. The Fe^3+^@MOF showed a slight red shifted (around 2 nm) π–π* peak along with a decrease of absorbance. But the discrete π–π* and n-π* peaks are heightened in such a way that the separate discreet peaks merged to become a single peak with broad shoulders. To observe the alteration of the curve nature closely, the MOF was subjected to UV-VIS titration with 10^−3^ M Fe^3+^ solution (Fig. 5B). The titration also showed a gradual heightening of discrete π–π* and n-π* peaks only to form the single wide peak (Fig. 5B) situated in a region of 225–500 nm range. Again equivalent amount of Fe^3+^ ions were added to all the metal ion solutions of MOF to measure the UV-VIS phenomenon (Fig. 21S†). All the solutions exhibited curve natures similar to Fe^3+^@MOF solution (Fig. 21S†). The PL of the MOF in presence of Fe^3+^ solutions displayed complete quenching of fluorescence (Fig. 5C). PL titration with 10^−3^ M Fe^3+^ solution also showed gradual quenching of luminescence along with a slight red shift (Fig. 5C) whereas the *λ*_Em_ shifted upto 546 nm. Again the binding selectivity was obtained by adding equivalent amount of Fe^3+^ in all the metal ion solution of MOF. The PL of all the solutions showed an effective quenching (Fig. 22S†). The corresponding Stern–Volmer curve was found to be linear at lower concentration and nonlinear at higher concentration which suggested the presence of both dynamic and static quenching mechanisms. The Stern–Volmer constant (*K*_sv_) is 7.131 × 10^6^ M^−1^ (Fig. 26S†). The LOD is calculated as 3.3 × 10^−8^ M (33 nM) (Fig. 32S†). The calculated LOD for Fe^3+^ ions are also one of the lowest reported in literature.^50,53–55^

The fluorescence decay measurements displayed a slight reduction in average lifetime (1.26 ns) of the MOF in presence of lower Fe^3+^ concentration where in higher Fe^3+^ concentration the fluorescence intensity reduced rapidly (Fig. 5D). This phenomenon indicated formation of a non-fluorescent complex in ground state. The average hydrodynamic diameter of the MOF in presence of Fe^3+^ increased rapidly. With addition of around 400 μL of 10^−3^ M Fe^3+^ solution the average diameter size increases up to around 1110 nm (Fig. 5E) while the zeta potential increases up to 4.75 mV (Fig. 5F). In this case also the recyclability was observed for 5 cycles. The intensity of the MOF showed a slight decline in fluorescence intensity. Around 10% intensity of MOF reduced in the 5^th^ cycle which could be due to partial degradation of the framework (Fig. 33S†) although the corresponding PXRD of the recovered MOF showcased good stability. Time dependent fluorescence measurements with 300 μL 10^−3^ M Fe^3+^ solution exhibited a 65% of intensity reduction within 8 second time. It further reduces up to 72% within 850 seconds (Fig. 35S†).

### Mechanism

**Fig. 6:**
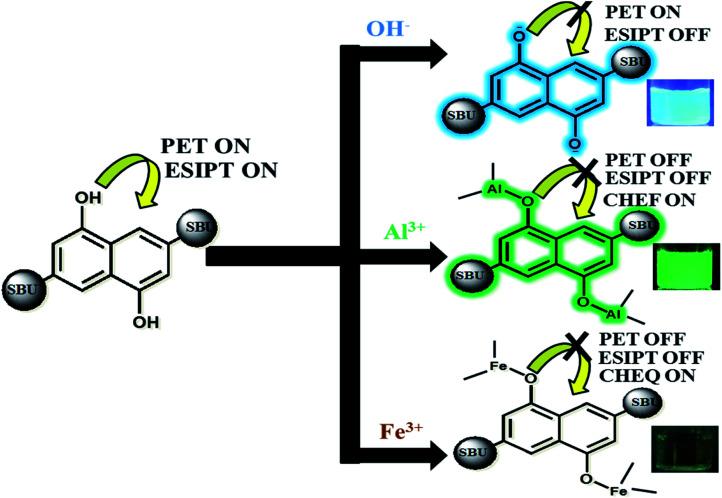
Possible mechanism of MOF-ion interaction triggering CHEF and CHEQ.

Our approach to explore the mechanism of MOF-ion interactions are based on the complexation between MOF and ionic components. These interactions severely affected the ESIPT based optical properties. Fig. 6 represents the proposed binding mechanism between MOF and ions. In neutral water the MOF showed a less intense faint bluish green fluorescence. The lone pair of electron on –OH functional groups is involved in photoinduced electron transfer (PET) process which can reduce the emission intensity quite a bit. It is also noteworthy that ESIPT can also decrease fluorescence intensity.^63^ The UV-VIS titration of MOF with OH^−^ and Al^3+^ ions projected a large bathochromic shift which represent the deprotonation of the –OH functional group.^48,56^ The hydroxyl proton is the key component in keto–enol tautomerism and ESIPT. Therefore for OH^−^ ions the deprotonation lead to suppression of ESIPT which enhances the fluorescence intensity. Al^3+^ ions however first deprotonated and then successfully chelated with the –OH group.

With increasing amount of Al^3+^ the hydroxyl lone pair of electrons got blocked which evidently resulted in suppression of PET and ESIPT simultaneously. Such interaction also activated CHEF^57^ causing the enhancement of fluorescence intensity. The deactivation of ESIPT with increasing concentration of Al^3+^ and OH^−^ ions could also be observed from the PL titrations which displayed a gradual transformation of the two headed peak nature of MOF. For Fe^3+^ ions, the mechanism of binding is similar but the optical phenomenon is quite different due to its paramagnetic quenching nature. The UV-VIS titration with Fe^3+^ showcasing broadening of the plot represented the ligand to metal charge transfer (LMCT).^58^ The LMCT occurs from electron rich orbitals (p) of –OH oxygen to vacant orbitals (s) of Fe^3+^. Due to LMCT a reduction of π electron density can be observed which lead to the drastic reduction of fluorescence intensity. The PXRD collected from the recycled MOF showed good stability of the peaks. The EDX data of thoroughly washed MOF (Fig. 37S†) recovered from the sensing experiments showed no presence of Al^3+^ or Fe^3+^ ions which rules out the possibility of ion exchange. Therefore the fluorescence enhancement or quenching does not occur from any kind of ion exchange or framework degradation. For Fe^3+^, the TCSPC data and Stern–Volmer plot suggested the presence of static quenching mechanism where the MOF forms a non-fluorescent ground state complex with Fe^3+^ ions. The TCSPC data is summarised in Table 1.

**Table 1:** TCSPC of MOF and MOF-ions

Medium	Amp (α1) = B1/(B1 + B2)	Amp (α2) = B2/(B1 + B2)	Lifetime (*τ*1/nS)	Lifetime (*τ*2/nS)	*χ*2	Avg. lifetime *T* (nS)
MOF	0.9256	0.0743	2.528	8.485	1.172	2.971
MOF-OH^−^	0.1008	0.9016	0.108	8.195	1.150	7.381
MOF-Al^3+^	0.3179	0.6821	3.553	9.379	1.146	7.527
MOF-Fe^3+^	0.7076	0.2923	0.459	3.204	1.173	1.261

Such results also indicated the presence of CHEQ mediated quenching behaviour for Fe^3+^ ions.^59^ Metal ions in water reside in a heavily hydrated solvent sphere which can interfere in interactions between –OH and metal ions. But metal ions possessing high ionic potential such as Al^3+^ and Fe^3+^ ions successfully approach and chelate with the –OH groups which also affected the potential of the MOF. Therefore zeta potential of MOF turning positive in presence of Al^3+^ and Fe^3+^ ions also hinted complexation between MOF and metal ions. The MOF is also capable of differentiating between Al^3+^ and Fe^3+^ ions. Selectivity between these two metal ions was measured by performing PL titrations of Al^3+^@MOF with 10^−3^ M Fe^3+^ solution and Fe^3+^@MOF solution with 10^−3^ M Al^3+^ solution.

The titration of Al^3+^@MOF with Fe^3+^ solution showed a rapid decline of fluorescence intensity with the gradual addition of Fe^3+^ ions (Fig. 29S†). But for the titrations of Fe^3+^@MOF with Al^3+^ solution no fluorescence enhancement was observed (Fig. 30S†). Therefore the MOF showed a higher selectivity for Fe^3+^ ions. The MOF in presence of Cu^2+^ also showcased paramagnetic quenching^60^ although compare to Fe^3+^ it is less.

Therefore observations conclude the reason behind turn-on-off optical phenomenon is the acid-base complexation based mechanism between –OH group of the MOF and ionic components.

## Biological studies

**Fig. 7:**
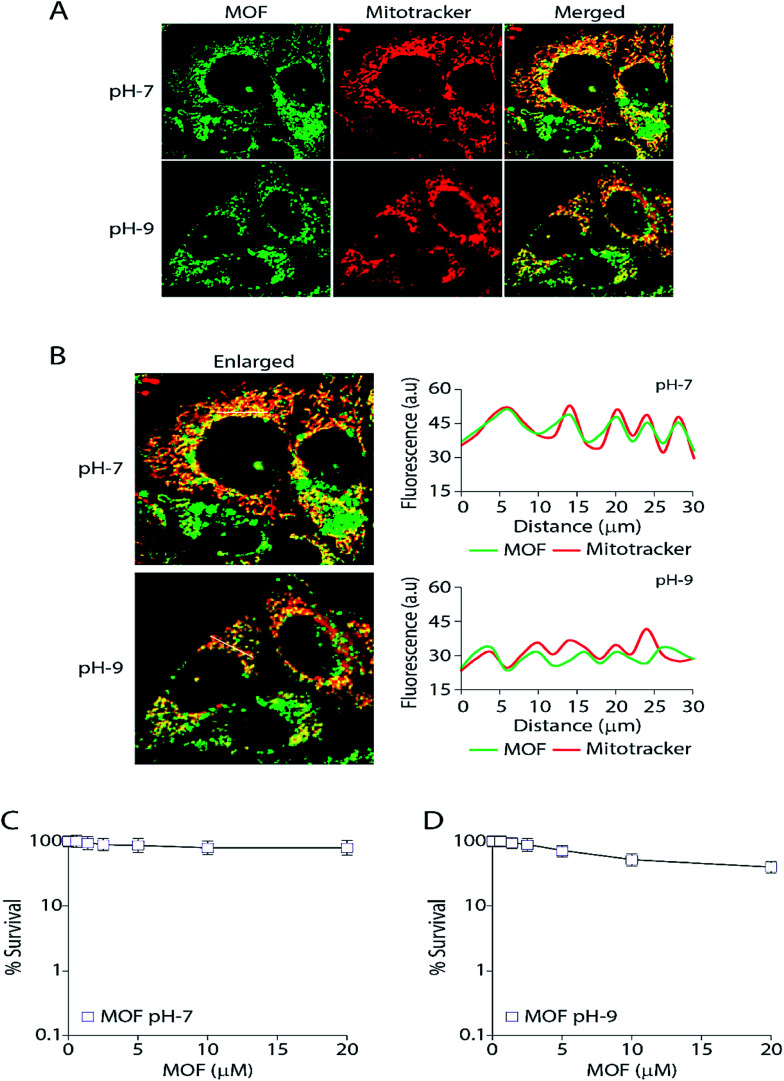
Delivery of –OH functionalized Zn-DHNDC (green) inside mitochondria and partially in cytoplasm shows no cytotoxicity. (A) Representative Live cell confocal images showcasing localization of MOF (10 μM) in MCF7 cells as observed by the green fluorescence of the MOF (green channel). As a mitochondrial marker MitoTracker red (red channel) was used. Right panel exhibits the merged and enlarged images; (B) quantification of the pixel intensities of the MOFs fluorescence and MitoTracker red along the indicated white line in the merged and enlarged image (left panel). The white line was drawn arbitrarily along the region of interest (ROI). a.u., arbitrary units; (C) survival curves (MTT assays) of MCF7 cells treated with MOF at pH-7 for 72 h. The standard deviation (*n* ≥ 3) was represented by the error bars. (D) Survival curves (MTT assays) of MCF7 cells treated with MOF at pH-9 for 72 h. The standard deviation (*n* ≥ 3) was represented by the error bars.

Finally the MOF was subjected for live cell confocal microscopy to explore its potential for the subcellular localization. The MOF in presence of high pH solution and Al^3+^ showed an enhanced turn on fluorescence emission. Rapid aggregation MOF particles shown in Fig. 4E in presence of Al^3+^ render it unsuitable for labelling cancer cells. Therefore all imaging was performed as the MCF7 cells were incorporated separately with MOF in pH-7 (neutral pH) and pH-9 (high pH) buffer solutions for 3 hours. The cells were also costained with the mitochondrion-specific dye MitoTracker red. The particle size of the MOF in pH-7 and pH-9 remained in the range of 80–120 nm. The cell survival assay (Fig. 7C and D) performed to validate cytotoxicity of the MOF revealed no cell killing activity. The fluorescent images certainly exhibit an unaltered nuclear morphology without any significant deformation confirming the bio-compatibility of the MOF inside cellular medium. Upon excitation, the fluorescent particles of MOF were found exclusively inside the mitochondria and partially in the cytoplasm. Surprisingly, the MOF localization completely excluded the nucleus (Fig. 7A). The correlation of the pixel intensities along a line in the two channels (green and red) clearly substantiated the colocalization of the MOF inside mitochondria.

Mitochondria specific labelling mechanisms usually follow two routes. The first route consists of labelling agents utilizing the negative membrane potential (up to −180 mV) of the mitochondrial matrix. In this case lipophilic type cations such as triphenylphosphonium (TPP) were attached with the labelling agent to bestow a cationic character.^61^ Furthermore due to this cationic nature the MOF can easily target the outer membrane of the mitochondria. The second route consists of the labelling agent targeting and binding with the mitochondrial receptors or lipids such as benzodiazepine, cardiolipin *etc.*^62^ Since the MOF at pH 7 or 9 possess negative zeta potential therefore we propose that mitochondria labelling by Zn-DHNDC follow the second route in which the metal ions of the MOF binds with these receptor in pH-7 creating a perfect co-localization between MOF and mitotracker. However at higher pH (pH-9) as the zeta potential of the MOF solution becomes further negative it could affect the degree of co-localization observed in Fig. 7B. Together these data suggest that the MOF is completely non-toxic and retained exclusively inside the mitochondria and partially in the cytoplasm while excluding the nucleus.

## Conclusions

In summary, we developed a novel strategy for ESIPT tuned turn-on-off sensing of OH^−^, Al^3+^ and Fe^3+^ ions and mitochondria targeted bio imaging by hydroxyl functionalized Zn-DHNDC MOF. The exceptional sensing capacity of ZnDHNDC MOF originates from the –OH group interacting with ionic components and trigger processes such as CHEF and CHEQ. The interactions also lead to suppression of ESIPT which especially facilities the turn on fluorescence phenomenon for OH^−^ and Al^3+^ ions. For Fe^3+^ ions, the CHEQ assisted quenching follow the static mechanism where the MOF forms non fluorescent ground state complex with Fe^3+^ ions and trigger LMCT. The selectivity and sensitivity is not only limited to versatile fluorescence phenomenon for each of these ions but also expand to low LOD values (48 nM of OH^−^, 95 nM for Al^3+^, 33 nM for Fe^3+^ ions), fast response time (8 second) and high recyclability of the MOF. Despite several reports on ESIPT based fluorescent MOFs, ESIPT tuned sensing mechanisms of Al^3+^ and OH^−^ ions by MOFs are rarely reported. In fact to the best of our knowledge this is the first report on sensing of hydroxide ion by an ESIPT based MOF.

In cellular medium the MOF showing a selective labelling of important organelles such as mitochondria also establishes its relevance in bio medical field. When subjected to live cell confocal microscopy with MCF7 cells the MOF showed high mitochondria accumulation in pH-7 medium along with a bio-compatible nature. Therefore the Zn-DHNDC MOF can be used as a long term sensor and bio-marker as it successfully demonstrate trace level fluorescent turn-on–off sensing of OH^−^, Al^3+^, Fe^3+^ ions in aqueous medium and mitochondria targeted bio-imaging.

## Conflicts of interest

There is no conflict of interest.

## Supplementary Material

RA-011-D1RA03078G-s001
