# Supplementary material for: Highly selective fluorescent turn-on–off sensing of OH−, Al3+ and Fe3+ ions by tuning ESIPT in metal organic frameworks and mitochondria targeted bio-imaging
Source: RSC Adv. 2021 Aug 16;11(45):27787–800. doi: 10.1039/d1ra03078g (PMC9037803; doi:10.1039/d1ra03078g)
Supplement: RA-011-D1RA03078G-s001 [file RA-011-D1RA03078G-s001.pdf]

Electronic Supplementary Material (ESI) for RSC Advances.
This journal is © The Royal Society of Chemistry 2021

Supporting Information

Highly selective fluorescent turn-on –off sensing of OH^-, $Al^{3+}$and Fe^{3+} ions by tuning ESIPT in metal organic framework and mitochondria targeted bio-imaging

Soumya Bhowal[a]*, Arijit Ghosh [b]*

Table of content

1) Experimental
2) HRMS of the ligand
3) NMR of the ligand
4) Thermo gravimetric analysis of as-synthesis and activated MOF
5) Fourier-transform infrared spectroscopy
6) Transmission electron microscopy
7) Optical properties of the MOF
8) pH titrations of Ligand and MOF
9) NaOH and HCl vs Emission titration
10) UV-VIS Titrations of the MOF by anions
11) Effect on the UV-VIS of the MOF with addition of OH^- in presence of other anions
12) PL Titrations of the MOF by anions
13) Effect on the PL of the MOF with addition of OH^- in presence of other anions
14) Time-correlated Single Photon Counting of MOF in presence of OH^- anion
15) UV-VIS Titrations of MOF with Metal Ions
16) PL Titrations of MOF with Metal Ions
17) Effect on the UV-VIS of the MOF with addition of Al^{3+} in presence of other Metal ions
18) Effect on the PL of the MOF with addition of Al^{3+} in presence of other Metal ions
19) Time-correlated Single Photon Counting of MOF in presence of Al^{3+} ion
20) Dynamic light scattering (particle size) of the MOF in presence Al^{3+} ions
21) Zeta potential of the MOF in presence of Al^{3+} ions
22) Effect on the UV-VIS of the MOF with addition of Fe^{3+} in presence of other Metal ions
23) Effect on the PL of the MOF with addition of Fe^{3+} in presence of other Metal ions
24) Dynamic light scattering (particle size) of the MOF in presence Fe^{3+} ion
25) Zeta potential of the MOF in presence of Fe^{3+} ions MOF
26) Stability of MOF in different solutions
27) The Stern-Volmer plot of the MOF calculated from titrations with Al^{3+} and Fe^{3+}

28) **UV-VIS titration of Ligand with OH^-**
29) **PL titration of Ligand with OH^-**
30) **Titration of the Al^{3+}@MOF with Fe^{3+} ion solution**
31) **Titration of the Fe^{3+}@MOF with Al^{3+} ion solution**
32) **Fluorescence Intensity of the MOF in presence of Al^{3+} and calculation of LOD**
33) **Fluorescence Intensity of the MOF in presence of Fe^{3+} and calculation of LOD**
34) **Fluorescence Intensity of the MOF in presence of OH^- and calculation of LOD**
35) **Recyclability of the MOF for Al^{3+}, Fe^{3+}, OH^- ions**
36) **Time dependent photoluminescence**
37) **Zeta potential of MOF in presence of pH solutions and OH^- ions**
38) **EDX of MOF recovered from metal ion solution of MOF.**

1. Experimental

Ligand and MOF synthesis

Synthesis of ligand

The synthesis of the 4, 8-dihydroxynaphthalene-2, 6-dicarboxylic acid was obtained in two steps. The first step involved the synthesis of disulpho derivative with sulphonation and the second step involves solid state hydroxylation.

Synthesis of 4, 8-disulfonaphthalene-2, 6-dicarboxylic acid

In a 100 mL round bottom flask 1g of naphthalene-2, 6-dicarboxylic acid was taken and 5 mL of Oleum (SO_3, 65 wt%) was added to it. The solution is stirred at around 140°C for 24 hours. The resulting solution was then cooled to room temperature and added to 50 mL of cold water in a drop wise manner. Acidity of the solution was balanced with Na_2CO_3 solution. A white precipitate formed which was then filtered and washed with water to remove excess acid from the surface of the precipitate. Thus 4, 8-disulfonaphthalene-2, 6-dicarboxylic acid is synthesized. FT-IR (KBr, cm^{-1}): 3596 (br), 2991 (br), 1693 (s), 1623 (w), 1604 (m), 1485 (w), 1427 (s), 1290 (s), 1193 (br), 1130 (br), 1047 (s), 908 (m), 803 (ss), 771 (ss), 738 (ss), 638 (s), 615 (s), 526 (w), 424 (w). 1H-NMR (500 MHz, D_6-DMSO): δ 8.48 (s, 2H), δ 9.604 (s, 2H), δ 13.126 (w, 2H).

Synthesis of 4, 8-dihydroxynaphthalene-2, 6-dicarboxylic acid

0.5 g of 4, 8-disulfonaphthalene-2, 6-dicarboxylic acid and 0.25 g NaOH was mixed, dried and then sealed in a vacuumed glass tube for heating. The sealed tube then placed inside a furnace and heated around 320^{0}C for 2h with a heating rate 6^{0}C/min. After the heating was completed the tube was then brought into room temperature and cut open. The mixture was then dissolved in water and neutralised with diluted HCl solutions. A light yellow colour precipitate formed which was then washed, dried and collected. FT-IR (KBr, cm^{-1}): 3586 (br), 3479 (br), 3135 (br), 2917 (w), 1729 (s), 1610 (w), 1416 (w), 1266 (w), 1178 (ss), 1053 (s), 916 (w), 751 (w), 734 (w), 691 (w), 622 (m). 1H-NMR (500 MHz, D_6-DMSO): δ 7.414 (s, 2H), δ 8.296 (s, 2H), δ 10.690 (s, 2H).

Synthesis of Zn-4, 8-dihydroxynaphthalene-2, 6-dicarboxylate MOF

In a glass based scintillation vial $Zn(NO_3)_2.6H_2O$ (0.03 g, 0.1 mM) and 4, 8-dihydroxynaphthalene-2, 6-dicarboxylic acid (0.025 g, 0.1 mM) were taken. The reactants were dissolved in 3 mL DMF and 2 mL ethanol solvent mixture. The mixture is then dissolved with sonication and sealed tightly with teflon tape. Then the mixture is heated at 80^0C for 12 hours in an oven. An orange colour cubic crystal generated at the bottom of the glass. The crystals were filtered and washed with DMF. FT-IR (KBr, cm^{-1}): 3467 (br), 3104 (br), 2804 (br), 1616 (s), 1585 (m), 1404 (s), 1353 (m), 1191 (ss), 1053 (s), 791 (s), 772 (m), 628 (s).

Activation

The activation method consisted of a two-step procedure to vacate the pores of the MOF from solvents and moisture. First the MOF was treated with dichloromethane (DCM) to replace the trapped high temperature boiling DMF and ethanol with a low temperature boiling DCM solvents. The MOF was then filtered from the DCM solvent and collected. The MOF was then heated and vacuumed at 80^0C for 24 hours. The resulting activated MOF was used for further experiments.

Material

All the starting materials were of reagent grade and used as received from the commercial suppliers without any further purification. Powder XRD (PXRD) of the as-synthesised and activated MOF and MOF in different solutions were collected in Bruker D8 ADVANCE using Cu-Kα (λ = 1.5406 Å) in a 2θ range 5-50^0C. FTIR data of the MOF and ligand was obtained with PerkinElmer Spectrochem 100 FTIR spectrometer. High-resolution mass spectrometry (HRMS) of the ligands was collected on QTOF−Micro YA 263 mass spectrometer in negative ESI mode. Fluorescence emission studies were performed using a HORIBA JOBIN YVON Fluoromax-4 spectrofluorometer. UV-Visible spectra data were collected in Perkin-Elmer Lambda 950 UV/vis instrument. Thermo gravimetric analyses of the As-synthesized and activated MOF were collected with TA Instrument SDT Q600 at a 5 °C/min heating and 100 mL/min flow rate under N_2 atmosphere. TCSPC measurements were obtained in an IBH Fluorocube by exciting with Picoseconds diode laser (IBH Nanoled-07). JEOL JEM 2010 high-resolution microscope instrument is used for TEM images. Dynamic

light scattering (DLS) and Zeta potential of the solutions were obtained using NanoZS90 (Malvern) instrument using Millipore water as solvents.

Live-cell confocal microscopy

Live-cell imaging was carried out using confocal laser-scanning microscope (Leica TCS SP8) with a UV-laser and 63X/1.4 NA oil objective equipped with a heated environmental chamber set to 37º C with optimal CO_2 facility. Briefly, MCF7 cells seeded on cover glass bottom dish (Genetix, Biotech Asia Pvt. Ltd.) were incubated with the MOF at pH-7 and pH-9 separately for 3 hours before imaging. Nuclei were subsequently stained with Hoechst 33342 (Blue) (Invitrogen). This was followed by live cell confocal microscopy. Images were collected and processed using the Leica software and sized in Adobe Photoshop 7.0.

Cell Survival Assay

MCF7 cells (6×10^3) were seeded in 96-well plates (BD Biosciences, USA) and treated with the MOF at pH-7 and pH-9 separately at the indicated concentrations. After 72-h treatment, cell survival was assessed by 3-(4, 5-dimethylthiazol-2-yl)-2, 5- diphenyltetrazolium bromide (MTT) assay. Briefly, cells were washed with 1X PBS and treated with MTT reagent (Sigma) for 3 h at 37º C and the resulting formazan was dissolved in 100 µL of Dimethyl Sulfoxide. Plates were analyzed on Molecular Devices SpectraMax M2 Microplate Reader at 570 nm. The percent inhibition of viability for each concentration of the compounds was calculated with respect to the control. Data represent mean values ± s.e.m. for three independent experiments.

2. HRMS of the ligand

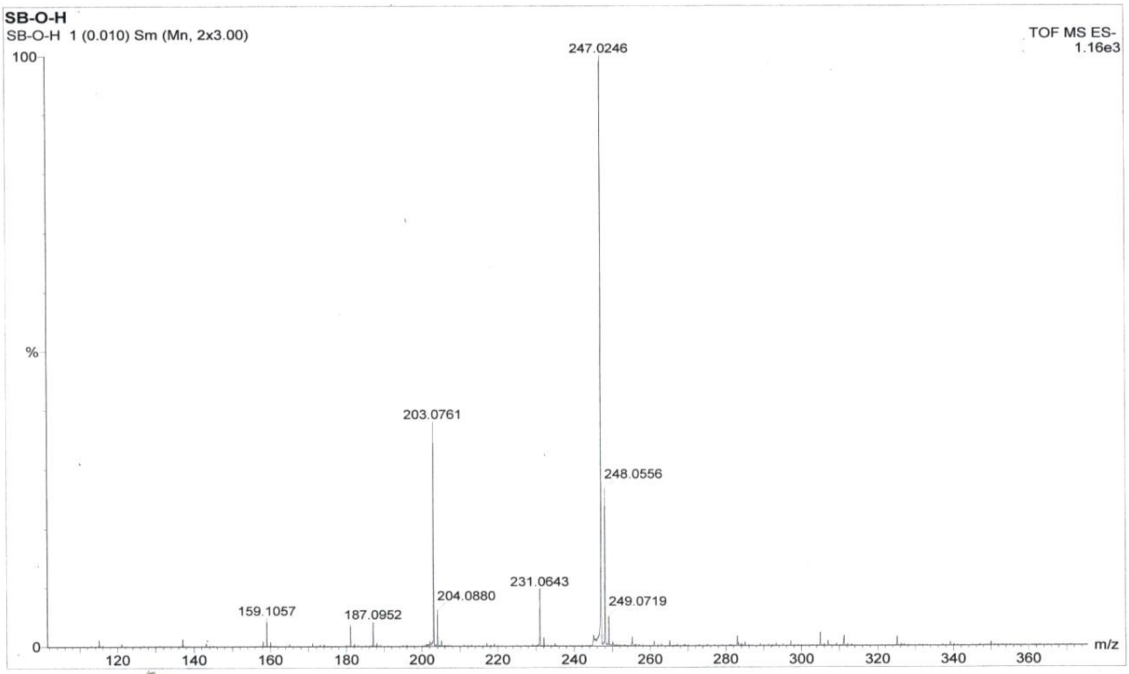

Figure 1S: The HRMS data of 4, 8-dihydroxynaphthalene-2, 6-dicarboxylic acid in ESI (-ve) mode.

3. NMR of the ligand

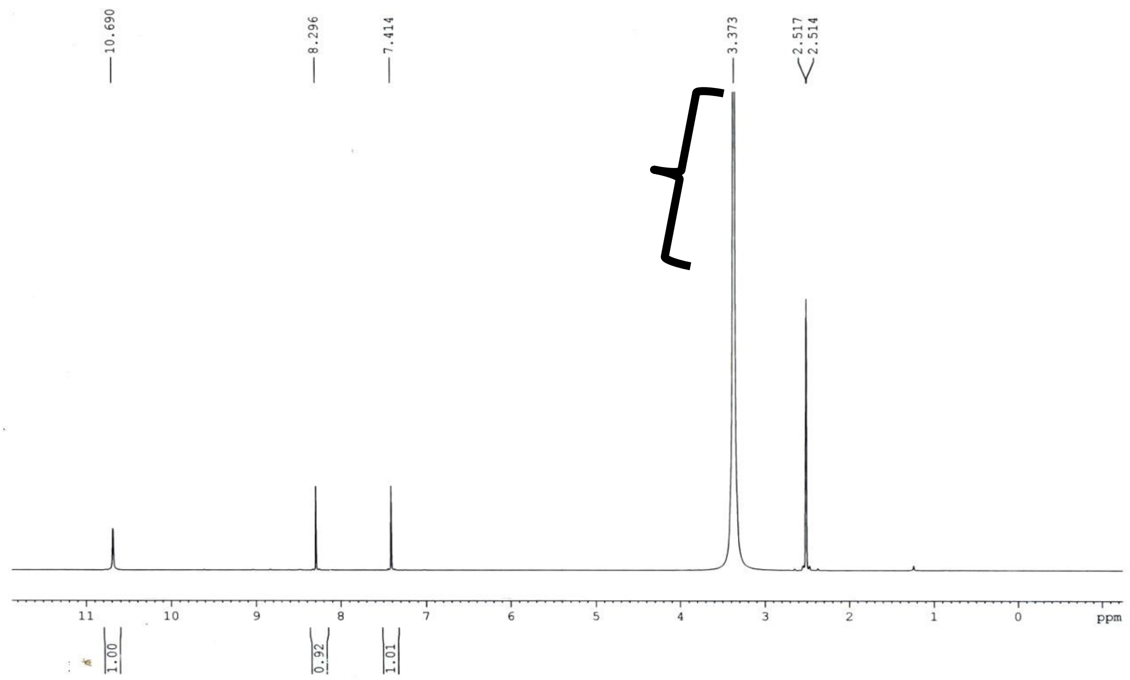

Figure 2S: The ^{1}H NMR of 4, 8-dihydroxynaphthalene-2, 6-dicarboxylic acid

4. Thermo gravimetric Analysis

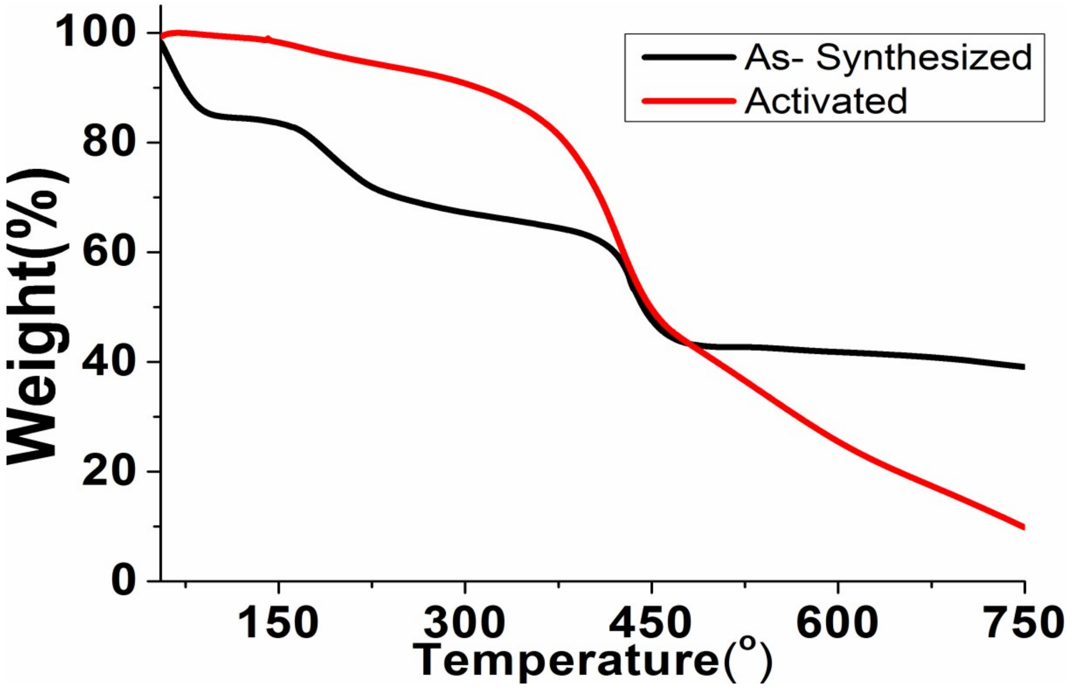

Figure 3S: Thermo gravimetric Analysis of as-synthesised and activated MOF

5. Fourier-transform infrared spectroscopy

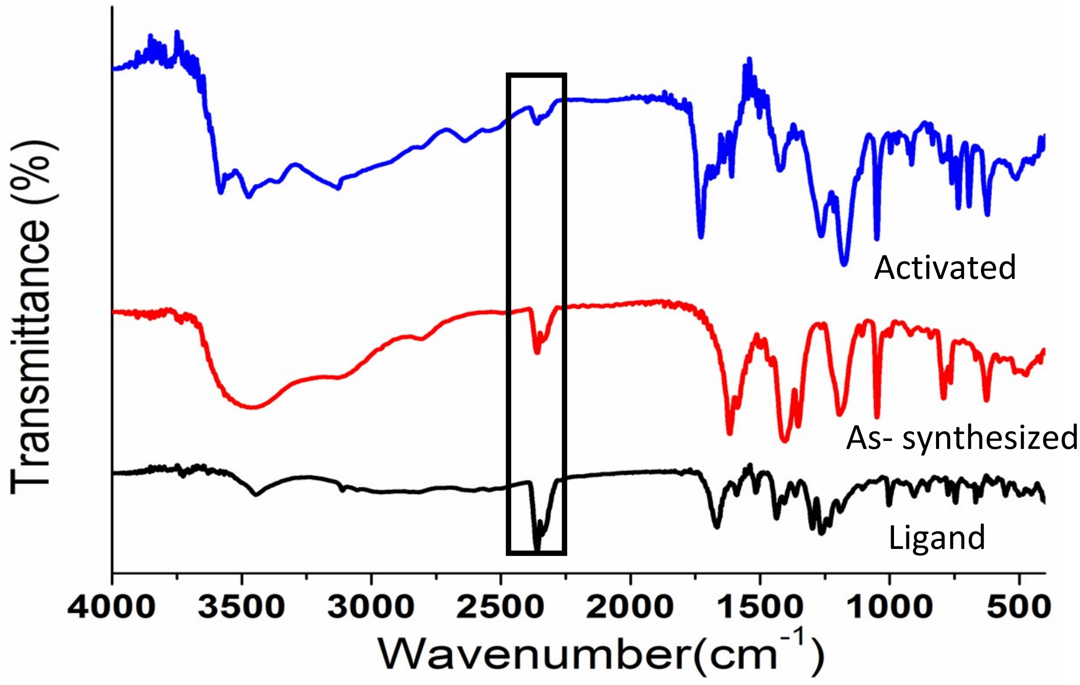

Figure 4S: The FTIR of Activated MOF, as-synthesized MOF and ligand. The black marked peak represents the C-O stretching of atmospheric CO_2.

6. Transmission Electron Microscopy

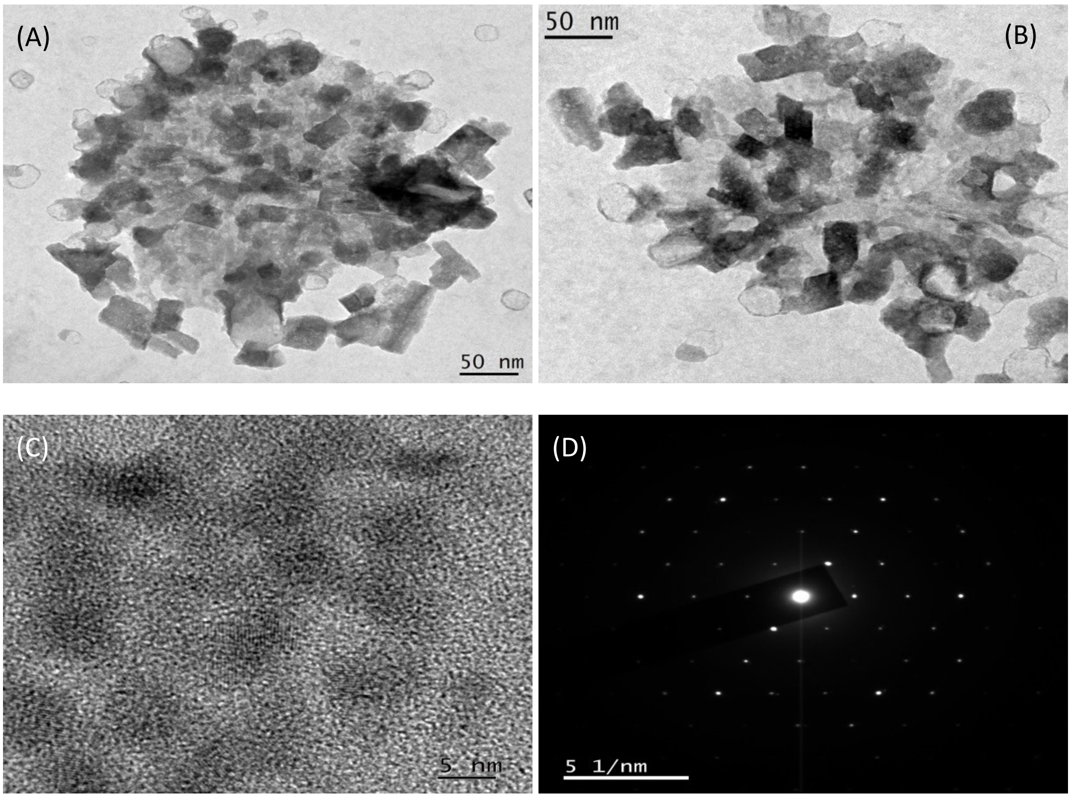

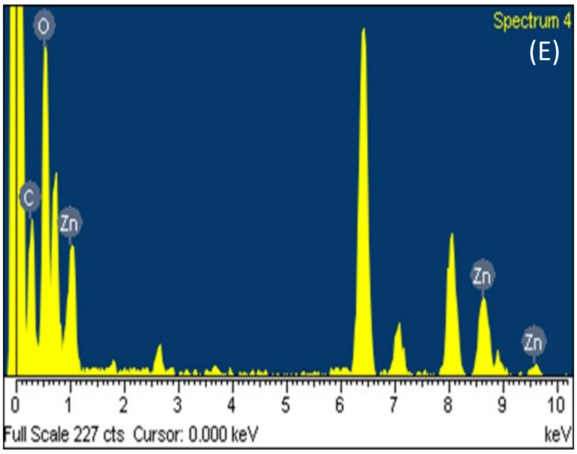

(F)

Element	Weight%	Atomic%
C K	15.12	30.38
O K	33.63	50.71
Zn K	51.25	18.91
Totals	100.00	

Figure 5S: Transmission electron microscopy of the MOF. (A), (B) represents the nano scale MOF particle. (C) Represents the HRTEM image. (D) Represents the SAED of the particles. (E), (F) Represents the EDX data and element analysis table.

7. Optical properties of the MOF

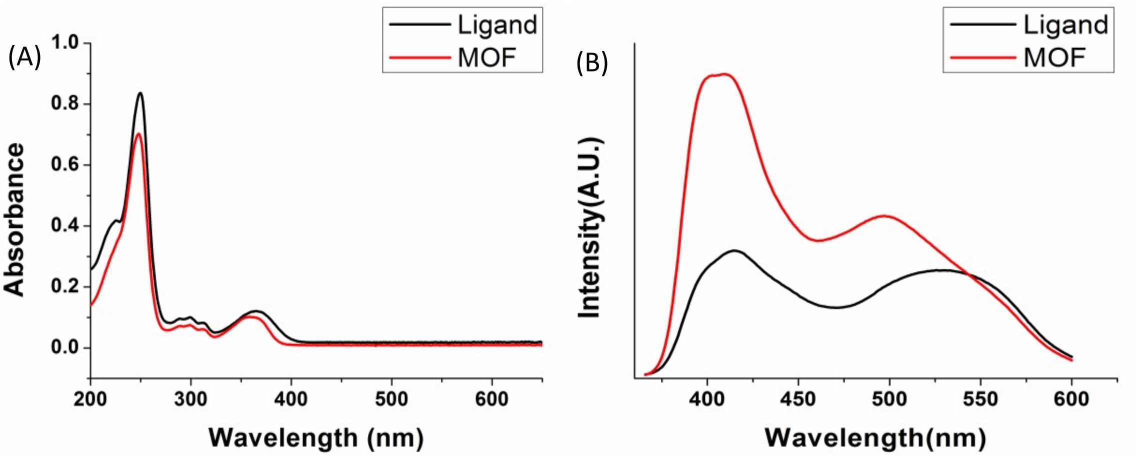

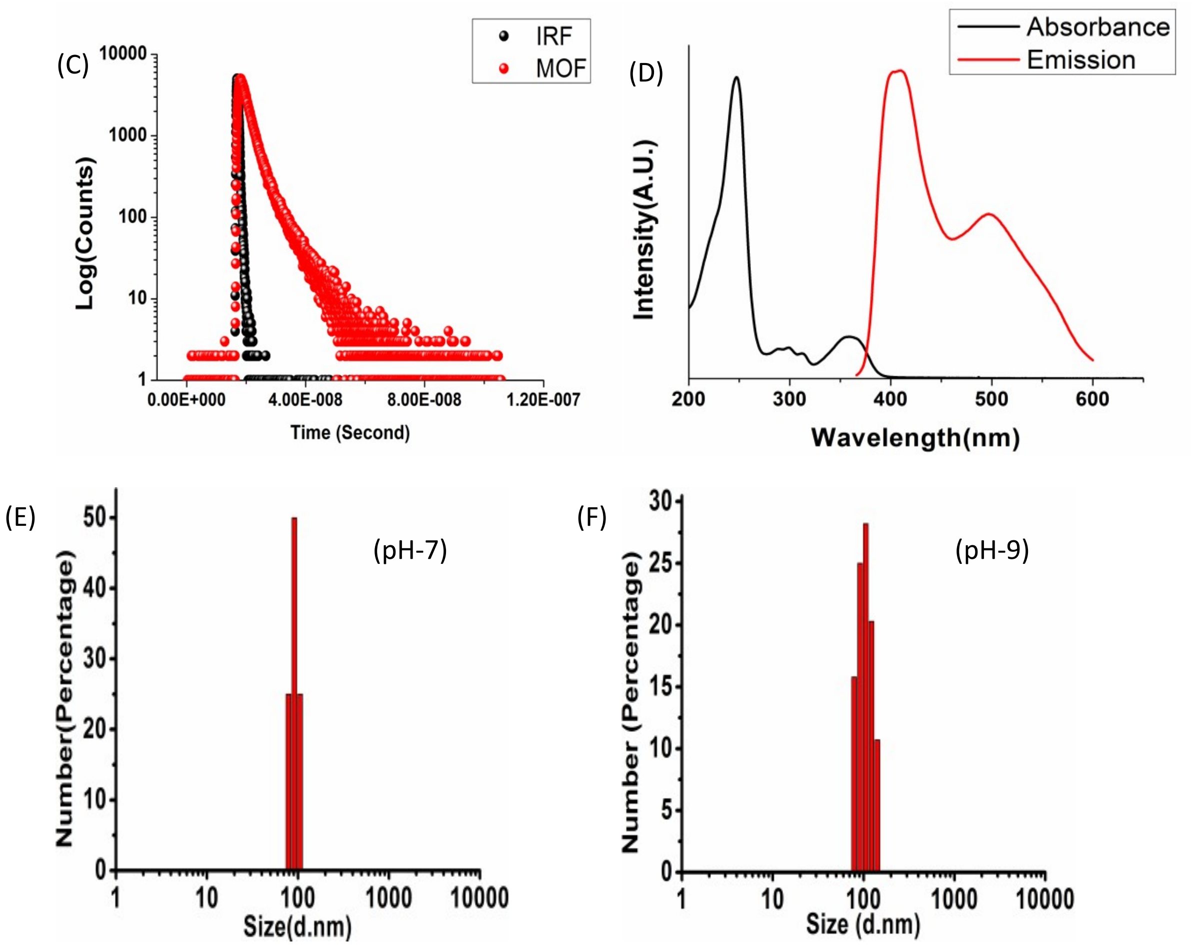

Figure 6S: represents the optical properties. (A) Represents the UV-VIS plots of ligand and MOF. (B) PL data of ligand and MOF. (C) Fluorescence decay (TCSPC) of the MOF. (D) Represents the UV-VIS and PL curve with a huge stocks shift. (E) DLS (hydrodynamic diameter) of the MOF in pH-7. (F) DLS (hydrodynamic diameter) of the MOF in pH-9.

8. pH titrations of Ligand and MOF

(A)

(B)

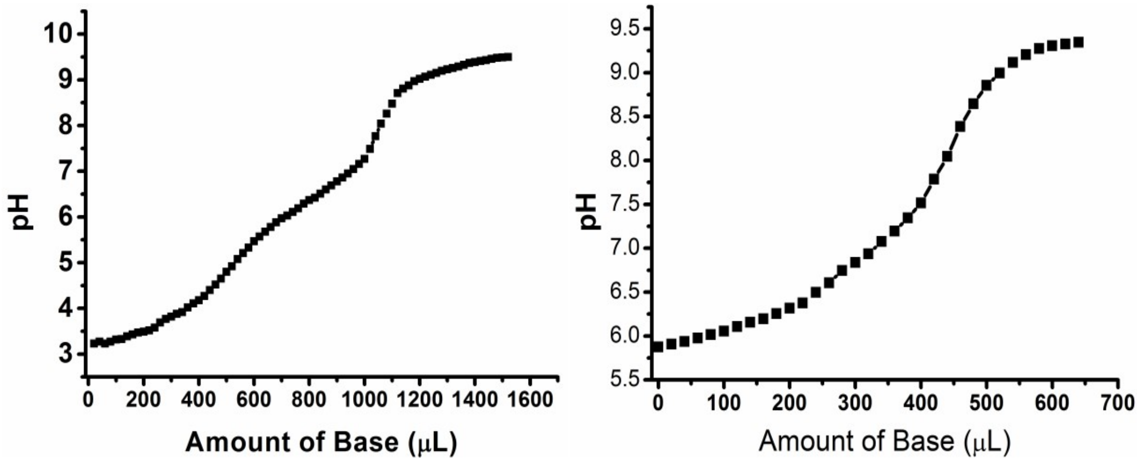

Figure 7S: Base vs pH titration of ligand and MOF. (A) The titration of ligand. (B) The titration of MOF.

9. NaOH and HCl vs Emission titration

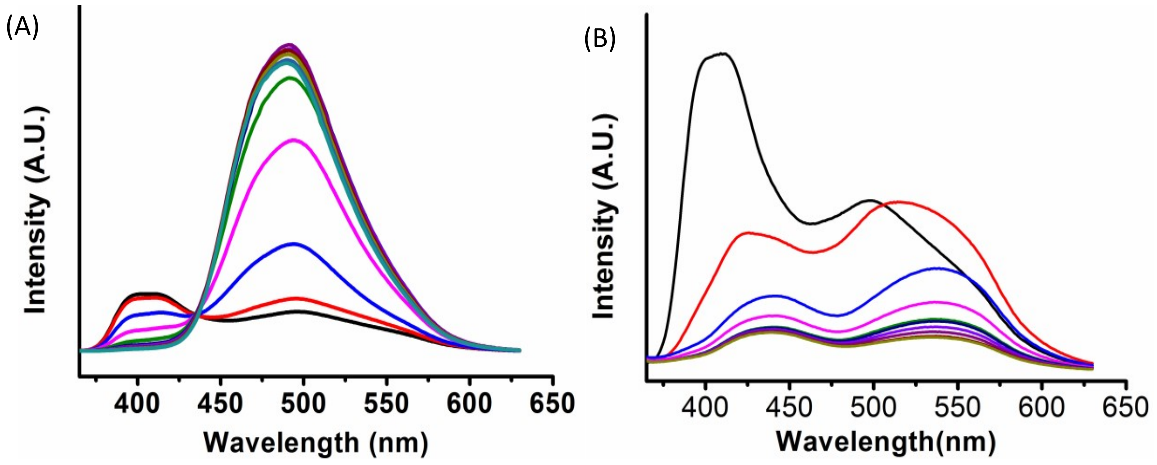

Figure 8S: Titration of MOF by acid and base.(A) Represents the PL titration of MOF with NaOH, (B) PL titration of MOF with HCl.

10. UV-VIS Titrations of the MOF by anions

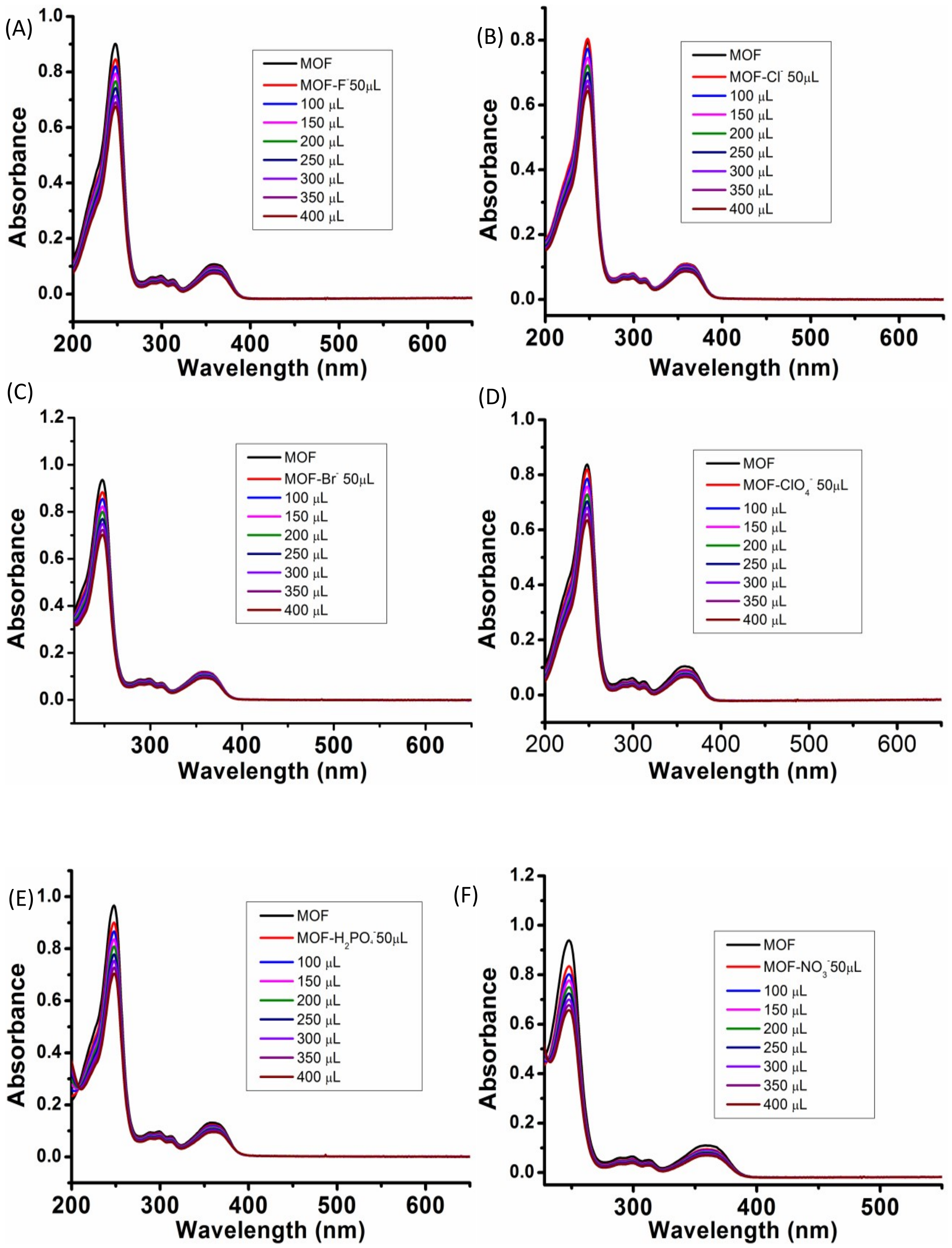

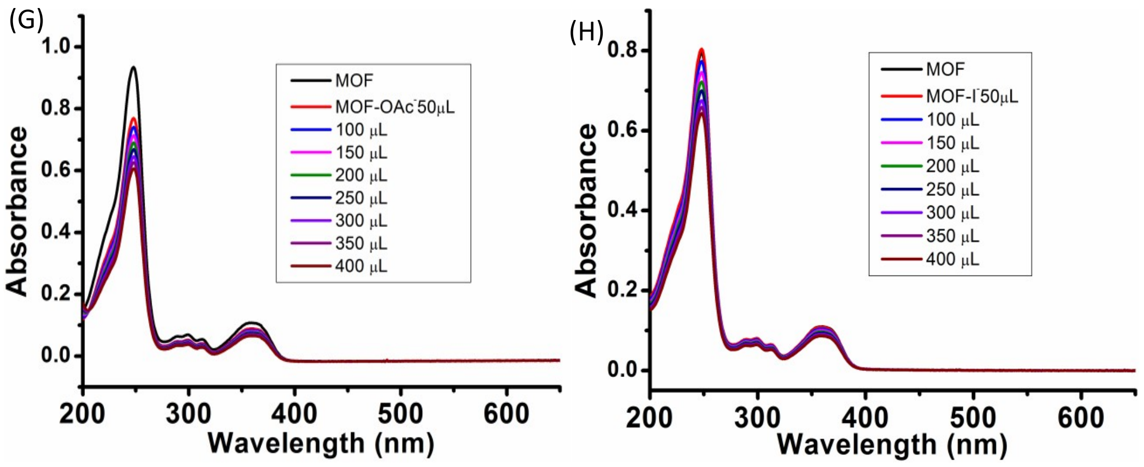

Figure 9S: The UV-VIS titration of the MOFs with (A) F^-, (B) Cl^-, (C) Br^-, (D) ClO_4^-, (E) $H_2PO_4^-$, (F) NO_3^-, (G) OAc^-,(H) I^-.

11. Effect on the UV-VIS of the MOF with addition of OH^- in presence of other anions

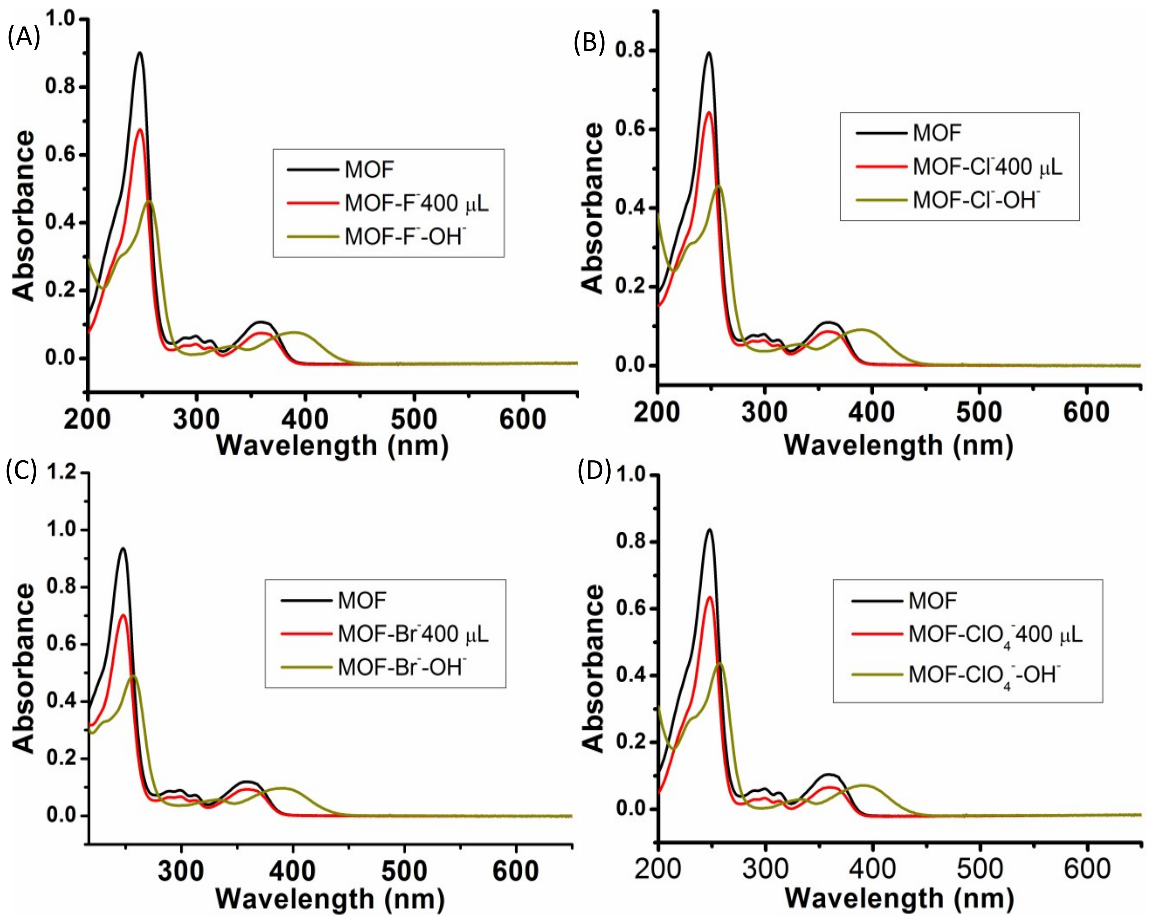

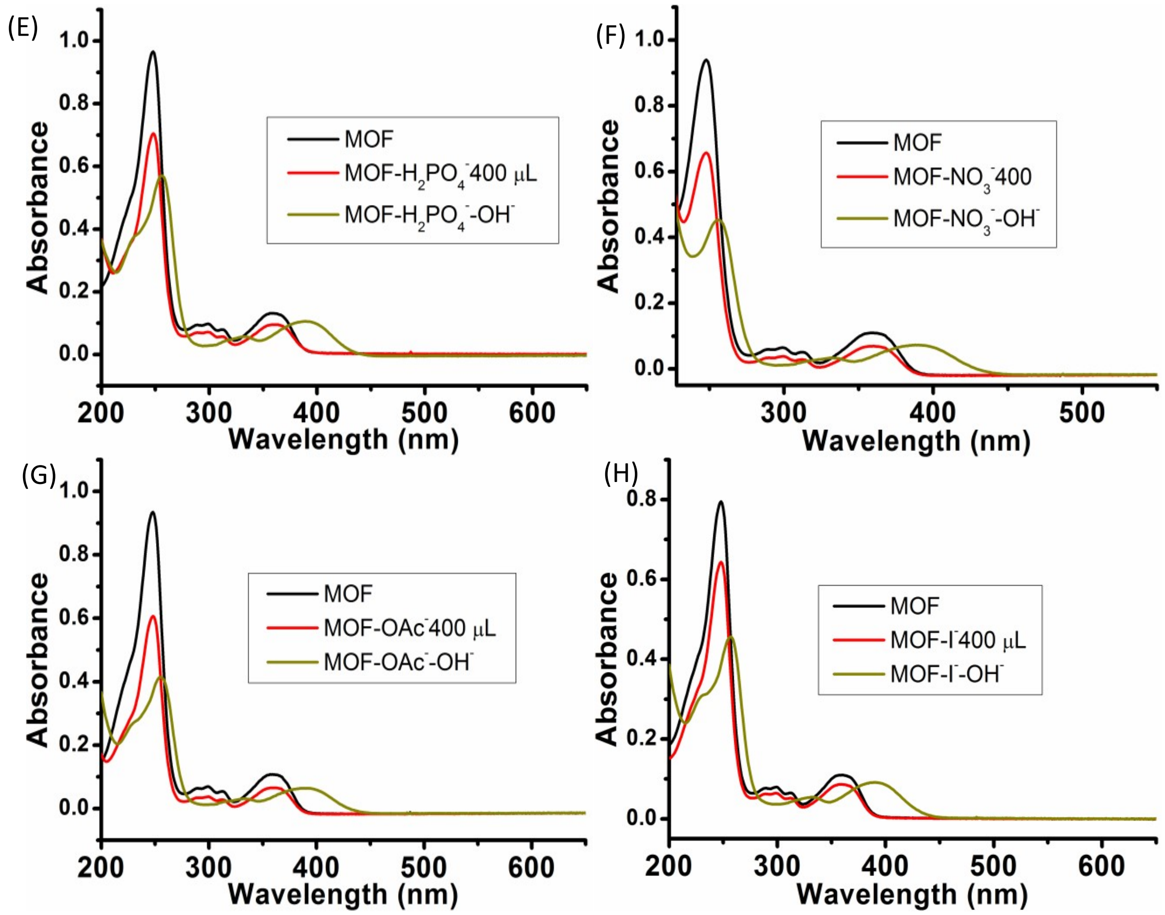

Figure 10S: Effect on UV-VIS curve nature of the MOF due to OH^- addition in the MOF solution with other anions (A) F^-, (B) Cl^-, (C) Br^-, (D) ClO_4^-, (E) $H_2PO_4^-$, (F) NO_3^-, (G) OAc^- ,(H) I^-.

12. PL Titrations of the MOF by anions

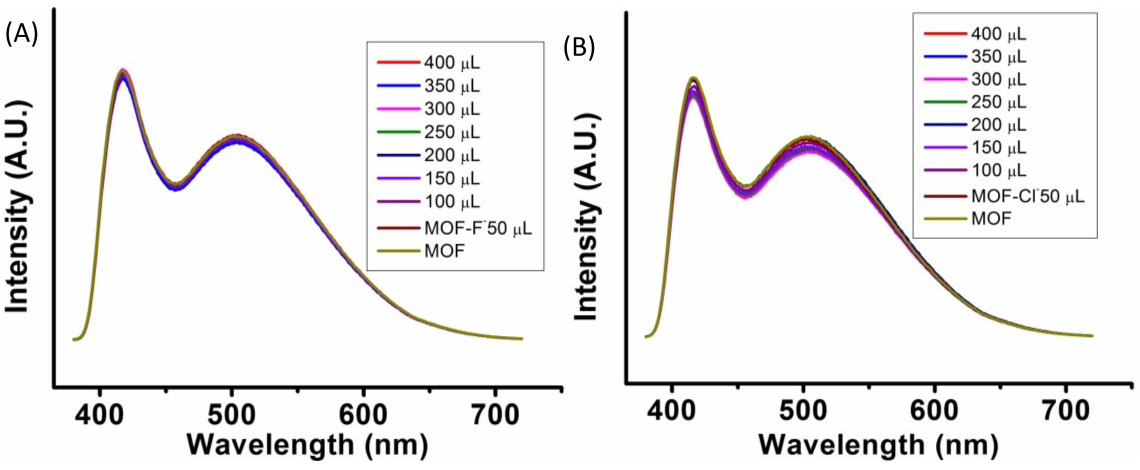

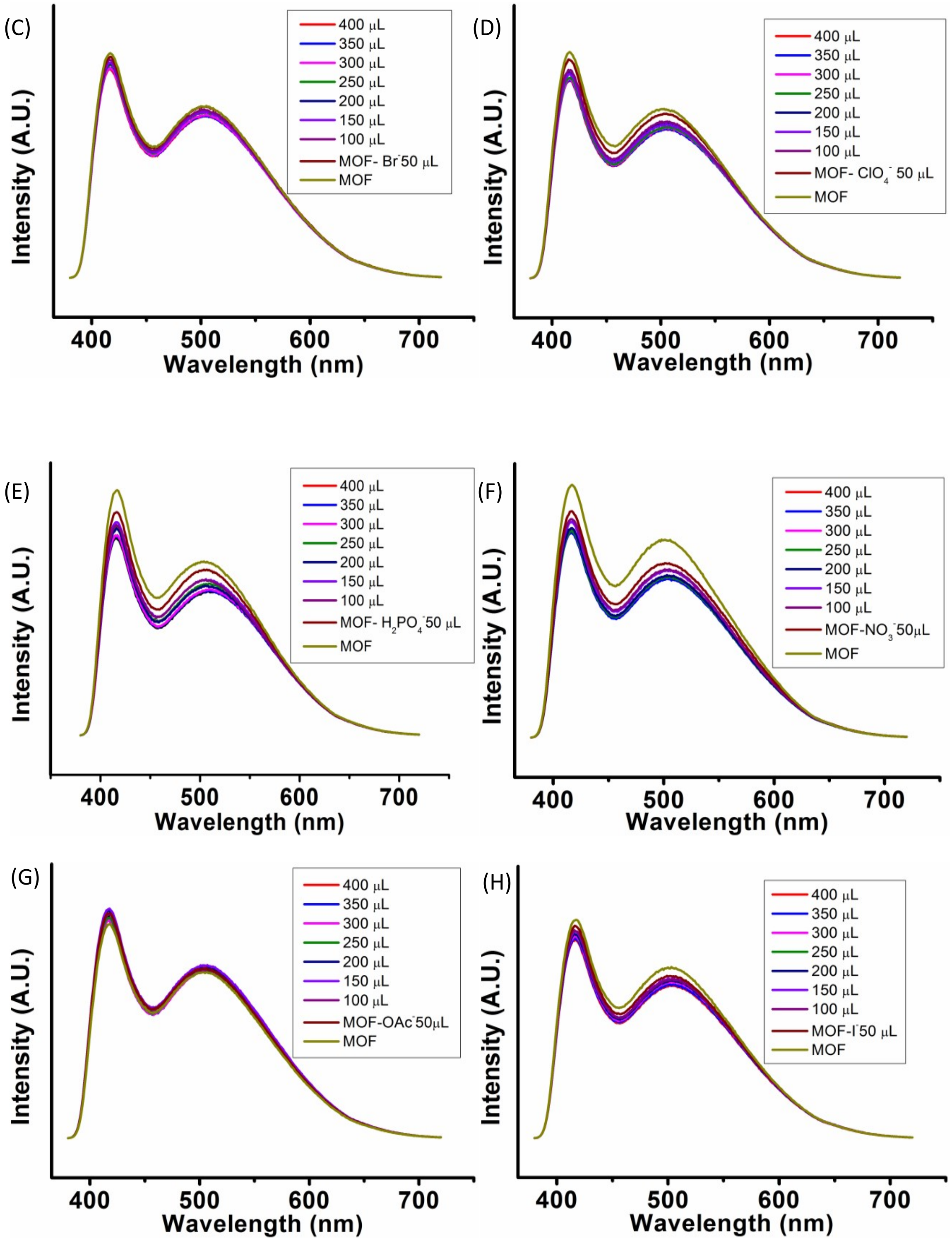

Figure 11S: The PL titration of the MOFs with (A) F^-, (B) Cl^-, (C) Br^-, (D) ClO_4^-, (E) $H_2PO_4^-$, (F) NO_3^-, (G) OAc^-,(H) I^-.

13. Effect on the PL of the MOF with addition of OH^- in presence of other anions

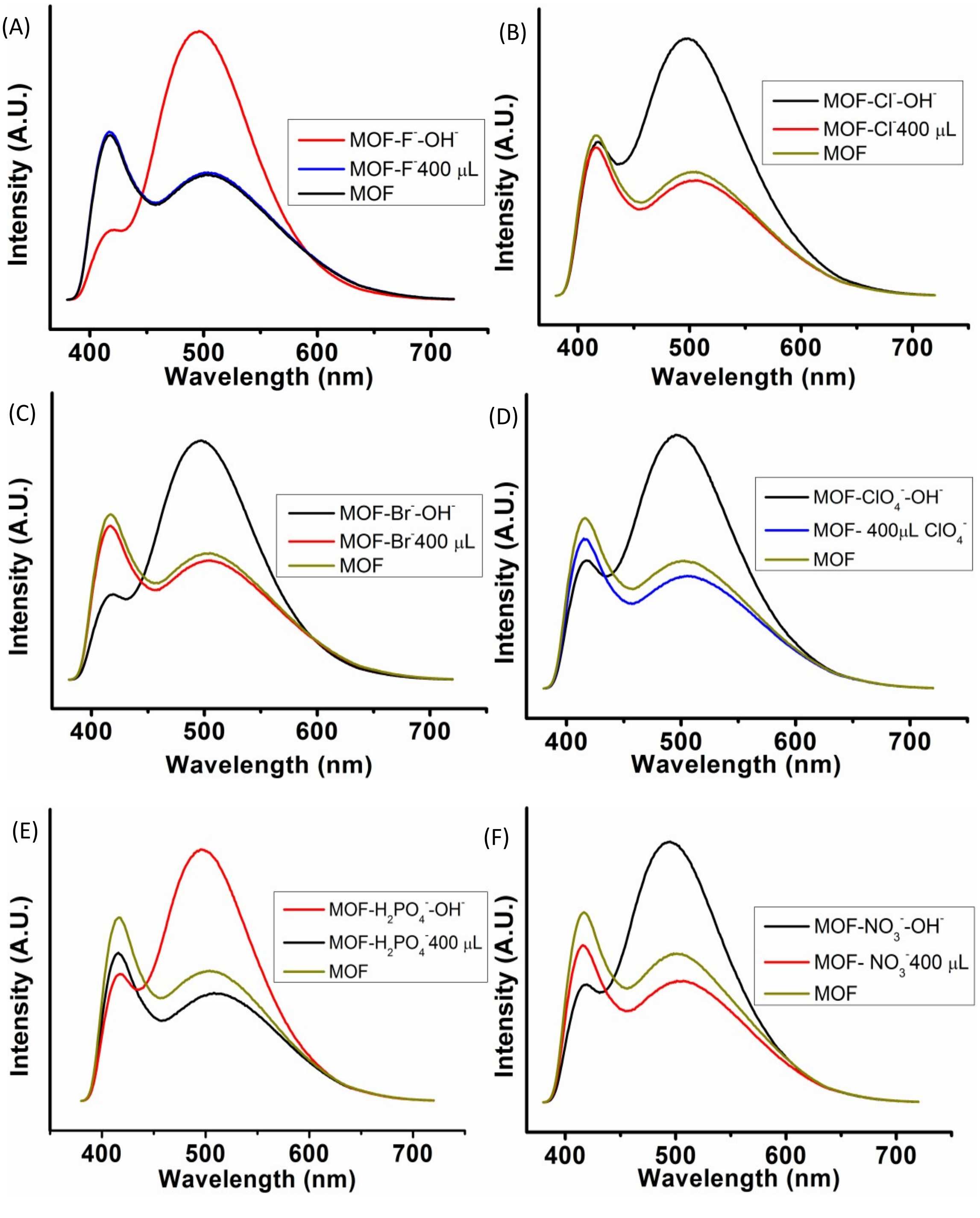

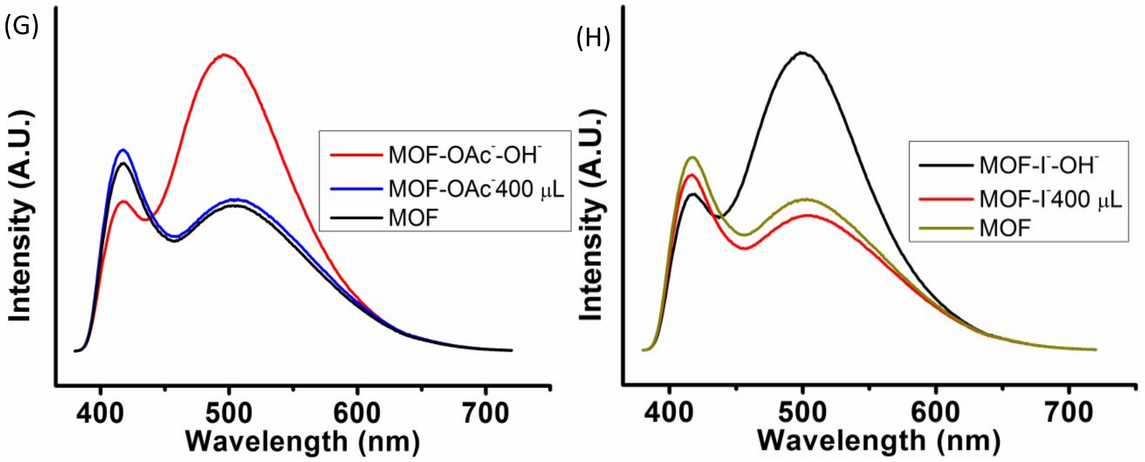

Figure 12S: Effect on PL curve nature of the MOF due to OH^- addition in the MOF solution with other anions (A) F^-, (B) Cl^-, (C) Br^-, (D) ClO_4^-, (E) $H_2PO_4^-$, (F) NO_3^-, (G) OAc^-,(H) I^-.

14. Time-correlated Single Photon Counting of MOF in presence of OH^- anion

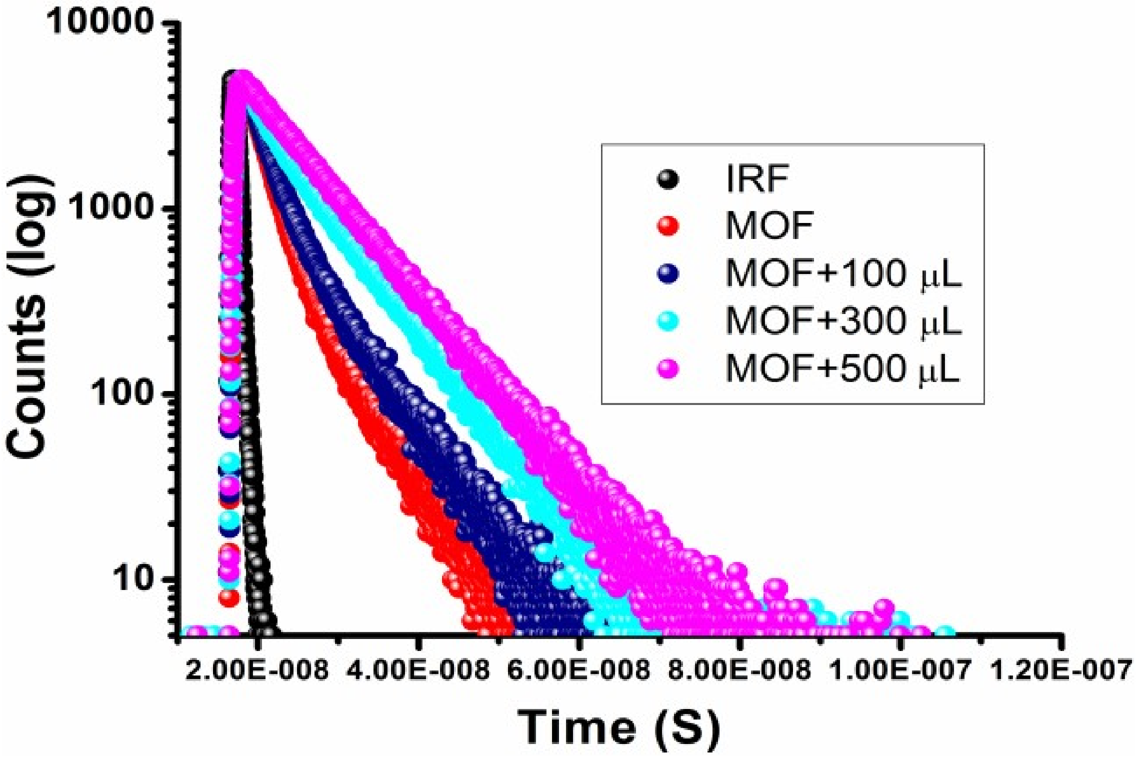

Figure 13S: The fluorescence decay of MOF and MOF solution with OH^- anion

15. UV-VIS Titrations of MOF with Metal Ions

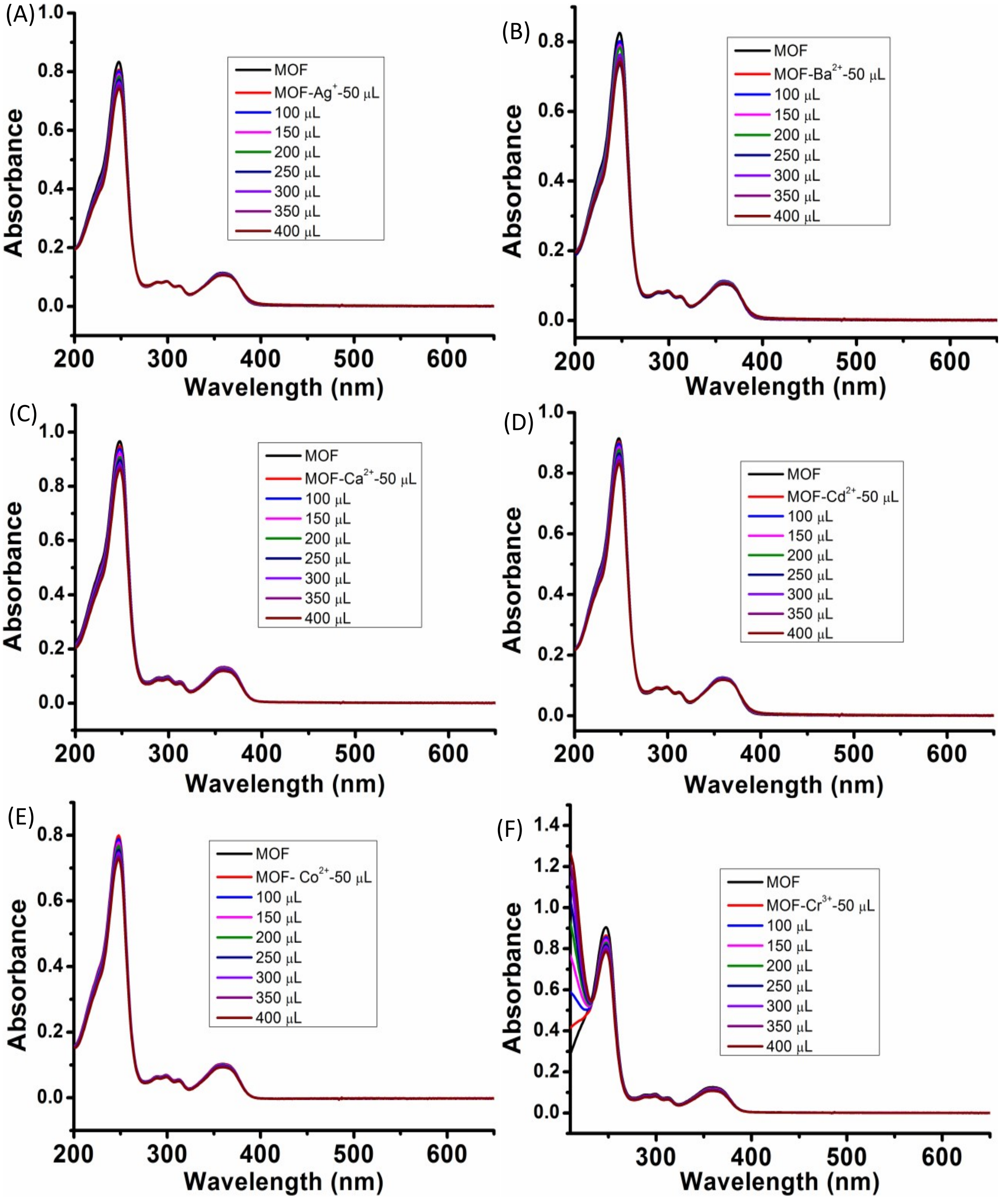

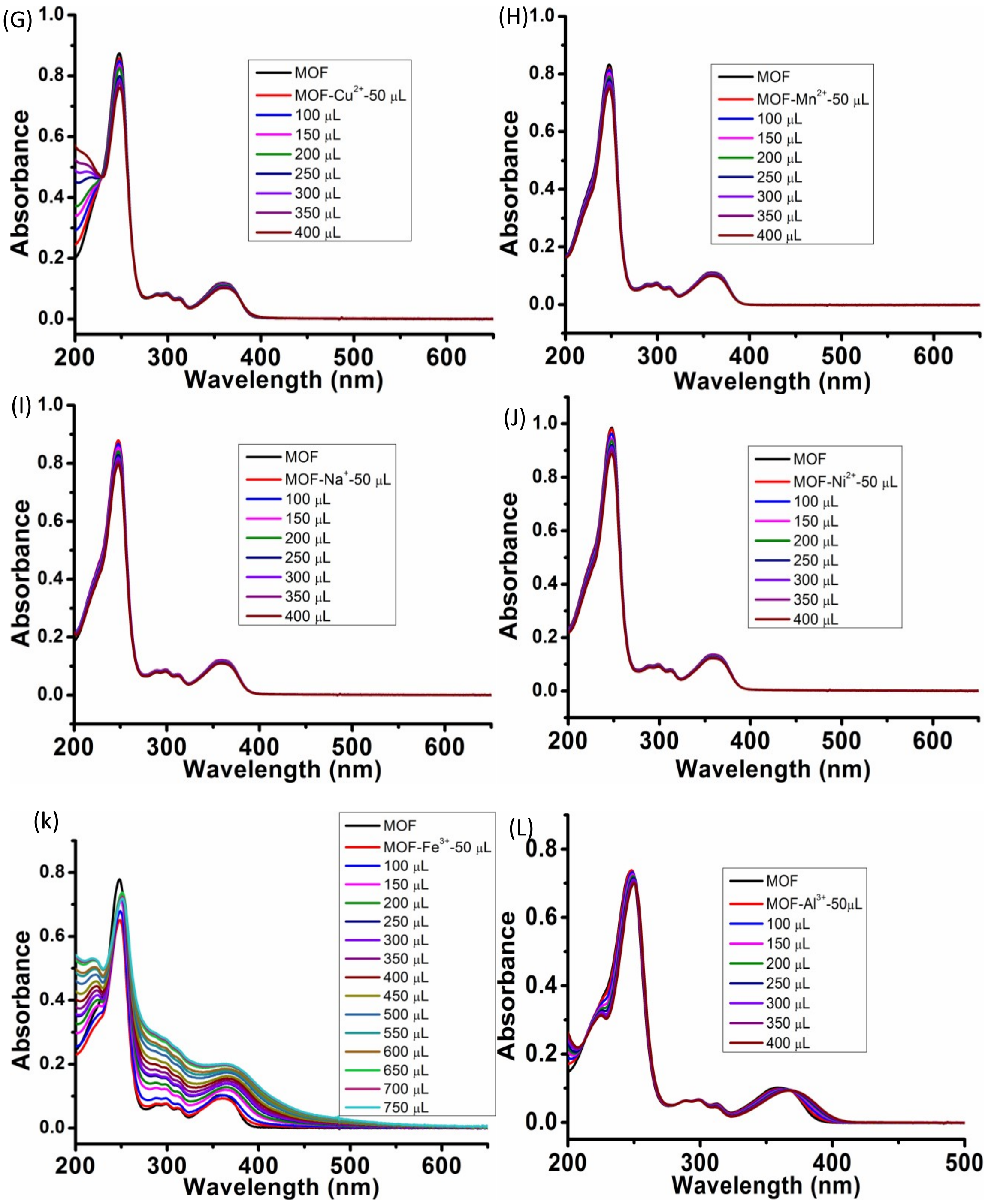

Figure 14S: UV-VIS titration of MOF in presence of metal ions (A) Ag^{+}, (B) Ba^{2+}, (C) Ca^{2+}, (D) Cd^{2+}, (E) Co^{2+}, (F) Cr^{3+}, (G) Cu^{2+}, (H) Mn^{2+}, (I) Na^{+}, (J) Ni^{2+}, (k) Fe^{3+}, (l) Al^{3+}

16. PL Titrations of MOF with Metal Ions

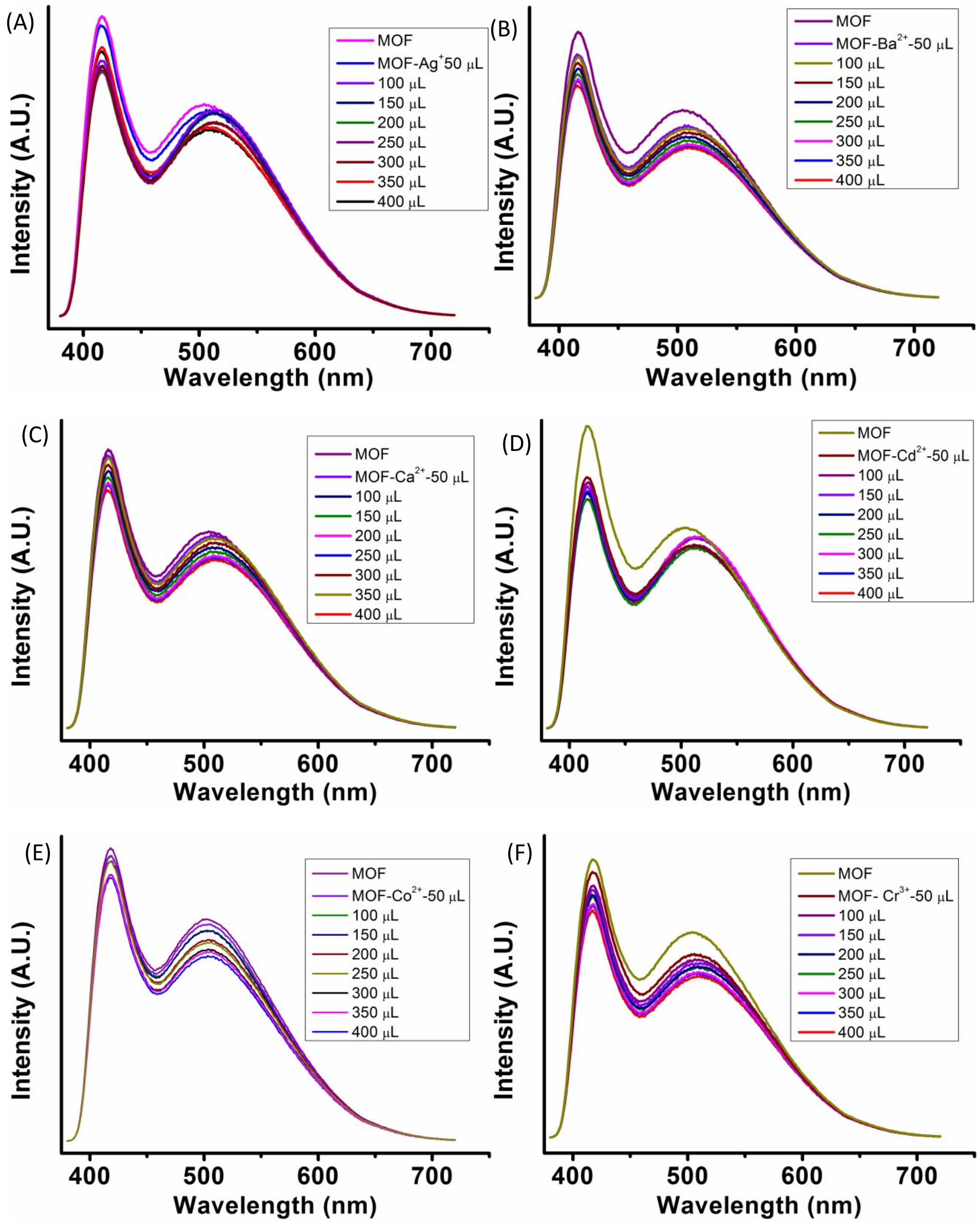

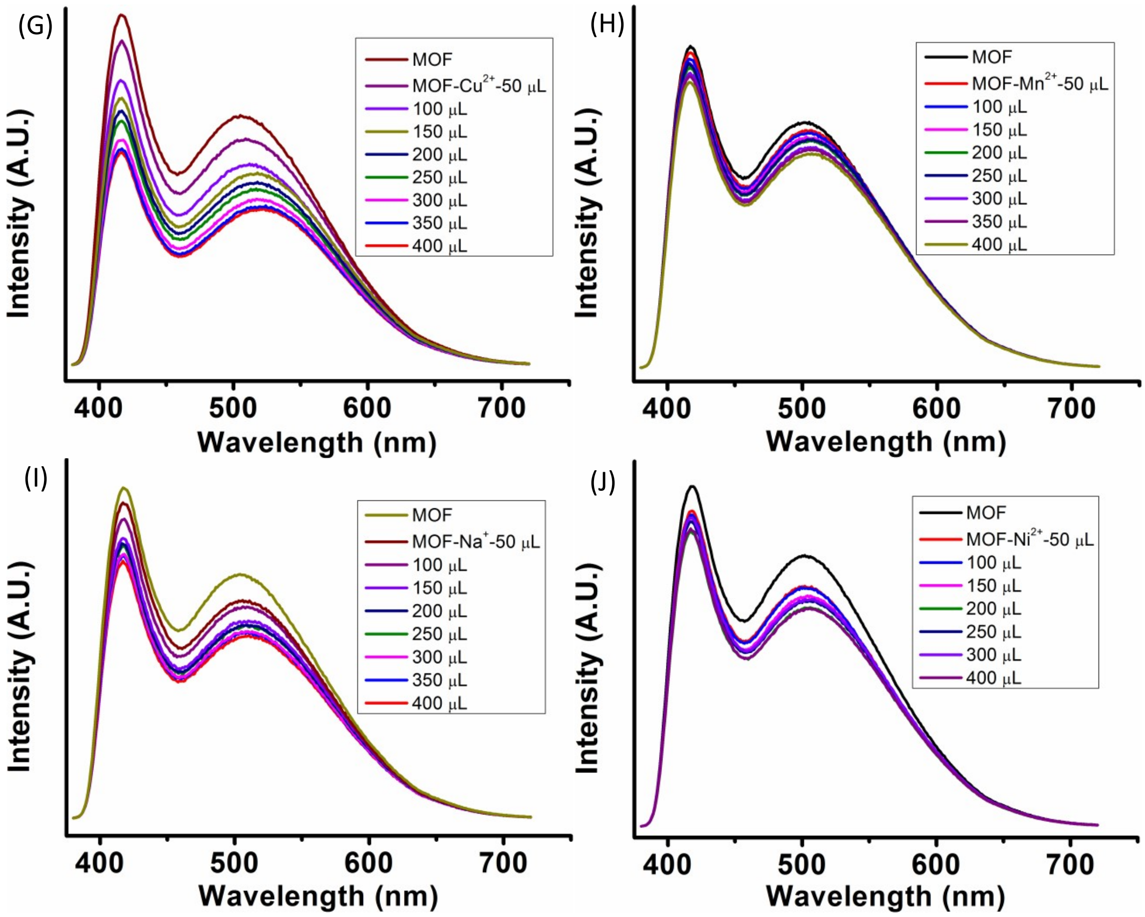

Figure 15S: The PL titration of MOF with metal ions (A) Ag^{+}, (B) Ba^{2+}, (C) Ca^{2+}, (D) Cd^{2+}, (E) Co^{2+}, (F) Cr^{3+}, (G) Cu^{2+}, (H) Mn^{2+}, (I) Na^{+}, (J) Ni^{2+}

17. **Effect on the UV-VIS of the MOF with addition of Al^{3+} in presence of other Metal ions**

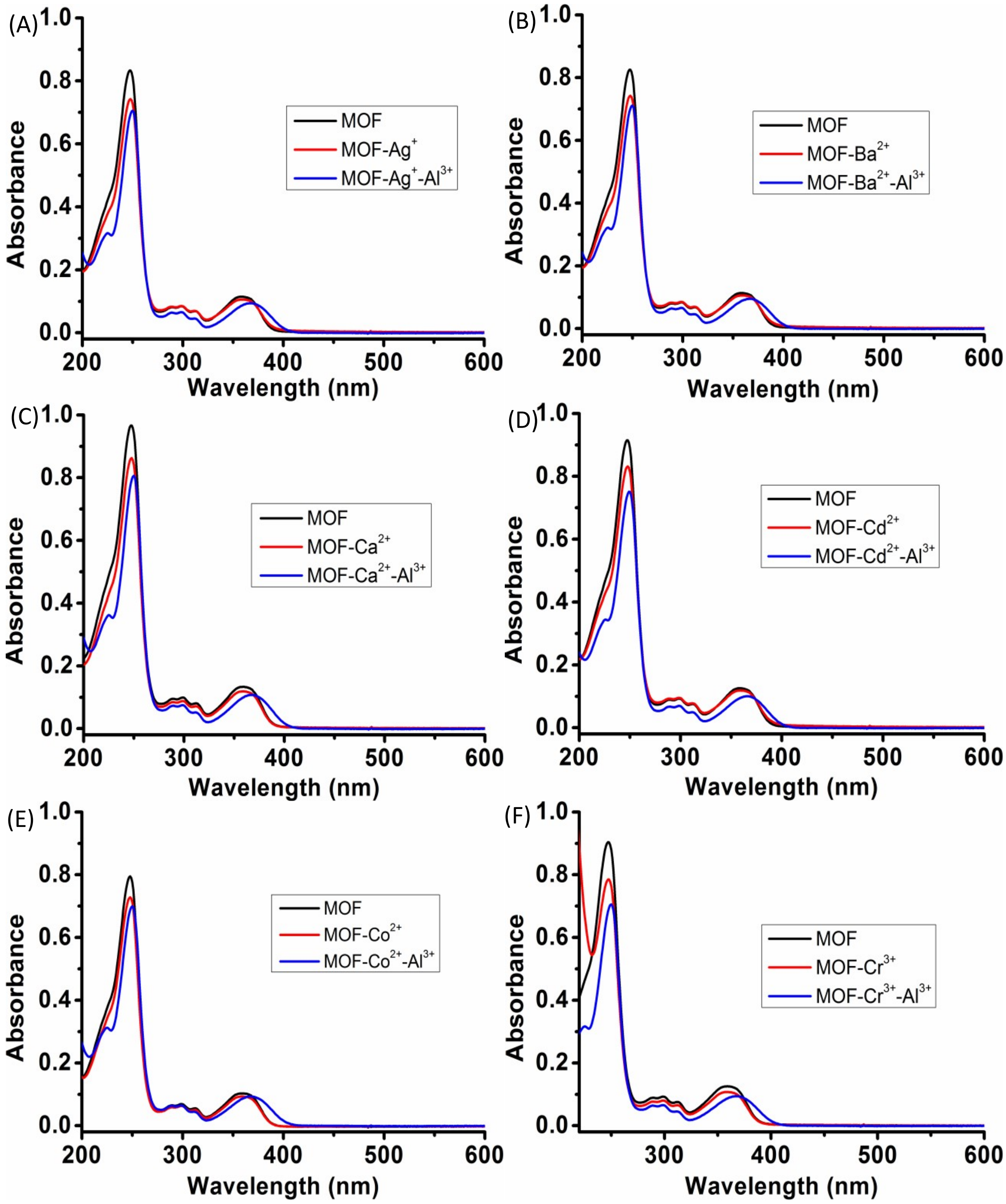

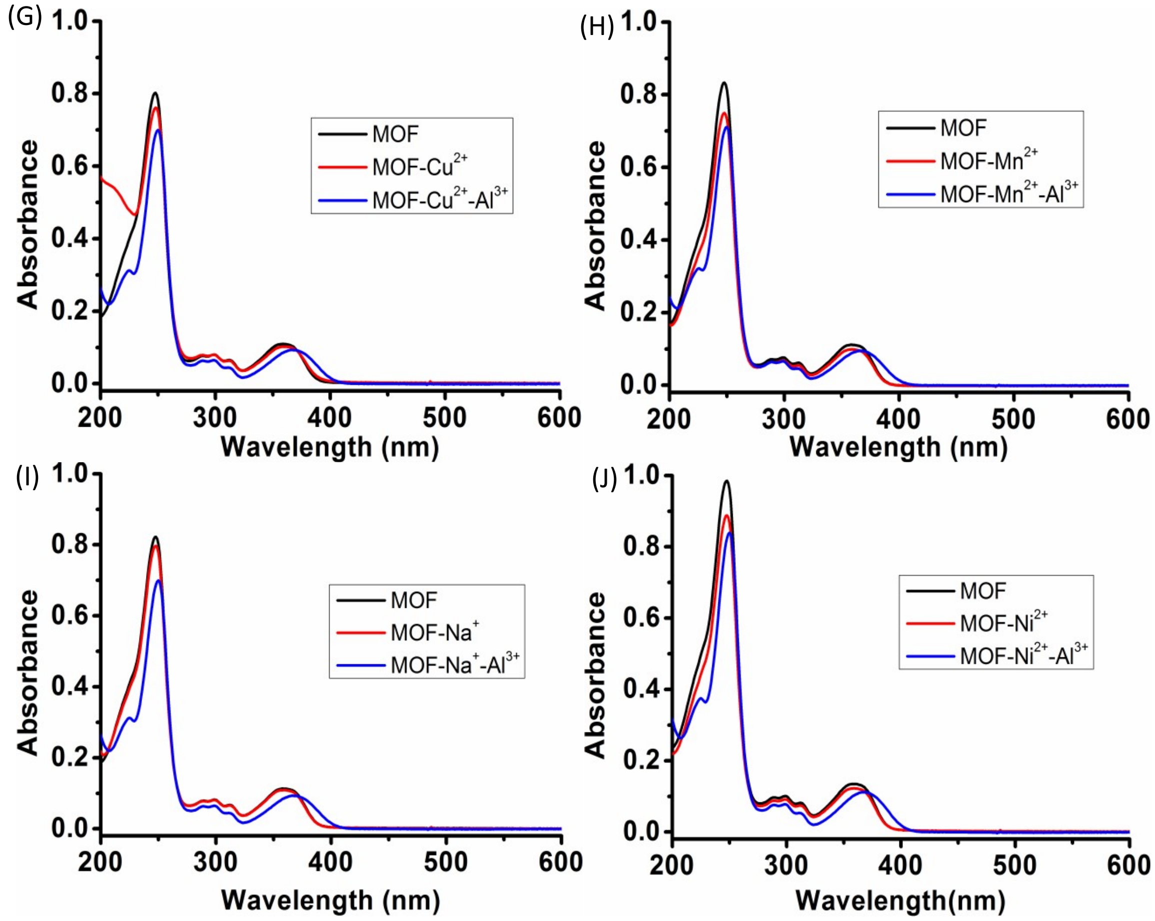

Figure 16S: Effect on UV-VIS curve nature of the MOF due to Al^{3+} addition in the MOF solution with metal ions (A) Ag^{+}, (B) Ba^{2+}, (C) Ca^{2+}, (D) Cd^{2+}, (E) Co^{2+}, (F) Cr^{3+}, (G) Cu^{2+}, (H) Mn^{2+}, (I) Na^{+}, (J) Ni^{2+}

18. Effect on the PL of the MOF with addition of Al^{3+} in presence of other Metal ions

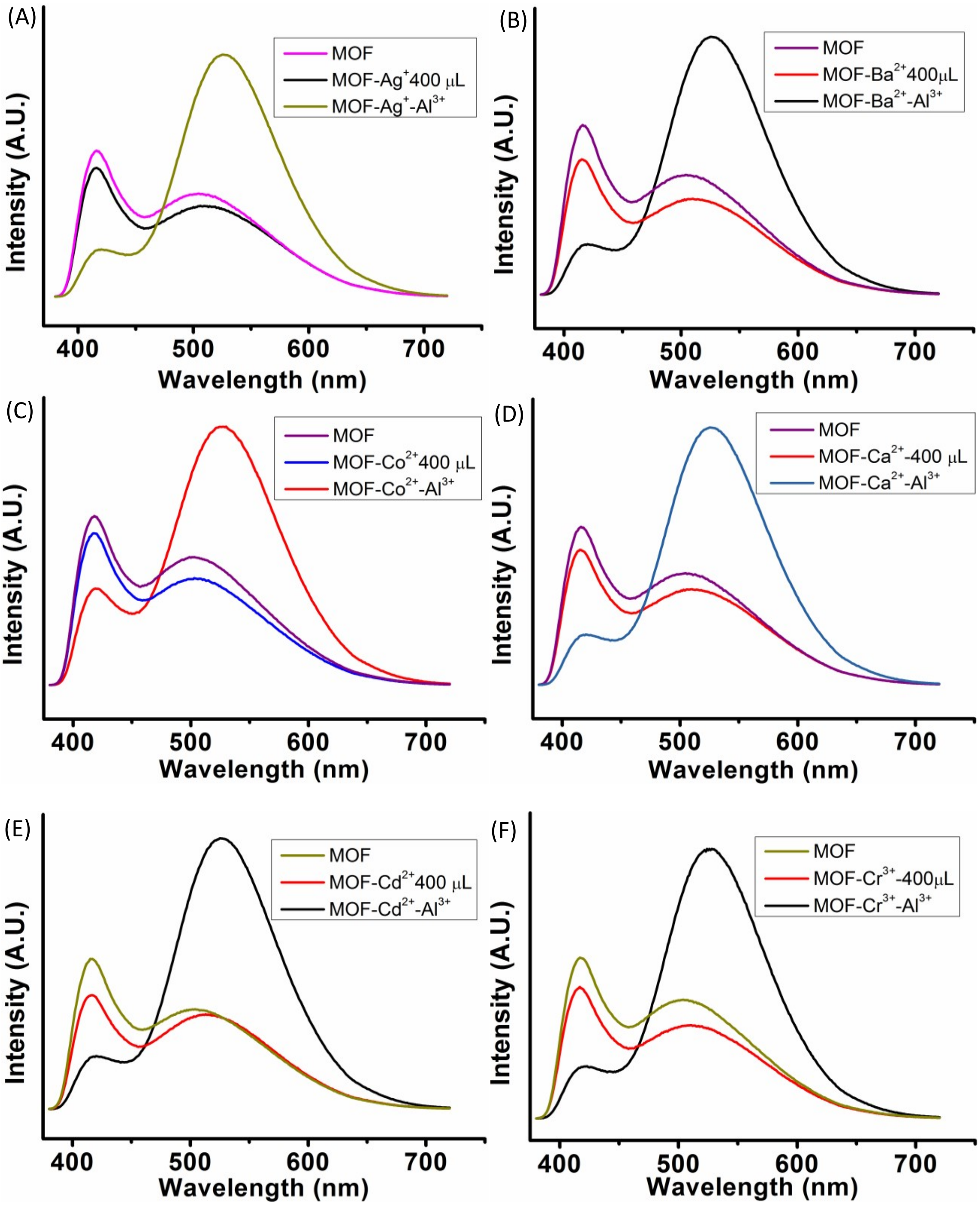

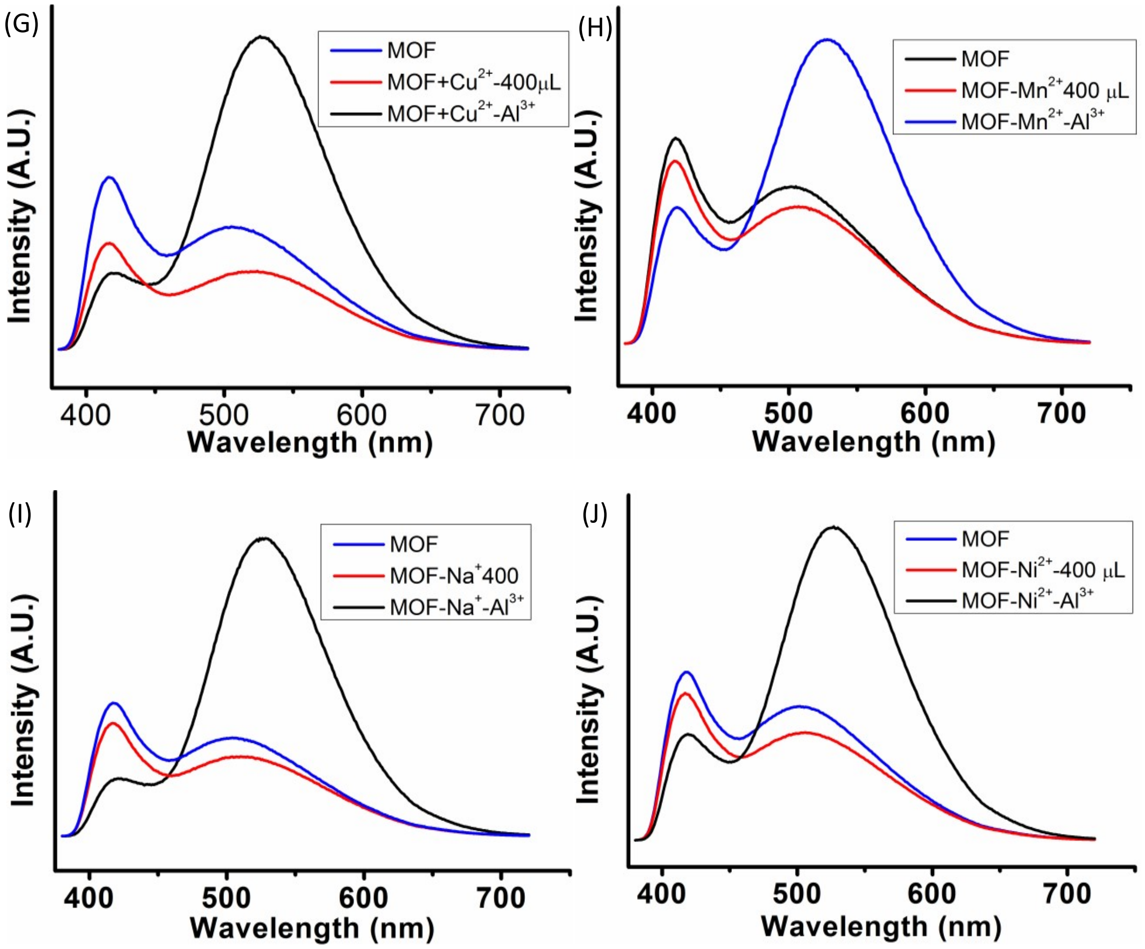

Figure 17S: Effect on PL curve nature of the MOF due to Al^{3+} addition in the MOF solution with metal ions (A) Ag^{+}, (B) Ba^{2+}, (C) Ca^{2+}, (D) Cd^{2+}, (E) Co^{2+}, (F) Cr^{3+}, (G) Cu^{2+}, (H) Mn^{2+}, (I) Na^{+}, (J) Ni^{2+}

19. **Time-correlated Single Photon Counting of MOF in presence of Al^{3+} ion**

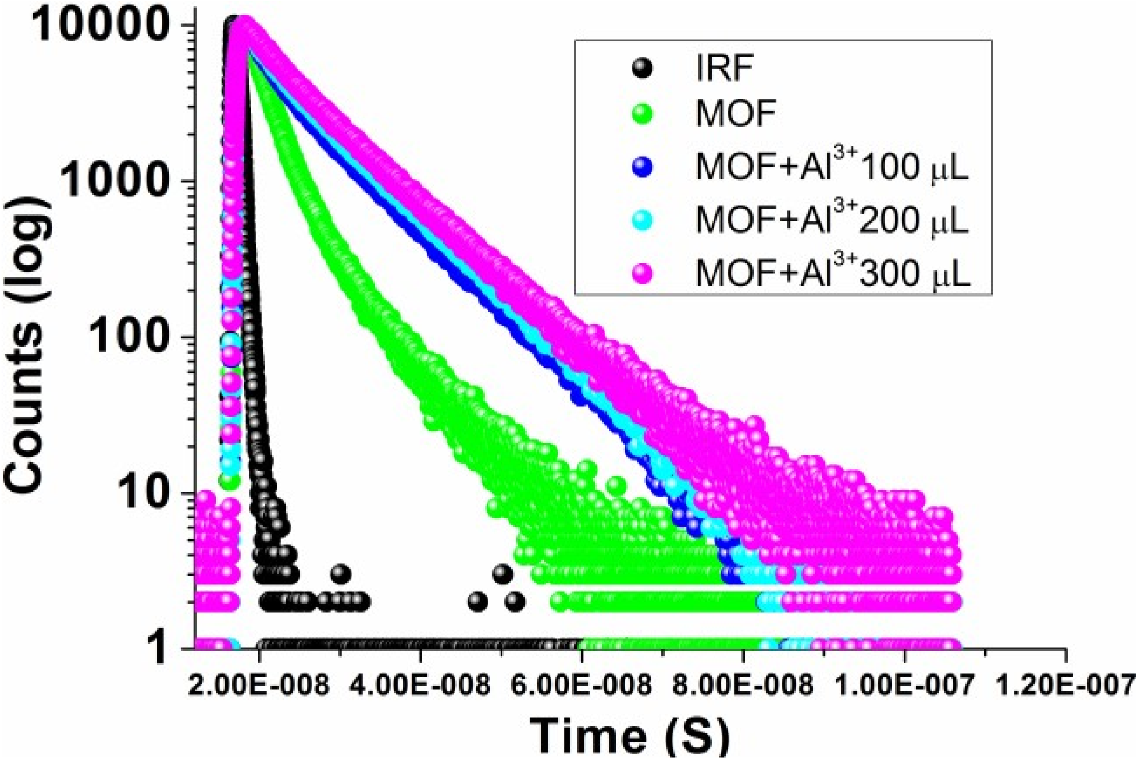

Figure 18S: The fluorescence decay of MOF and MOF solution with Al^{3+} metal ion.

20. Dynamic light scattering (particle size) of the MOF in presence Al^{3+} ions

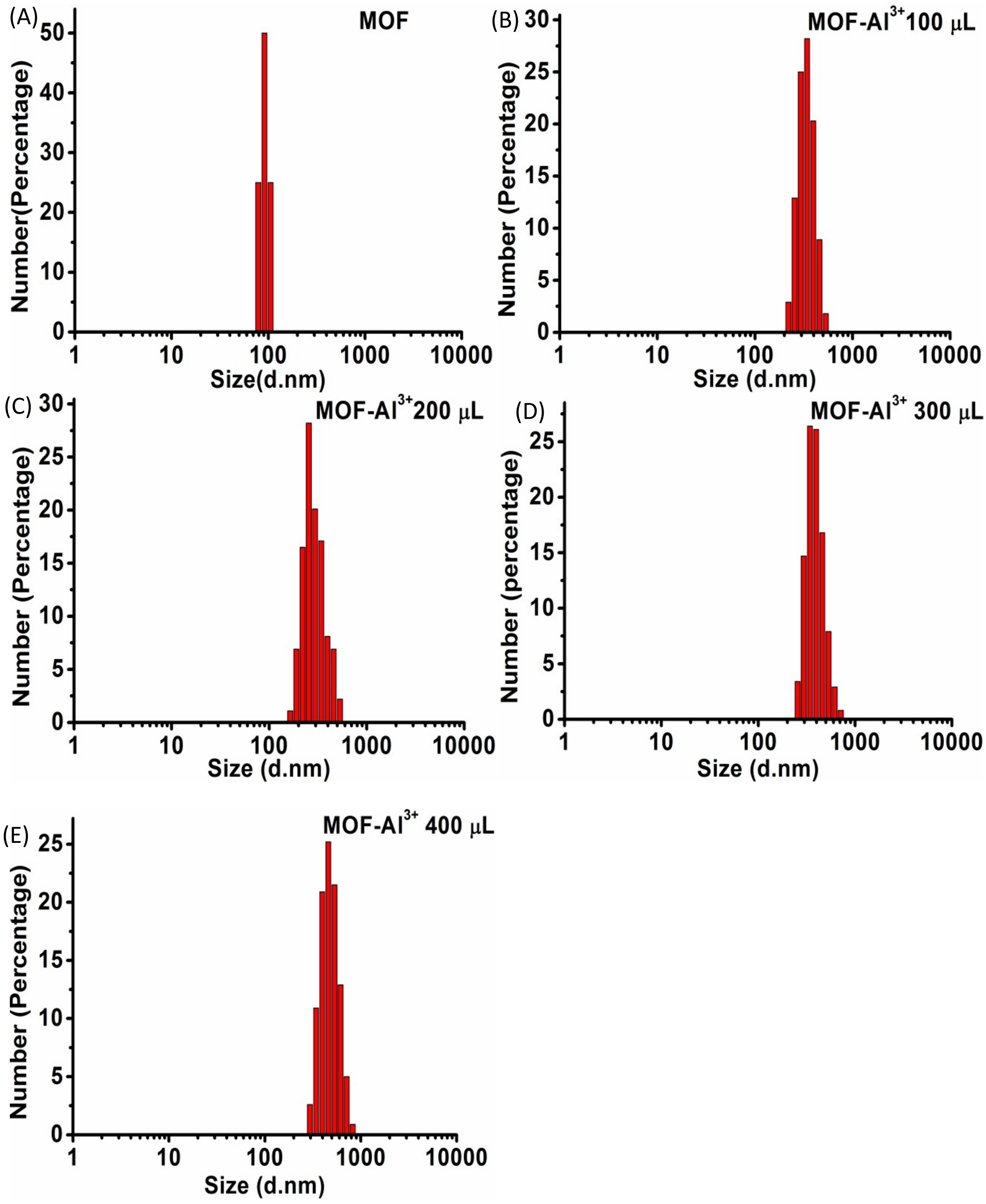

Figure 19S: The particle Size (DLS) of the MOF and MOF in presence of Al^{3+} metal ion

21. Zeta potential of the MOF in presence of Al^{3+} ions

MOF

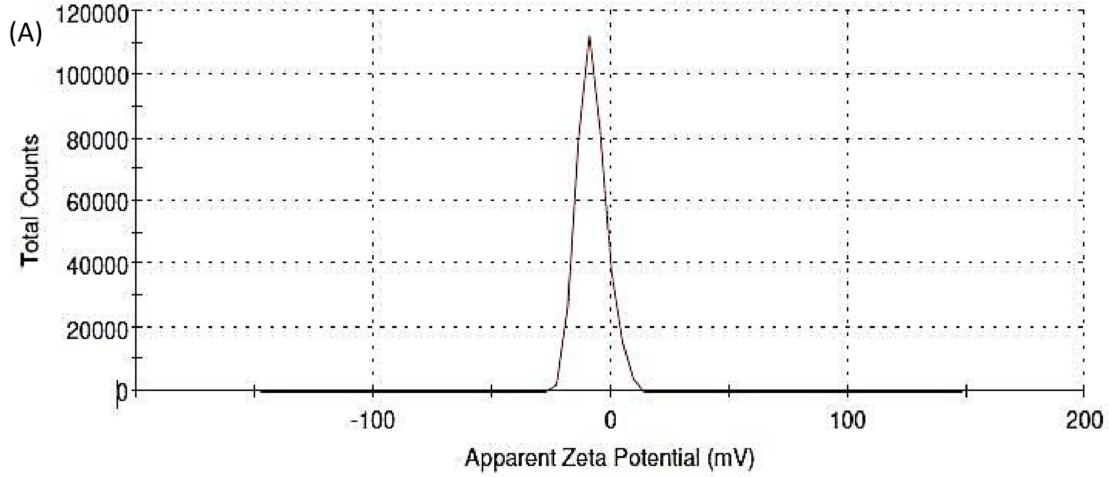

M

OF-Al^{3+} 100 µL

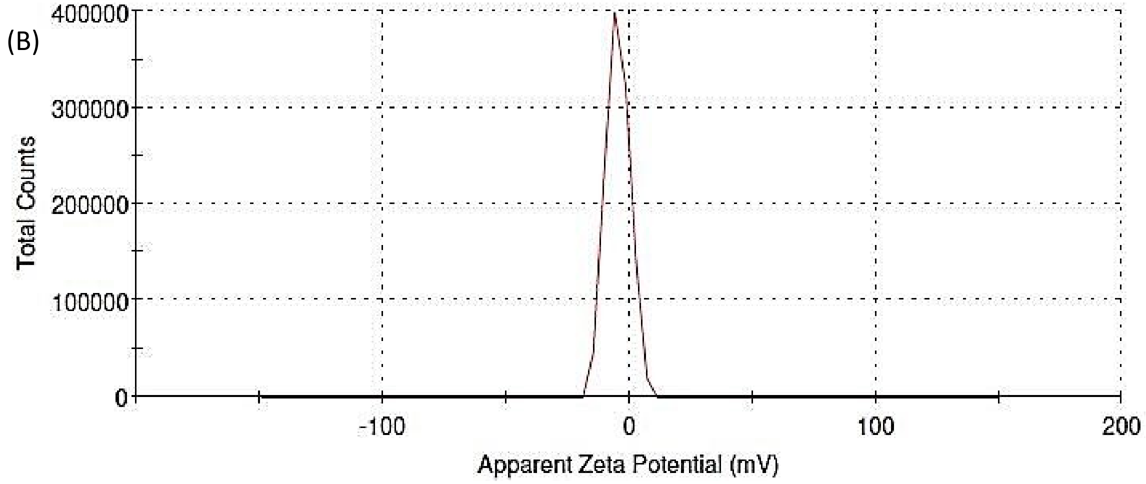

MOF-Al^{3+} 200 µL

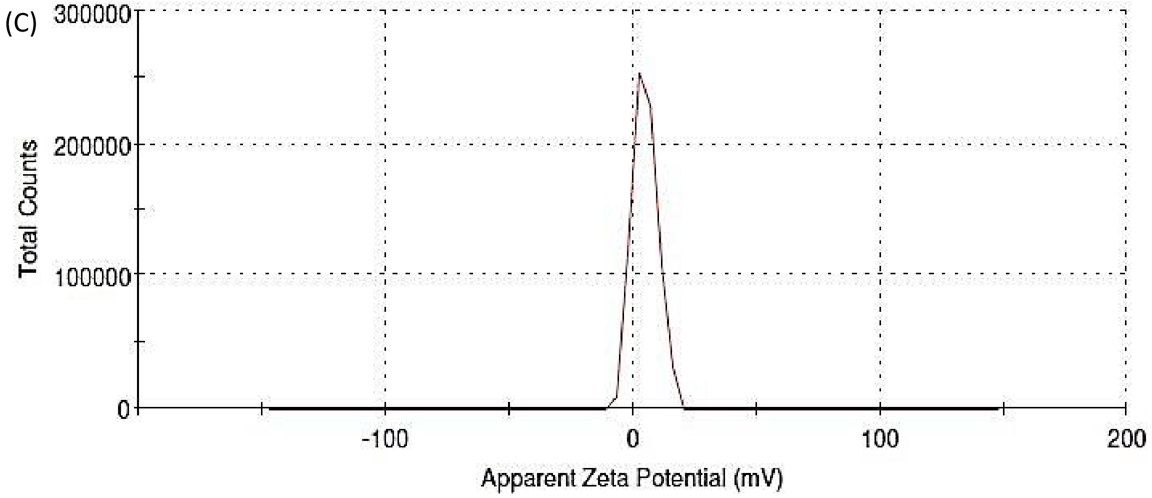

MOF-Al^{3+} 300 µL

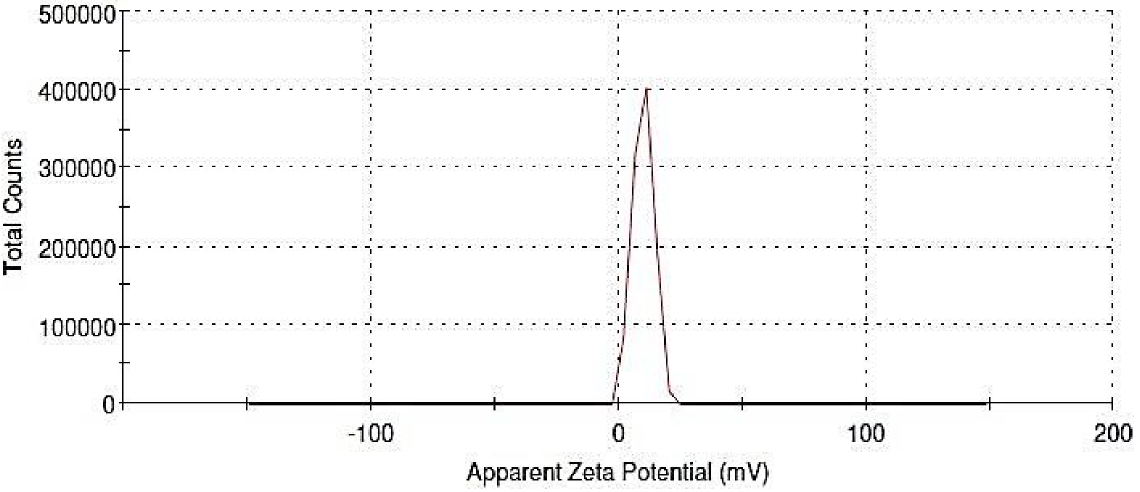

MOF-Al^{3+} 400 µL

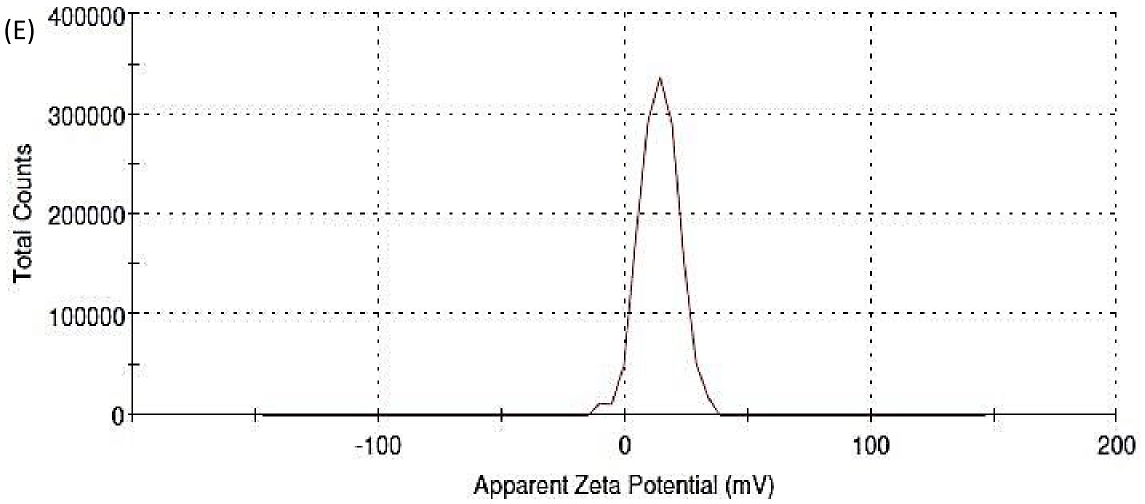

Figure 20S: The Zeta potential (DLS) of the MOF and Al^{3+} solution of MOF

22. Effect on the UV-VIS of the MOF with addition of Fe^{3+} in presence of other Metal ions

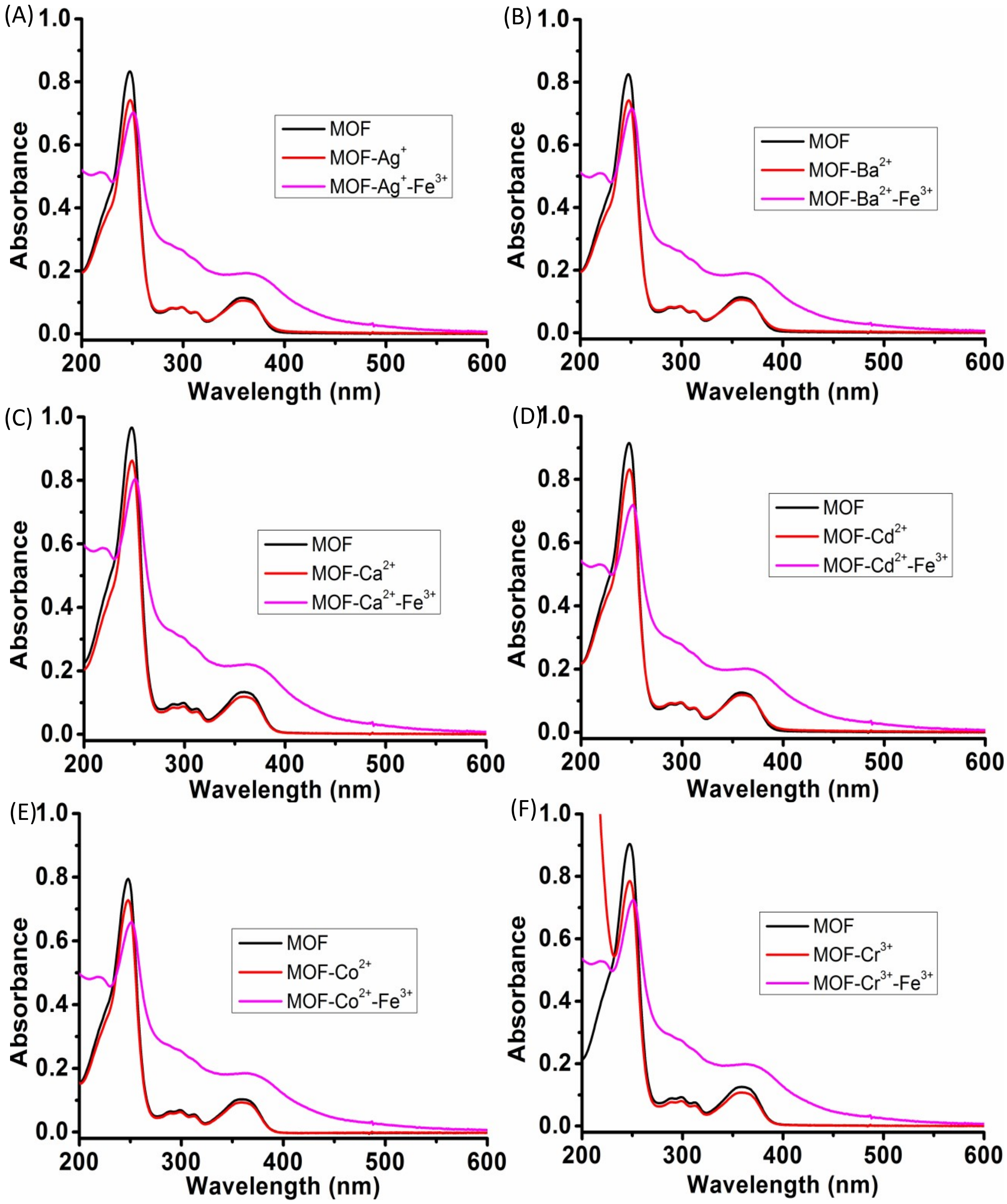

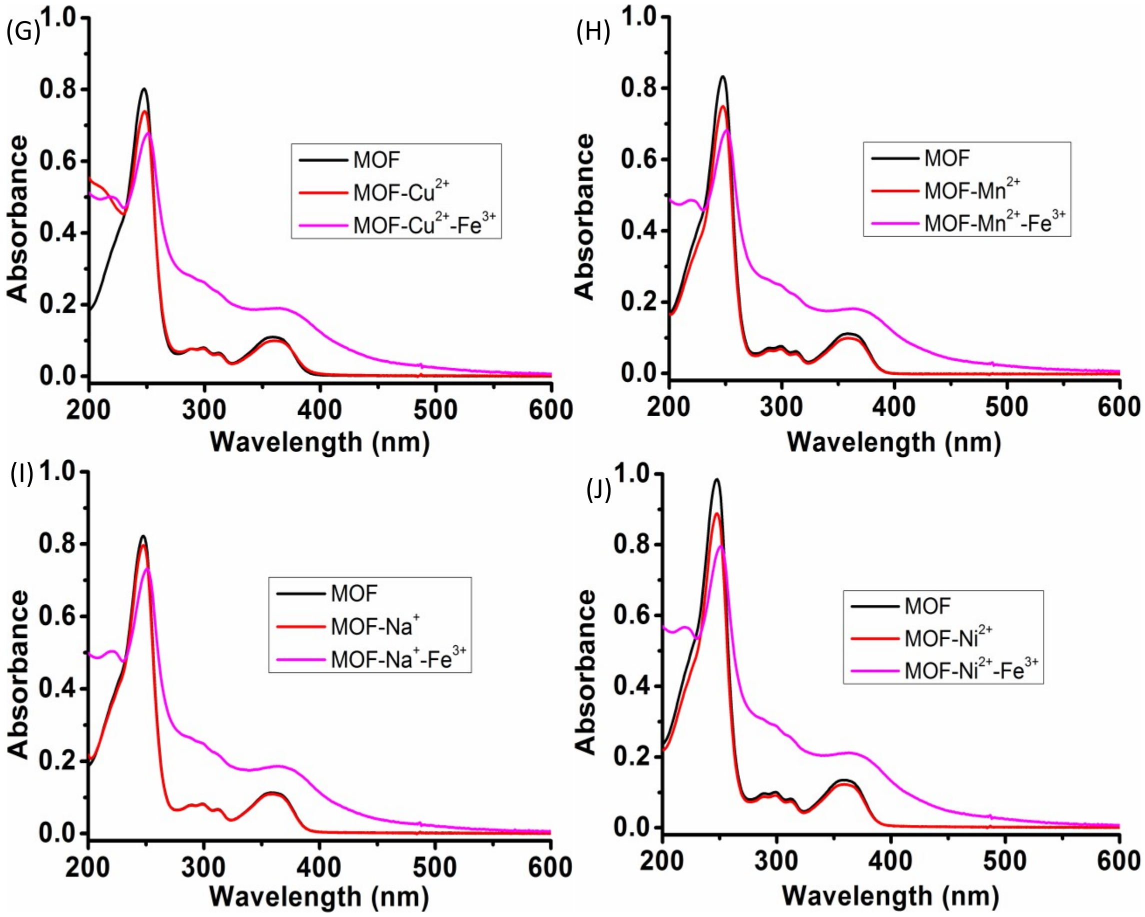

Figure 21S: Effect on UV-VIS curve nature of the MOF due to Fe^{3+} addition in the MOF solution with metal ions (A) Ag^{+}, (B) Ba^{2+}, (C) Ca^{2+}, (D) Cd^{2+}, (E) Co^{2+}, (F) Cr^{3+}, (G) Cu^{2+}, (H) Mn^{2+}, (I) Na^{+}, (J) Ni^{2+}

23. Effect on the PL of the MOF with addition of Fe^{3+} in presence of other Metal ions

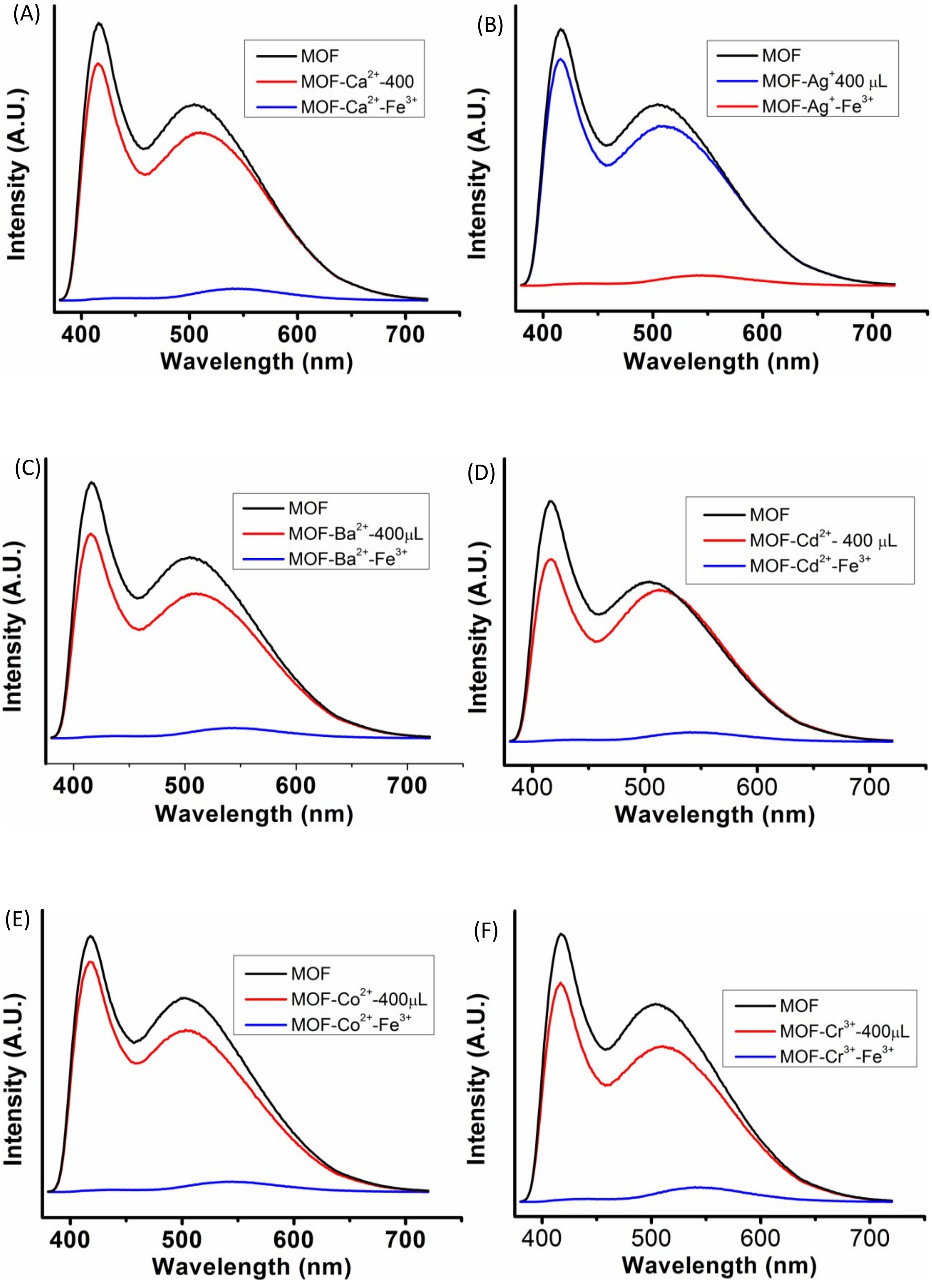

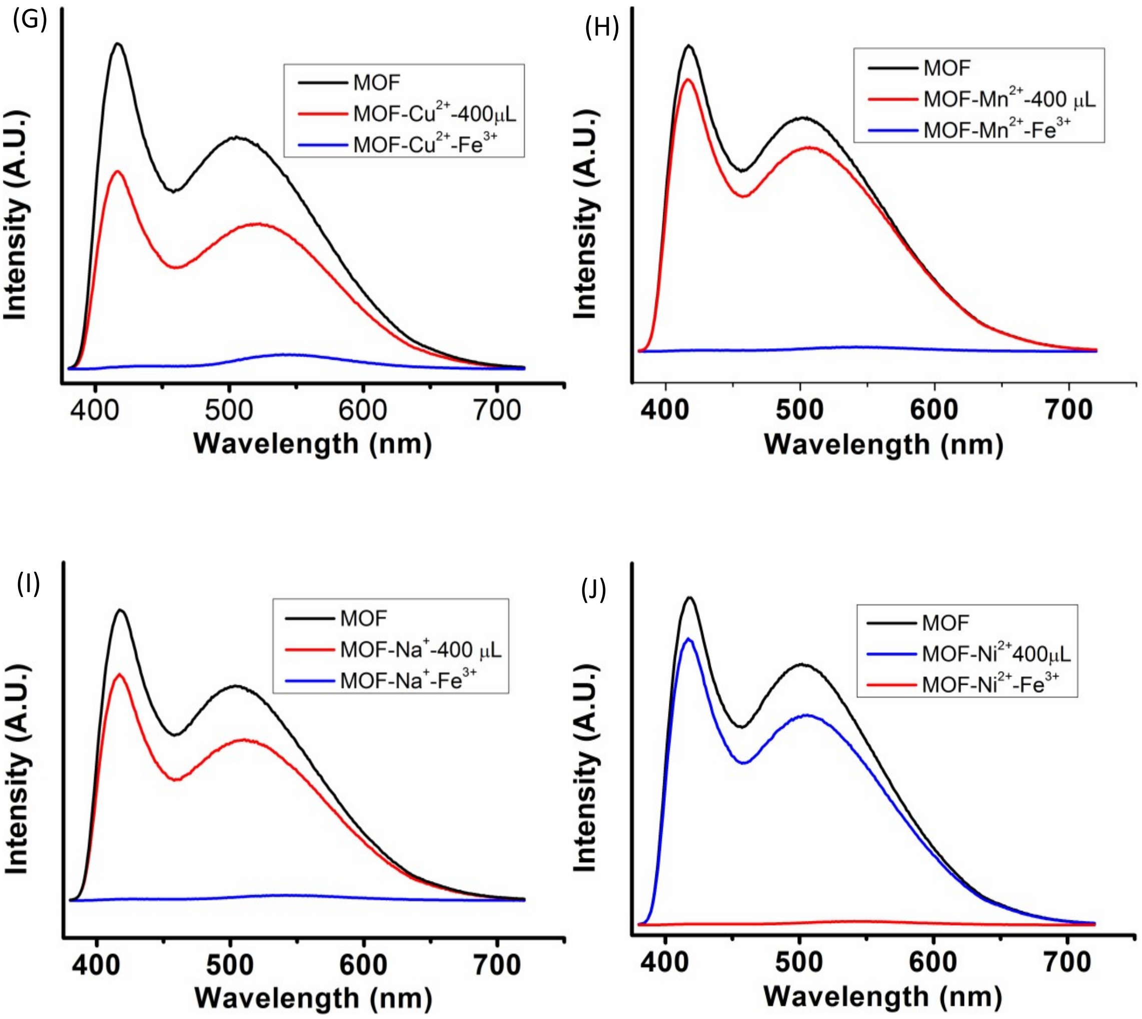

Figure 22S: Effect on PL curve nature of the MOF due to Al^{3+} addition in the MOF solution with metal ions (A) Ag^{+}, (B) Ba^{2+}, (C) Ca^{2+}, (D) Cd^{2+}, (E) Co^{2+}, (F) Cr^{3+}, (G) Cu^{2+}, (H) Mn^{2+}, (I) Na^{+}, (J) Ni^{2+}

24. **Dynamic light scattering (particle size) of the MOF in presence Fe^{3+} ion**

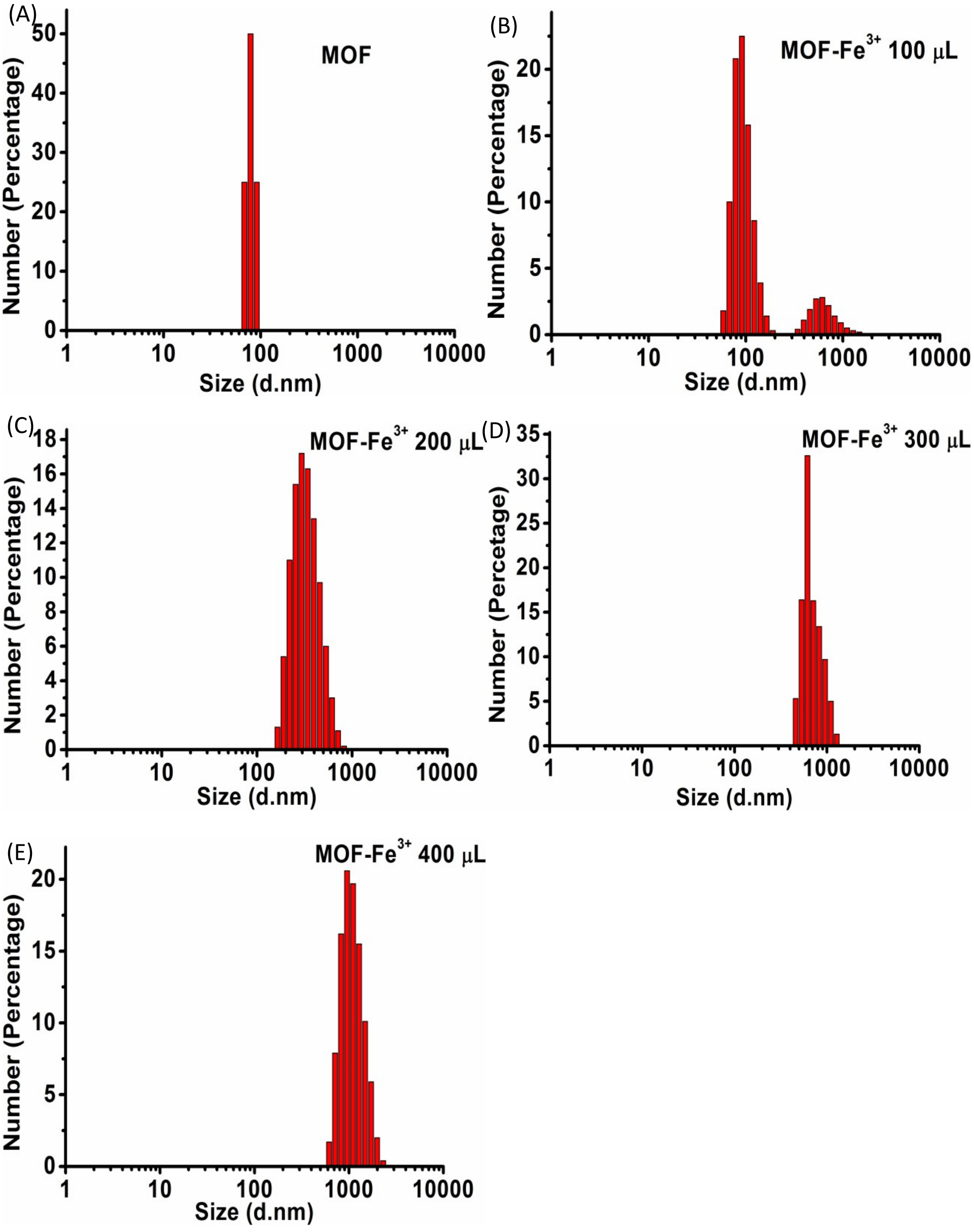

Figure 23S: The particle Size (DLS) of the MOF and MOF in presence of Fe^{3+} metal ion

25. Zeta potential of the MOF in presence of Fe^{3+} ions MOF

MOF

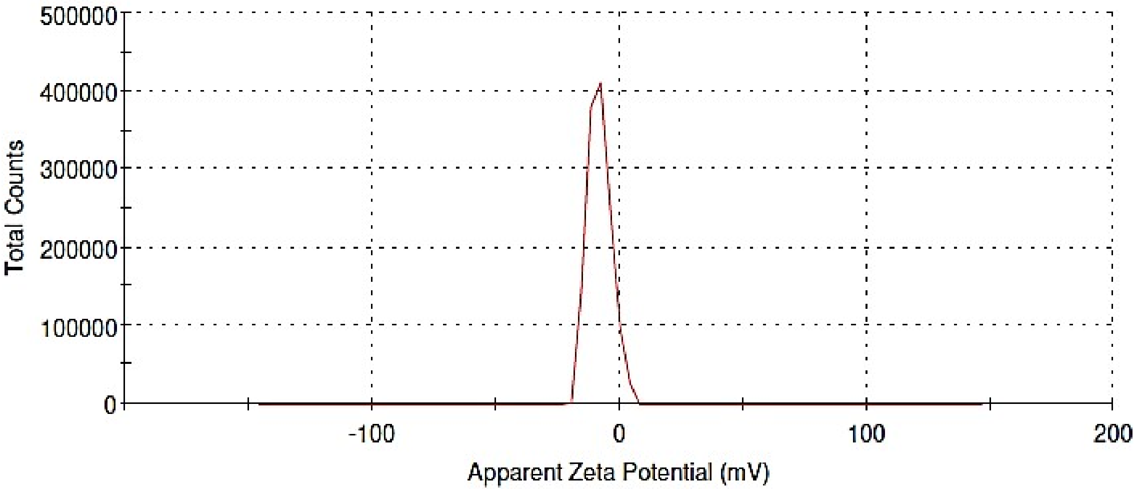

MOF-Fe^{3+} 100 µL

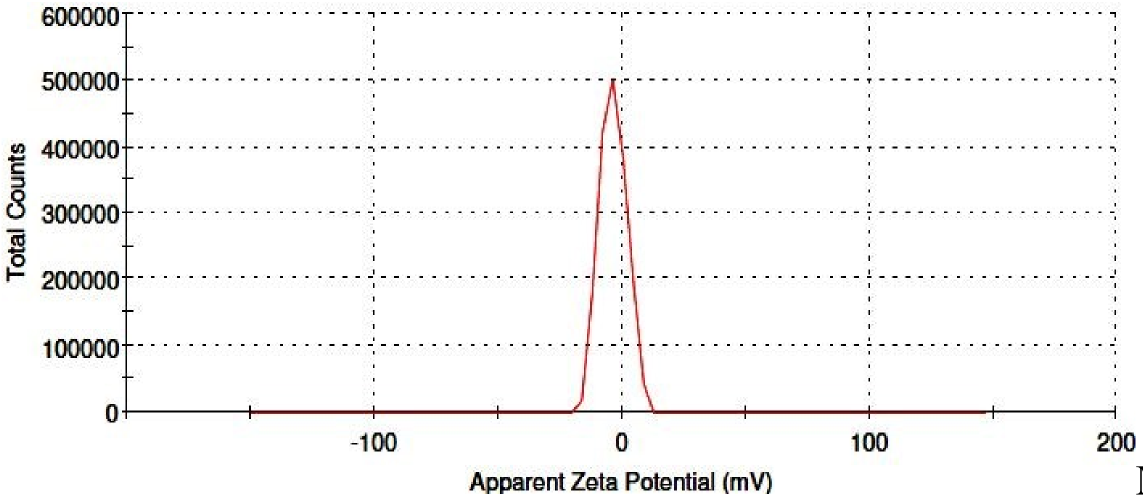

M

OF-Fe^{3+} 200µL

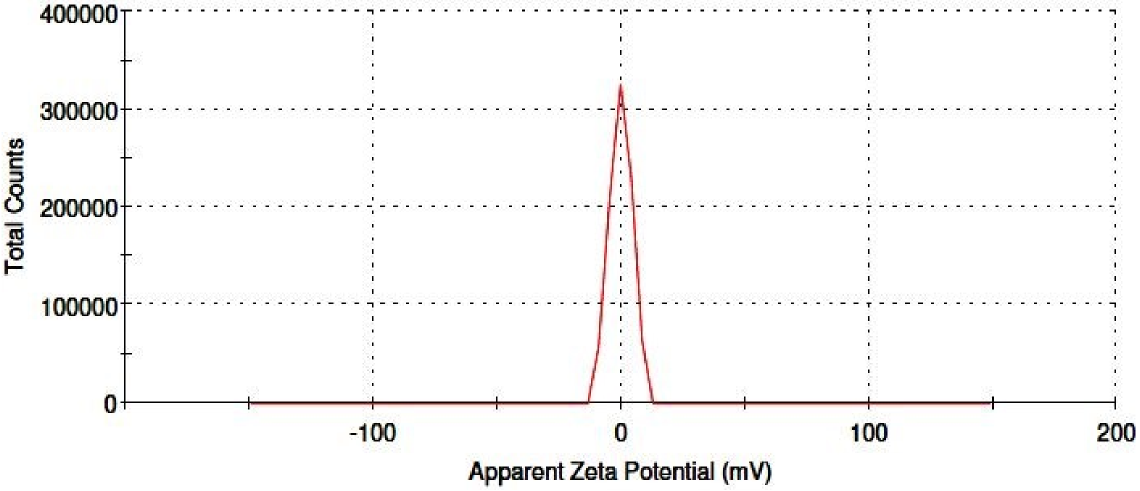

MOF-Fe^{3+} 300 µL

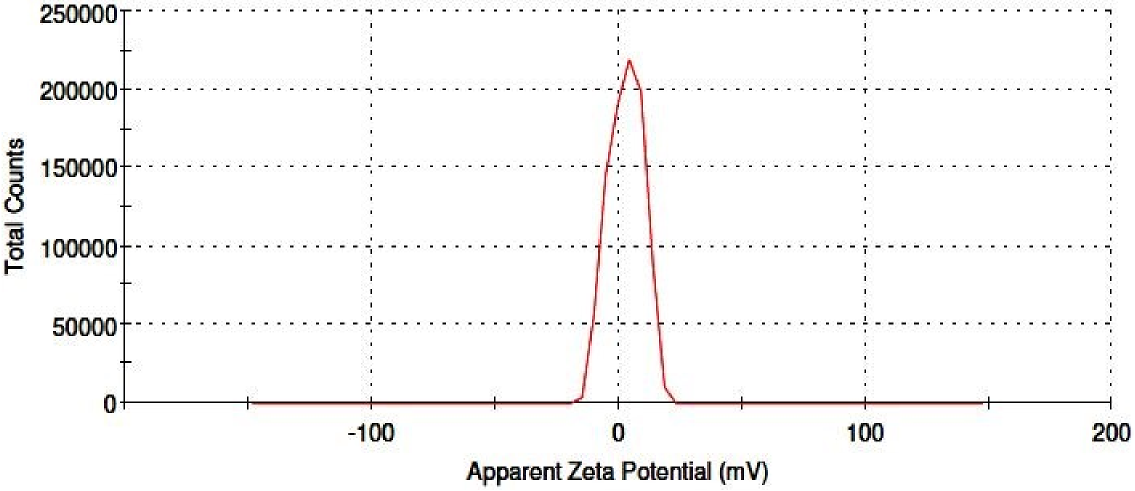

MOF-Fe^{3+} 400 µL

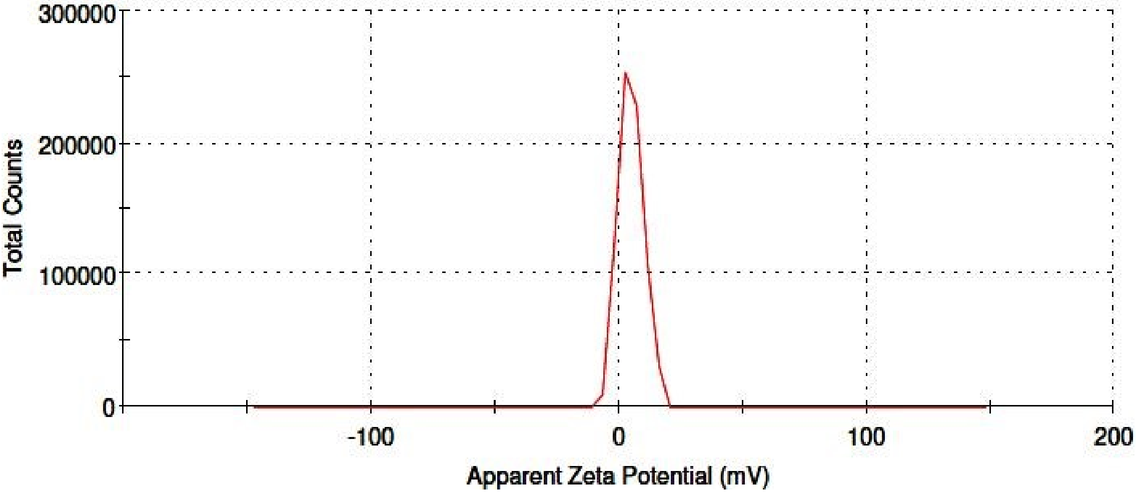

Figure 24S: The Zeta potential (DLS) of the MOF and Fe^{3+} solution of MOF

26. Stability of MOF in different solutions

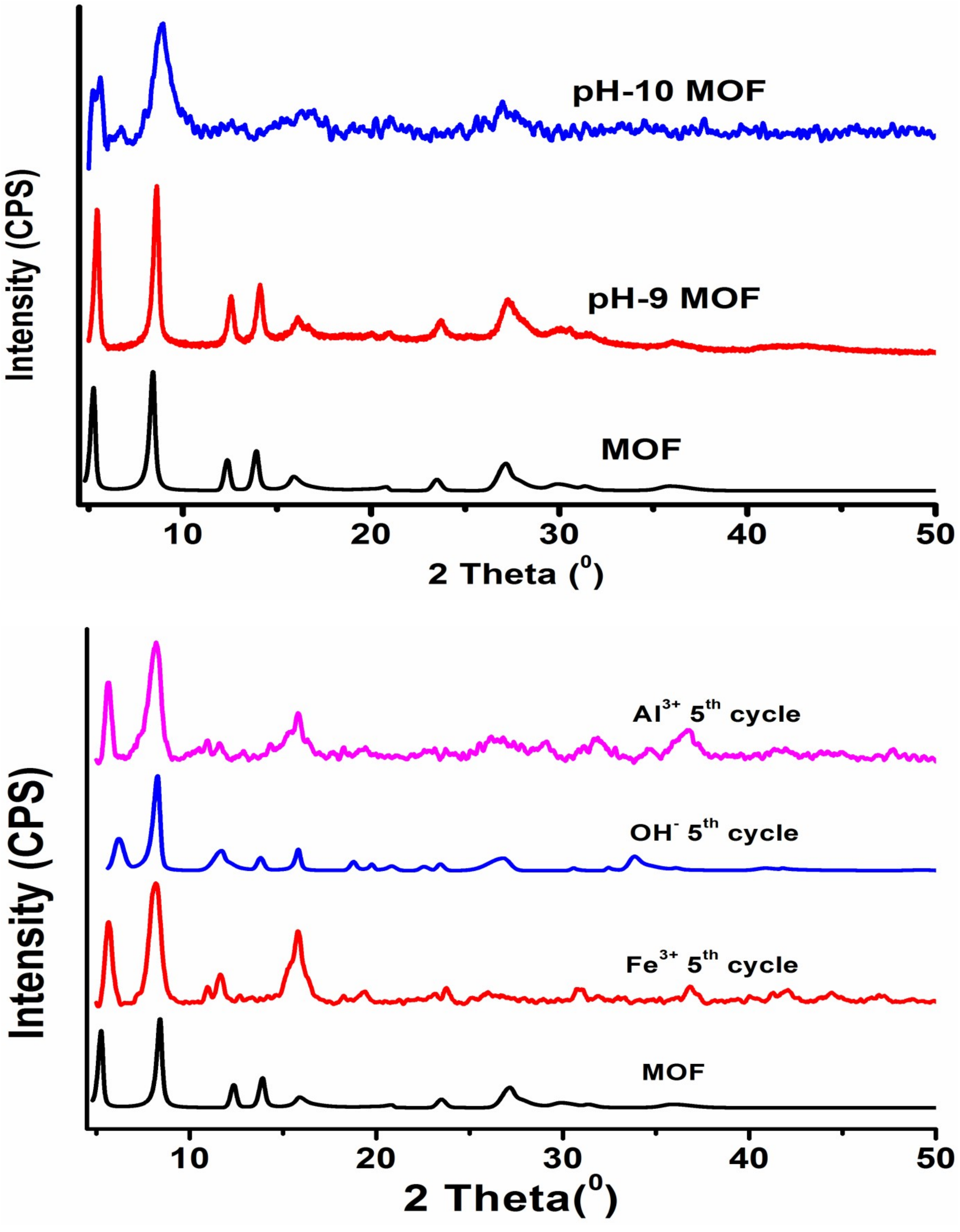

Figure 25S: PXRD of the MOF retrieved from different solution

27. The Stern-Volmer plot of the MOF calculated from titrations with Al^{3+} and Fe^{3+}

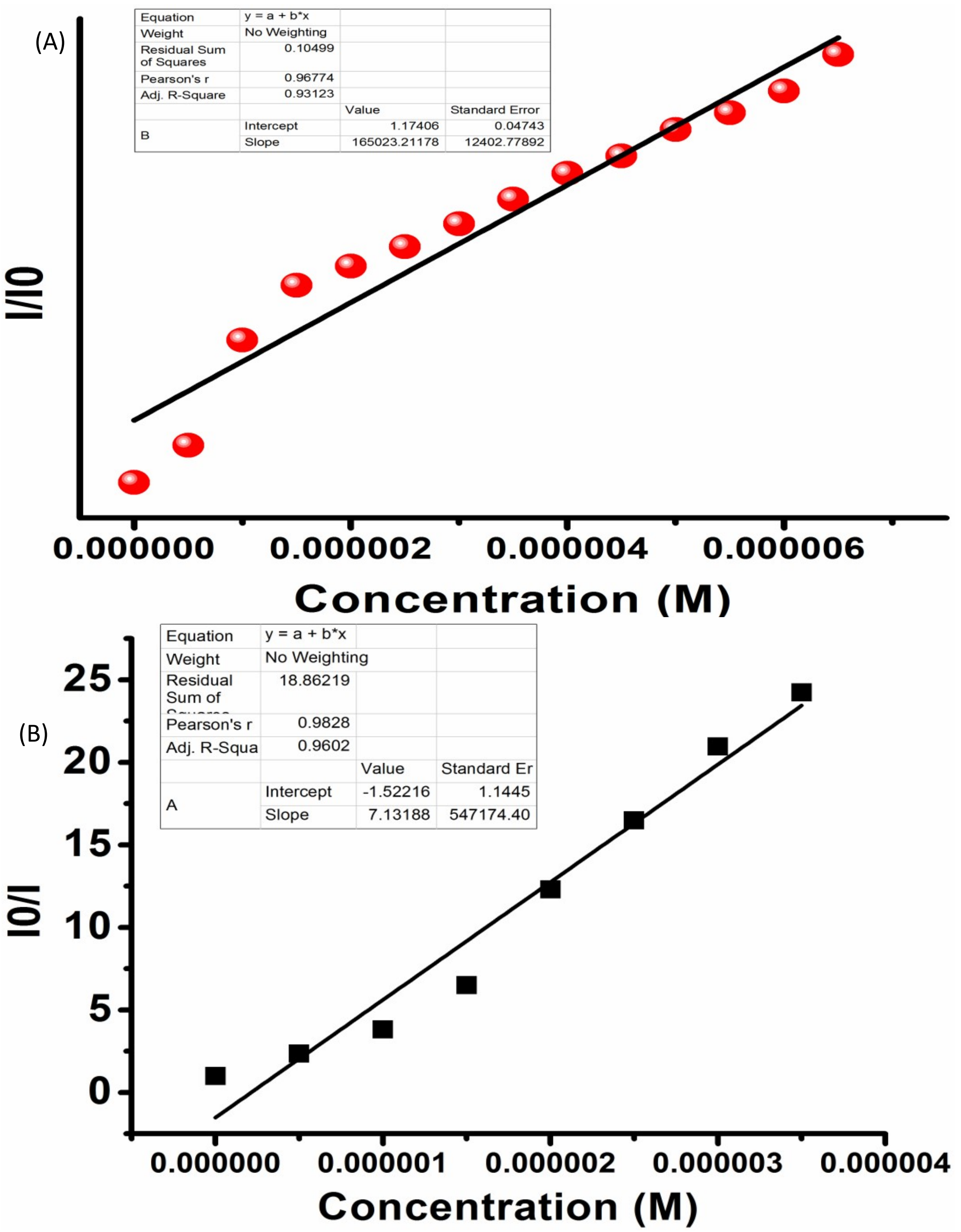

Figure 26S: (A) Represents the K_{SV} plot of MOF titrated with Al^{3+} solution. (B) Represents the K_{SV} plot of Fe^{3+} titration.

28. UV-VIS titration of Ligand with OH^-

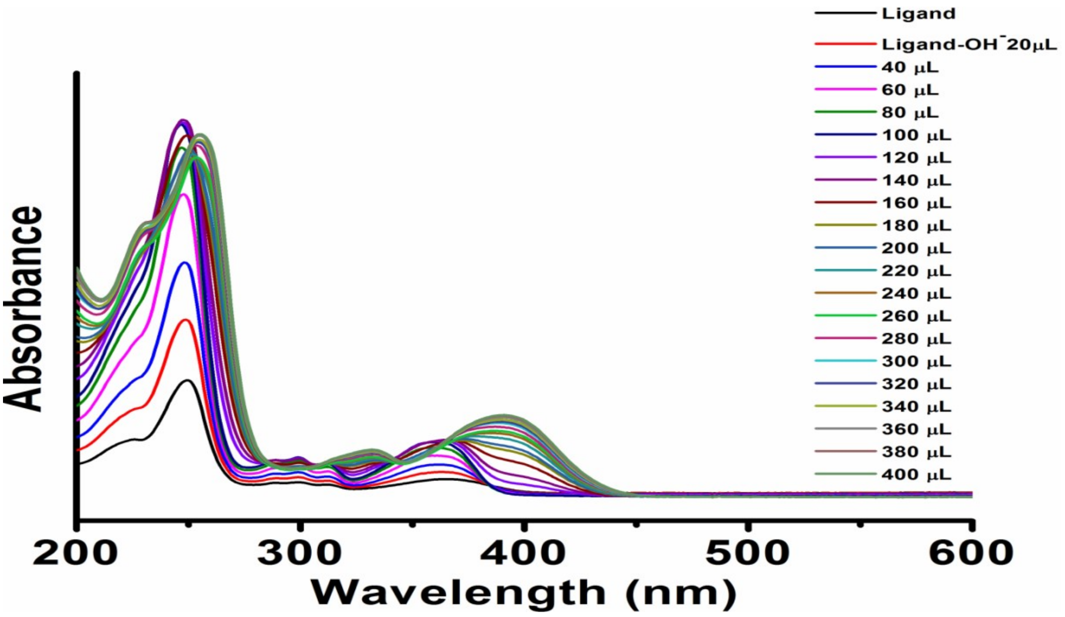

Figure 27S: The UV-VIS titration of 4, 8-dihydroxynaphthalene-2, 6-dicarboxylic acid with OH^- anion.

29. PL titration of Ligand with OH^-

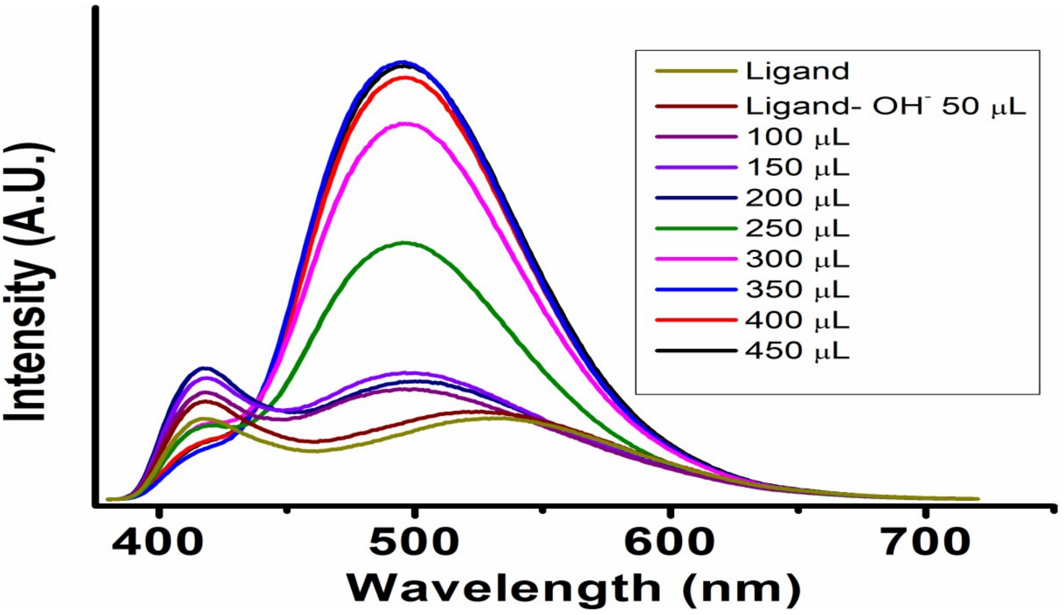

Figure 28S: The PL titration of 4, 8-dihydroxynaphthalene-2, 6-dicarboxylic acid with OH^- anion.

30. Titration of the Al^{3+}@MOF with Fe^{3+} ion solution

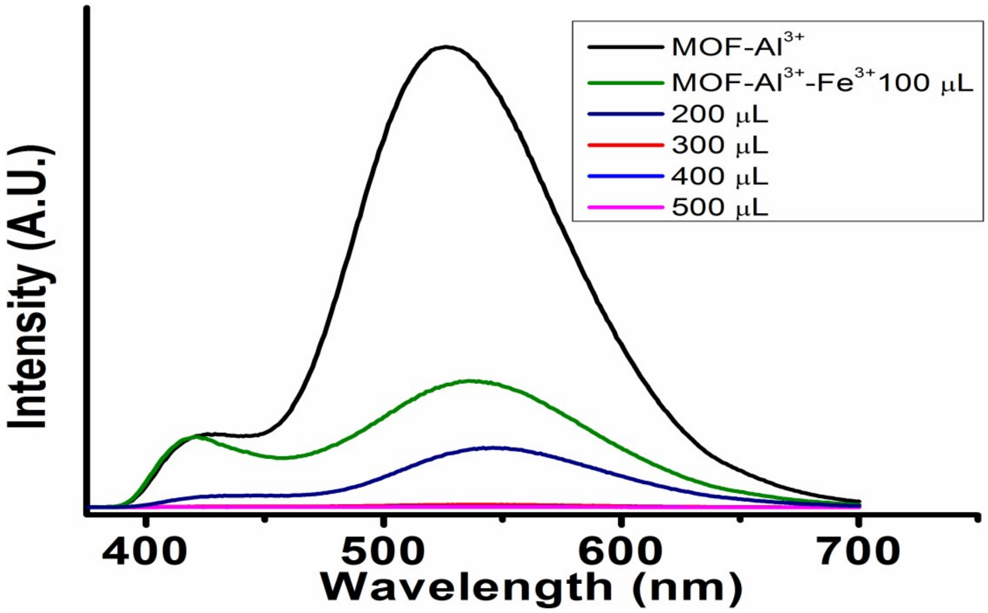

Figure 29S: The PL titration of Al^{3+}@MOF with Fe^{3+} ions.

31. Titration of the Fe^{3+}@MOF with Al^{3+} ion solution

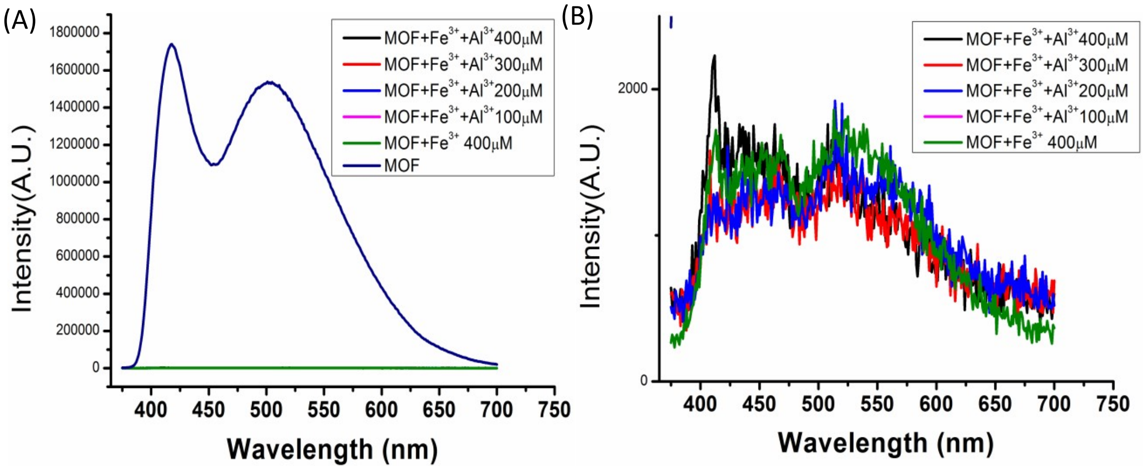

Figure 30S: The PL titration curves of Fe^{3+}@MOF with Al^{3+} ions (A) with MOF emissions, (B) without MOF emissions.

32. Fluorescence Intensity of the MOF in presence of Al^{3+} and calculation of LOD

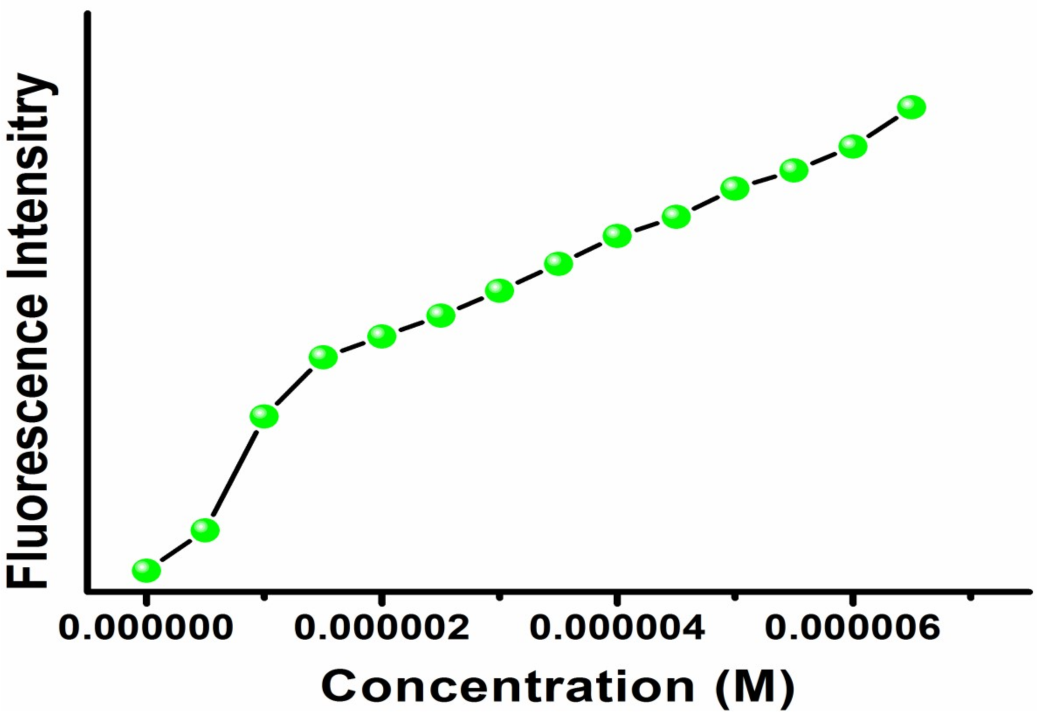

Regression Statistics								
Multiple R	0.999435054							
R Square	0.998870427							
Adjusted R Square	0.99872923							
Standard Error	10664.42249							
Observations	10							
ANOVA								
	df	*SS*	*MS*	*F*	*Significance F*			
Regression	1	8.04562E+11	8.04562E+11	7074.32017	4.4536E-13			
Residual	8	909839255.8	113729907					
Total	9	8.05472E+11						
	Coefficients	*Standard Error*	*t Stat*	*P-value*	*Lower 95%*	*Upper 95%*	*Lower 95.0%*	*Upper 95.0%*
Intercept	2163228.418	6268.054987	345.1195662	5.5636E-18	2148774.26	2177682.58	2148774.26	2177682.58
X Variable 1	1.97507E+11	2348228547	84.10897794	4.4536E-13	1.9209E+11	2.0292E+11	1.9209E+11	2.0292E+11

LOD for Al^{3+} is 3* standard error/co-efficient = 9.52×10^{-8} M

Figure 31S: calculation of LOD for Al^{3+} using the regression method.

33. Fluorescence Intensity of the MOF in presence of Fe^{3+} and calculation of LOD

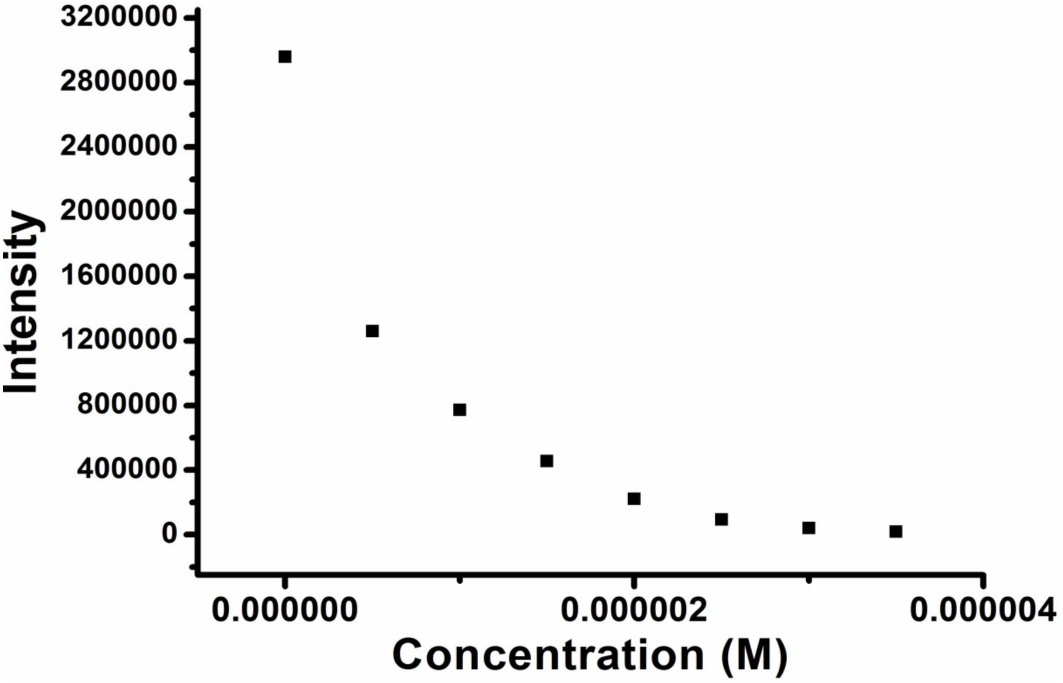

SUMMARY OUTPUT

Regression Statistics	
Multiple R	0.988089032
R Square	0.976319935
Adjusted R Squa	0.96842658
Standard Error	110254.1788
Observations	5

ANOVA

	df	SS	MS	F	Significance F
Regression	1	1.50356E+12	1.50356E+12	123.6888408	0.00155768
Residual	3	36467951859	12155983953		
Total	4	1.54003E+12			

	Coefficients	Standard Error	t Stat	P-value	Lower 95%	Upper 95%	Lower 95.0%	Upper 95.0%
Intercept	1669477	8540.519704	19.54833424	0.000292459	1397688.07	1941265.93	1397688.07	1941265.93
X Variable 1	-7.75515E+11	69730865341	-11.12154849	0.001557676	-9.9743E+11	-5.536E+11	-9.9743E+11	-5.536E+11

LOD for Fe^{3+} is 3* standard error/co-efficient = $3.3x10^{-8}$ M

Figure 32S: calculation of LOD for Fe^{3+} using the regression method

34. Fluorescence Intensity of the MOF in presence of OH^- and calculation of LOD

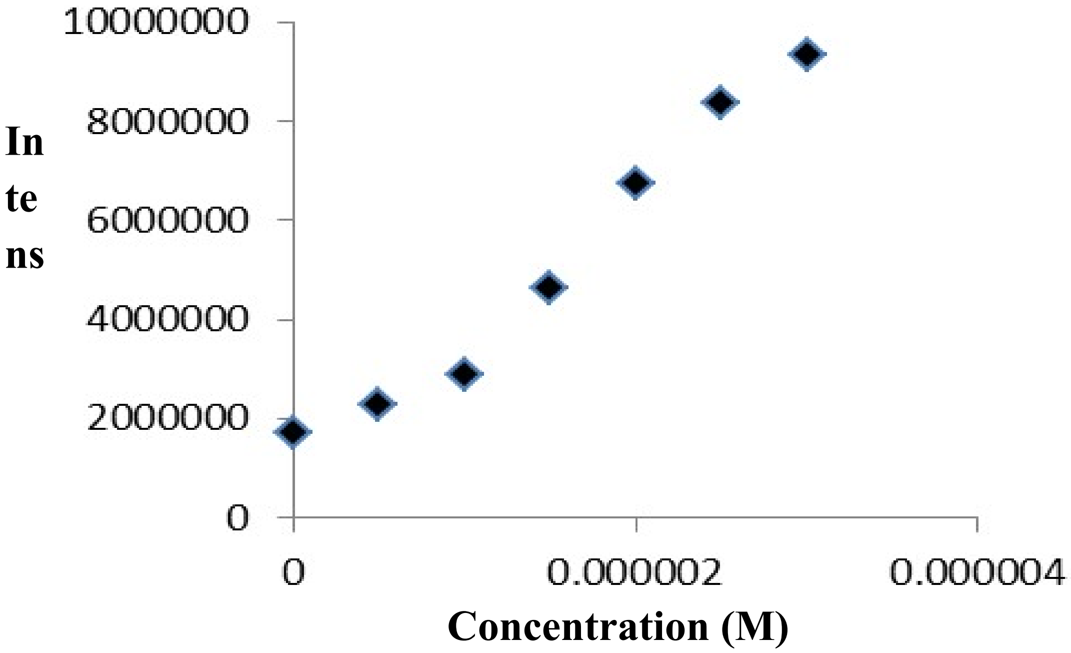

SUMMARY OUTPUT								
Regression Statistics								
Multiple R	0.983865948							
R Square	0.967992204							
Adjusted R Squar	0.961590644							
Standard Error	597974.6982							
Observations	7							
ANOVA								
	df	*SS*	*MS*	*F*	*Significance F*			
Regression	1	5.40694E+13	5.4069E+13	151.211941	6.2958E-05			
Residual	5	1.78787E+12	3.5757E+11					
Total	6	5.58573E+13						
	Coefficients	*Standard Error*	*t Stat*	*P-value*	*Lower 95%*	*Upper 95%*	*ower 95.0%*	*pper 95.0%*
Intercept	970215.5	40745.07574	2.38118282	0.06307248	-77170.8342	2017601.834	-77170.83	2017602
X Variable 1	2.77925E+12	2.26013E+11	12.2968265	6.2958E-05	2.1983E+12	3.36023E+12	2.198E+12	3.36E+12

LOD for OH^- is 3* standard error/co-efficient = 4.83×10^{-8} M

Figure 33S: calculation of LOD for OH^- using the regression method

35. Recyclability of the MOF for Al^{3+}, Fe^{3+}, OH^- ions

For Al^{3+} ions

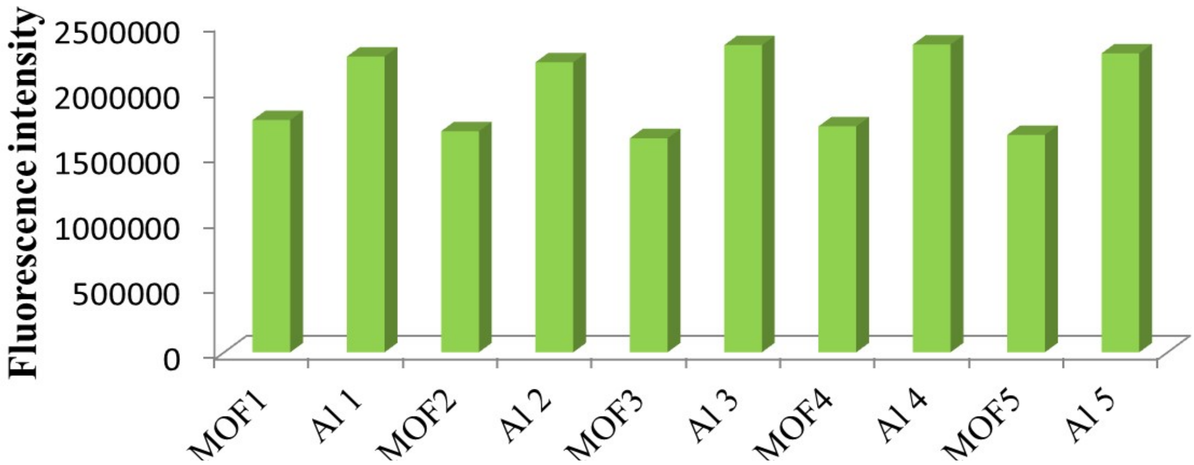

For Fe^{3+} ions

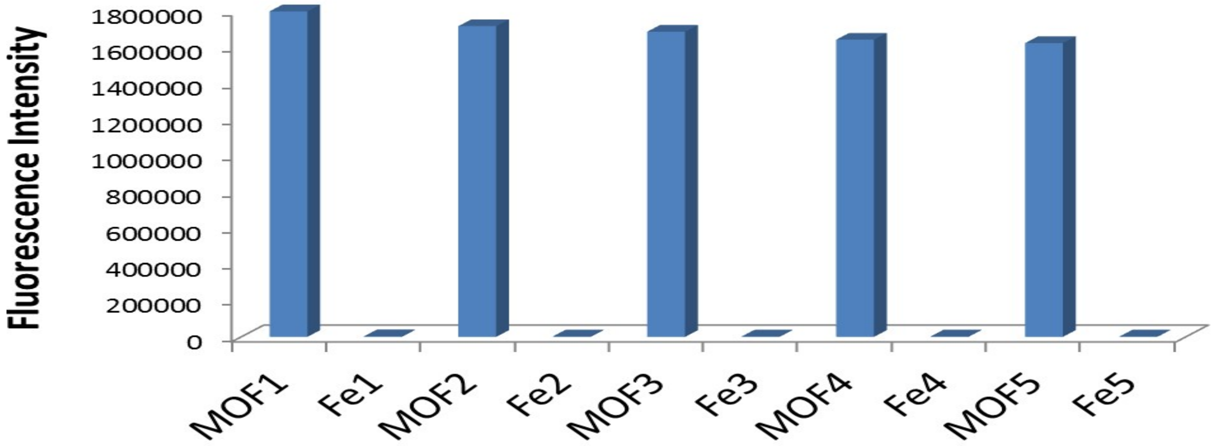

For OH^- ions

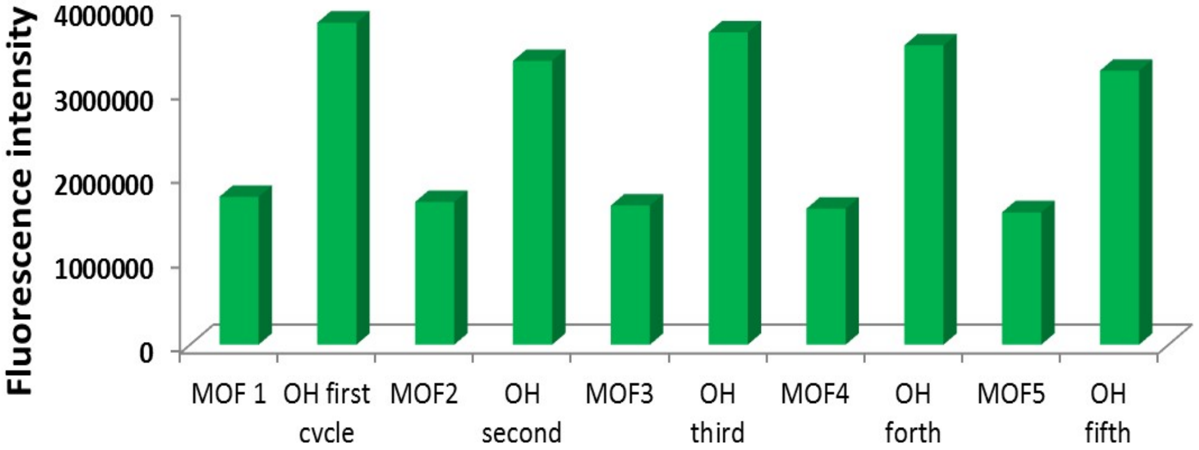

Figure 34S: The recyclability of the MOF up to 5th cycle for Al^{3+}, Fe^{3+}, OH^- ions

36. Time dependent photoluminescence

Al^{3+} ions

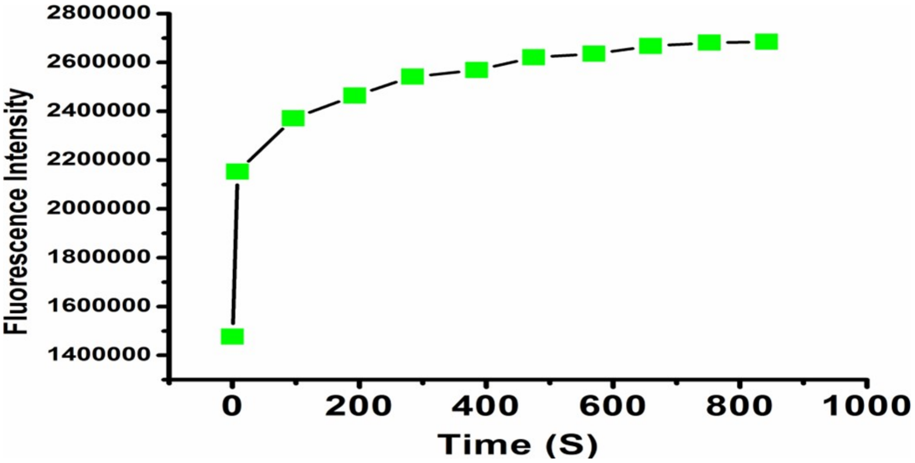

Fe^{3+} ions

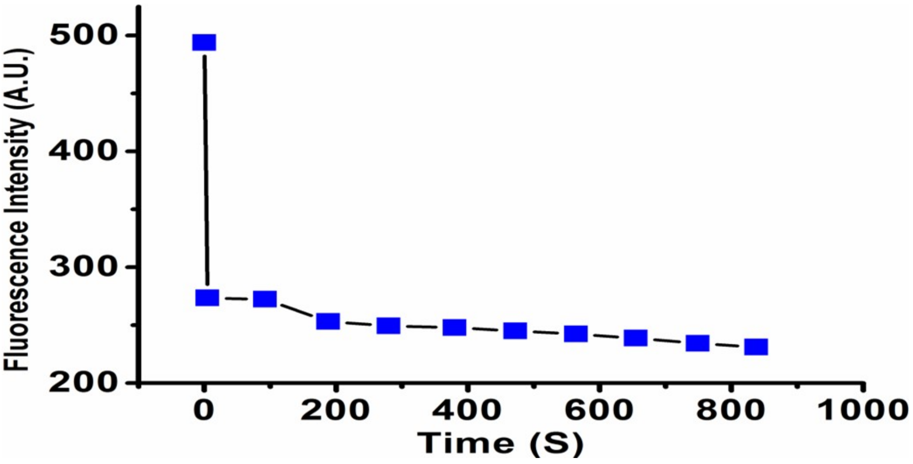

OH^- ions

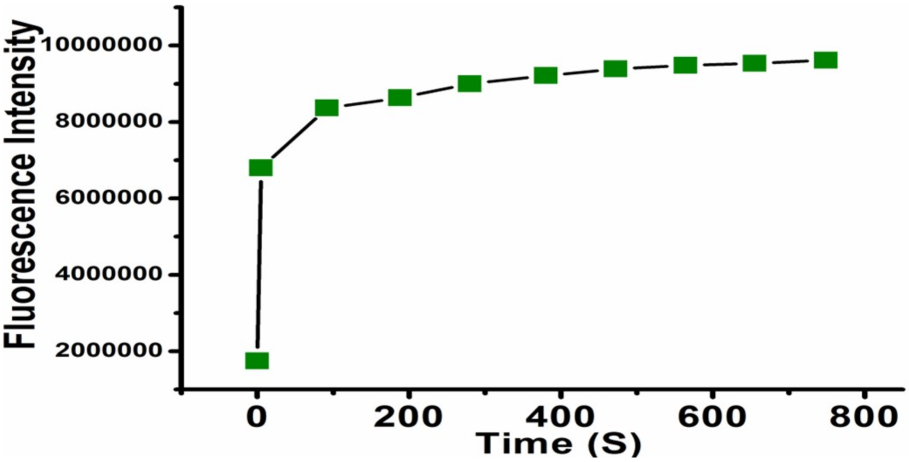

Figure 35S: the time dependent PL measuring the exposure time for Al^{3+}, Fe^{3+}, OH^- ions

37. Zeta potential of MOF in presence of pH solutions and OH^- ions

MOF in pH-7

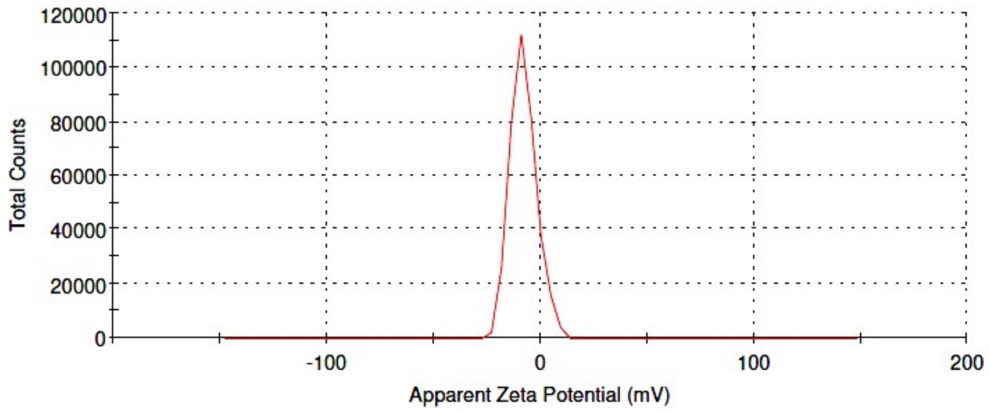

MOF in pH-9

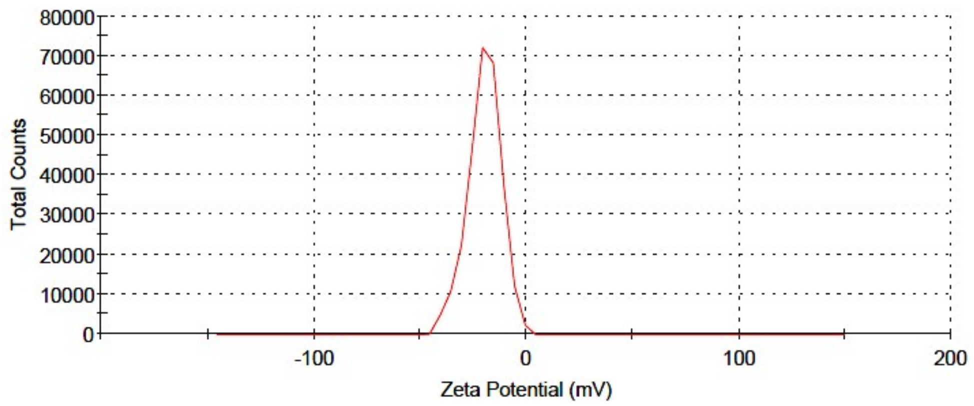

MOF in OH^- ions

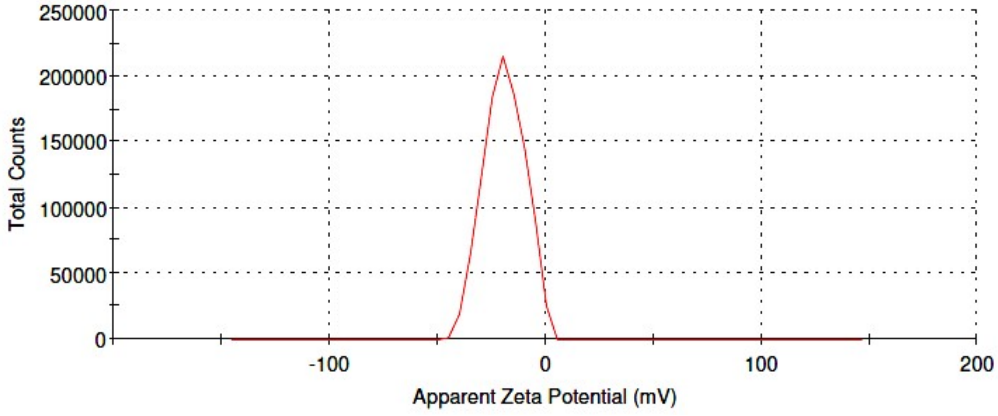

Figure 36S: Zeta potential of MOF in different pH solutions and OH^- ions

38. EDX of MOF recovered from metal ion solution of MOF

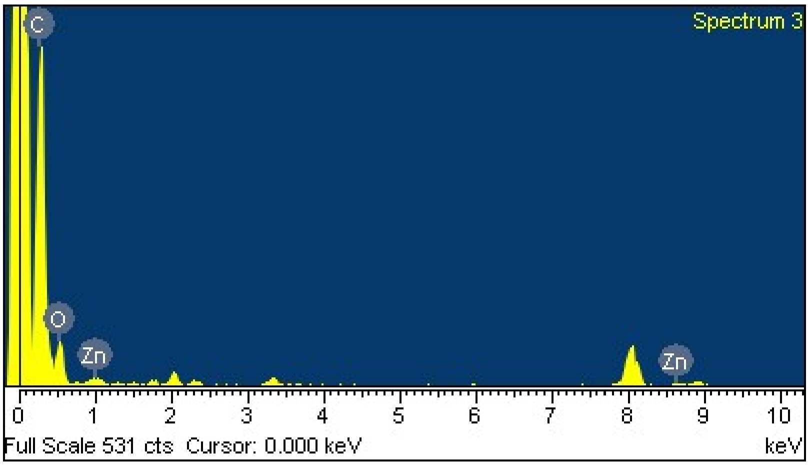

Element	Weight%	Atomic%
C K	60.01	62.51
O K	18.15	17.14
Zn K	21.84	20.35
Totals	100.00	

Figure 37S: EDX of MOF recovered from Al^{3+}@MOF
